# Supplementary material for: Genome-wide identification, characterization and gene expression of BES1 transcription factor family in grapevine (Vitis vinifera L.)
Source: Sci Rep. 2023 Jan 5;13:240. doi: 10.1038/s41598-022-24407-y (PMC9816167; doi:10.1038/s41598-022-24407-y)
Supplement: Supplementary file 3 — Supplementary Information. [file 41598_2022_24407_MOESM3_ESM.zip › Vvi_Vvi/Vitis_vinifera.PN40024.v4.dna_sm.toplevel.fa.vs.Vitis_vinifera.PN40024.v4.dna_sm.toplevel.fa.html/4.html]

|  |  |  |  |  |  |  |  |  |  |  |  |  |  |
| --- | --- | --- | --- | --- | --- | --- | --- | --- | --- | --- | --- | --- | --- |
| Duplication depth | Reference chromosome | Collinear blocks | | | | | | | | | | | |
| 0 | Vitvi04g04000\_t001 |  |  |  |  |  |  |  |  |  |  |  |  |
| 1 | Vitvi04g00001\_t001 |  | Vitvi11g00276\_t001 |  |  |  |  |  |  |  |  |  |  |
| 1 | Vitvi04g04001\_t001 |  | \| \| |  |  |  |  |  |  |  |  |  |  |
| 1 | Vitvi04g04002\_t001 |  | \| \| |  |  |  |  |  |  |  |  |  |  |
| 2 | Vitvi04g00005\_t001 |  | Vitvi11g00275\_t001 |  | Vitvi09g00307\_t001 |  |  |  |  |  |  |  |  |
| 2 | Vitvi04g00006\_t001 |  | \| \| |  | Vitvi09g00306\_t002 |  |  |  |  |  |  |  |  |
| 2 | Vitvi04g04003\_t001 |  | \| \| |  | \| \| |  |  |  |  |  |  |  |  |
| 2 | Vitvi04g00007\_t001 |  | \| \| |  | Vitvi09g00305\_t001 |  |  |  |  |  |  |  |  |
| 2 | Vitvi04g00009\_t001 |  | Vitvi11g00272\_t001 |  | Vitvi09g00301\_t001 |  |  |  |  |  |  |  |  |
| 2 | Vitvi04g00010\_t001 |  | \| \| |  | \| \| |  |  |  |  |  |  |  |  |
| 2 | Vitvi04g00011\_t001 |  | \| \| |  | \| \| |  |  |  |  |  |  |  |  |
| 2 | Vitvi04g00012\_t001 |  | Vitvi11g01392\_t001 |  | Vitvi09g00290\_t001 |  |  |  |  |  |  |  |  |
| 2 | Vitvi04g00013\_t002 |  | \| \| |  | Vitvi09g00281\_t001 |  |  |  |  |  |  |  |  |
| 1 | Vitvi04g01769\_t001 |  | \| \| |  |  |  |  |  |  |  |  |  |  |
| 1 | Vitvi04g01770\_t001 |  | \| \| |  |  |  |  |  |  |  |  |  |  |
| 1 | Vitvi04g00014\_t001 |  | \| \| |  |  |  |  |  |  |  |  |  |  |
| 1 | Vitvi04g04004\_t001 |  | \| \| |  |  |  |  |  |  |  |  |  |  |
| 1 | Vitvi04g04005\_t001 |  | \| \| |  |  |  |  |  |  |  |  |  |  |
| 1 | Vitvi04g04006\_t001 |  | \| \| |  |  |  |  |  |  |  |  |  |  |
| 1 | Vitvi04g00016\_t001 |  | \| \| |  |  |  |  |  |  |  |  |  |  |
| 1 | Vitvi04g04007\_t001 |  | \| \| |  |  |  |  |  |  |  |  |  |  |
| 1 | Vitvi04g00017\_t001 |  | \| \| |  |  |  |  |  |  |  |  |  |  |
| 1 | Vitvi04g04008\_t001 |  | \| \| |  |  |  |  |  |  |  |  |  |  |
| 1 | Vitvi04g00021\_t001 |  | Vitvi11g00261\_t001 |  |  |  |  |  |  |  |  |  |  |
| 1 | Vitvi04g00022\_t001 |  | \| \| |  |  |  |  |  |  |  |  |  |  |
| 1 | Vitvi04g04009\_t001 |  | \| \| |  |  |  |  |  |  |  |  |  |  |
| 1 | Vitvi04g04010\_t001 |  | \| \| |  |  |  |  |  |  |  |  |  |  |
| 1 | Vitvi04g04011\_t001 |  | \| \| |  |  |  |  |  |  |  |  |  |  |
| 1 | Vitvi04g04012\_t001 |  | \| \| |  |  |  |  |  |  |  |  |  |  |
| 1 | Vitvi04g04013\_t001 |  | \| \| |  |  |  |  |  |  |  |  |  |  |
| 1 | Vitvi04g04014\_t001 |  | \| \| |  |  |  |  |  |  |  |  |  |  |
| 1 | Vitvi04g00029\_t001 |  | \| \| |  |  |  |  |  |  |  |  |  |  |
| 1 | Vitvi04g04015\_t001 |  | \| \| |  |  |  |  |  |  |  |  |  |  |
| 1 | Vitvi04g04016\_t001 |  | \| \| |  |  |  |  |  |  |  |  |  |  |
| 1 | Vitvi04g04017\_t001 |  | \| \| |  |  |  |  |  |  |  |  |  |  |
| 1 | Vitvi04g04018\_t001 |  | \| \| |  |  |  |  |  |  |  |  |  |  |
| 1 | Vitvi04g00031\_t001 |  | \| \| |  |  |  |  |  |  |  |  |  |  |
| 1 | Vitvi04g04019\_t001 |  | \| \| |  |  |  |  |  |  |  |  |  |  |
| 1 | Vitvi04g00033\_t001 |  | \| \| |  |  |  |  |  |  |  |  |  |  |
| 1 | Vitvi04g00034\_t001 |  | \| \| |  |  |  |  |  |  |  |  |  |  |
| 1 | Vitvi04g00035\_t001 |  | \| \| |  |  |  |  |  |  |  |  |  |  |
| 1 | Vitvi04g00036\_t001 |  | Vitvi11g00251\_t001 |  |  |  |  |  |  |  |  |  |  |
| 1 | Vitvi04g01775\_t001 |  | \| \| |  |  |  |  |  |  |  |  |  |  |
| 1 | Vitvi04g00037\_t001 |  | \| \| |  |  |  |  |  |  |  |  |  |  |
| 1 | Vitvi04g00038\_t001 |  | Vitvi11g00247\_t001 |  |  |  |  |  |  |  |  |  |  |
| 1 | Vitvi04g00039\_t002 |  | \| \| |  |  |  |  |  |  |  |  |  |  |
| 1 | Vitvi04g00040\_t003 |  | Vitvi11g00237\_t001 |  |  |  |  |  |  |  |  |  |  |
| 1 | Vitvi04g00041\_t001 |  | \| \| |  |  |  |  |  |  |  |  |  |  |
| 1 | Vitvi04g00042\_t001 |  | \| \| |  |  |  |  |  |  |  |  |  |  |
| 1 | Vitvi04g00043\_t001.1.6037826d |  | \| \| |  |  |  |  |  |  |  |  |  |  |
| 1 | Vitvi04g00044\_t001 |  | \| \| |  |  |  |  |  |  |  |  |  |  |
| 1 | Vitvi04g01777\_t001 |  | \| \| |  |  |  |  |  |  |  |  |  |  |
| 1 | Vitvi04g00045\_t001 |  | Vitvi11g01386\_t001 |  |  |  |  |  |  |  |  |  |  |
| 1 | Vitvi04g00046\_t001 |  | \| \| |  |  |  |  |  |  |  |  |  |  |
| 1 | Vitvi04g04020\_t001 |  | \| \| |  |  |  |  |  |  |  |  |  |  |
| 1 | Vitvi04g00047\_t001 |  | Vitvi11g00229\_t001 |  |  |  |  |  |  |  |  |  |  |
| 1 | Vitvi04g04021\_t001 |  | \| \| |  |  |  |  |  |  |  |  |  |  |
| 1 | Vitvi04g00049\_t001 |  | Vitvi11g00228\_t001 |  |  |  |  |  |  |  |  |  |  |
| 2 | Vitvi04g01778\_t003 |  | Vitvi11g01381\_t001 |  | Vitvi09g01550\_t006 |  |  |  |  |  |  |  |  |
| 2 | Vitvi04g00050\_t002 |  | \| \| |  | \| \| |  |  |  |  |  |  |  |  |
| 2 | Vitvi04g00051\_t001 |  | \| \| |  | Vitvi09g00226\_t001 |  |  |  |  |  |  |  |  |
| 2 | Vitvi04g04022\_t001 |  | \| \| |  | \| \| |  |  |  |  |  |  |  |  |
| 2 | Vitvi04g00052\_t001 |  | \| \| |  | Vitvi09g00225\_t001 |  |  |  |  |  |  |  |  |
| 2 | Vitvi04g01779\_t001 |  | \| \| |  | \| \| |  |  |  |  |  |  |  |  |
| 2 | Vitvi04g00053\_t001 |  | \| \| |  | \| \| |  |  |  |  |  |  |  |  |
| 2 | Vitvi04g01780\_t001 |  | \| \| |  | \| \| |  |  |  |  |  |  |  |  |
| 2 | Vitvi04g00055\_t001 |  | \| \| |  | \| \| |  |  |  |  |  |  |  |  |
| 2 | Vitvi04g00056\_t001 |  | \| \| |  | Vitvi09g00223\_t001 |  |  |  |  |  |  |  |  |
| 2 | Vitvi04g01781\_t001 |  | \| \| |  | \| \| |  |  |  |  |  |  |  |  |
| 2 | Vitvi04g04023\_t001 |  | \| \| |  | \| \| |  |  |  |  |  |  |  |  |
| 2 | Vitvi04g04024\_t001 |  | \| \| |  | \| \| |  |  |  |  |  |  |  |  |
| 2 | Vitvi04g00059\_t001 |  | \| \| |  | \| \| |  |  |  |  |  |  |  |  |
| 2 | Vitvi04g00060\_t001 |  | \| \| |  | \| \| |  |  |  |  |  |  |  |  |
| 2 | Vitvi04g04025\_t001 |  | \| \| |  | \| \| |  |  |  |  |  |  |  |  |
| 2 | Vitvi04g00062\_t001 |  | \| \| |  | \| \| |  |  |  |  |  |  |  |  |
| 2 | Vitvi04g00063\_t001 |  | \| \| |  | \| \| |  |  |  |  |  |  |  |  |
| 2 | Vitvi04g01782\_t001 |  | \| \| |  | \| \| |  |  |  |  |  |  |  |  |
| 2 | Vitvi04g00064\_t001 |  | \| \| |  | Vitvi09g01545\_t001 |  |  |  |  |  |  |  |  |
| 2 | Vitvi04g00065\_t001 |  | \| \| |  | \| \| |  |  |  |  |  |  |  |  |
| 2 | Vitvi04g04026\_t001 |  | \| \| |  | Vitvi09g00209\_t001 |  |  |  |  |  |  |  |  |
| 2 | Vitvi04g00066\_t001 |  | \| \| |  | \| \| |  |  |  |  |  |  |  |  |
| 2 | Vitvi04g00067\_t001 |  | \| \| |  | \| \| |  |  |  |  |  |  |  |  |
| 2 | Vitvi04g00068\_t001 |  | Vitvi11g00210\_t001 |  | Vitvi09g00208\_t001 |  |  |  |  |  |  |  |  |
| 2 | Vitvi04g00069\_t001 |  | Vitvi11g00206\_t001 |  | \| \| |  |  |  |  |  |  |  |  |
| 2 | Vitvi04g00070\_t001 |  | Vitvi11g00203\_t001 |  | \| \| |  |  |  |  |  |  |  |  |
| 2 | Vitvi04g00071\_t001 |  | Vitvi11g00202\_t001 |  | Vitvi09g01542\_t002 |  |  |  |  |  |  |  |  |
| 1 | Vitvi04g00072\_t001 |  | \| \| |  |  |  |  |  |  |  |  |  |  |
| 1 | Vitvi04g04027\_t001 |  | \| \| |  |  |  |  |  |  |  |  |  |  |
| 1 | Vitvi04g00075\_t001 |  | \| \| |  |  |  |  |  |  |  |  |  |  |
| 1 | Vitvi04g00076\_t001 |  | \| \| |  |  |  |  |  |  |  |  |  |  |
| 1 | Vitvi04g00077\_t001 |  | \| \| |  |  |  |  |  |  |  |  |  |  |
| 1 | Vitvi04g04028\_t001 |  | \| \| |  |  |  |  |  |  |  |  |  |  |
| 1 | Vitvi04g04029\_t001 |  | \| \| |  |  |  |  |  |  |  |  |  |  |
| 1 | Vitvi04g04030\_t001 |  | \| \| |  |  |  |  |  |  |  |  |  |  |
| 1 | Vitvi04g00078\_t001 |  | \| \| |  |  |  |  |  |  |  |  |  |  |
| 1 | Vitvi04g01784\_t001 |  | \| \| |  |  |  |  |  |  |  |  |  |  |
| 1 | Vitvi04g00080\_t001 |  | \| \| |  |  |  |  |  |  |  |  |  |  |
| 1 | Vitvi04g00081\_t001 |  | \| \| |  |  |  |  |  |  |  |  |  |  |
| 2 | Vitvi04g04031\_t001 |  | \| \| |  | Vitvi11g00152\_t001 |  |  |  |  |  |  |  |  |
| 2 | Vitvi04g04032\_t001 |  | \| \| |  | \| \| |  |  |  |  |  |  |  |  |
| 2 | Vitvi04g00083\_t001 |  | \| \| |  | \| \| |  |  |  |  |  |  |  |  |
| 2 | Vitvi04g01786\_t001 |  | \| \| |  | Vitvi11g00154\_t001 |  |  |  |  |  |  |  |  |
| 2 | Vitvi04g00084\_t001 |  | \| \| |  | \| \| |  |  |  |  |  |  |  |  |
| 2 | Vitvi04g00085\_t001 |  | \| \| |  | \| \| |  |  |  |  |  |  |  |  |
| 2 | Vitvi04g01787\_t001 |  | \| \| |  | \| \| |  |  |  |  |  |  |  |  |
| 2 | Vitvi04g00086\_t001 |  | \| \| |  | \| \| |  |  |  |  |  |  |  |  |
| 2 | Vitvi04g00087\_t001 |  | Vitvi11g00201\_t001 |  | \| \| |  |  |  |  |  |  |  |  |
| 2 | Vitvi04g00088\_t001 |  | \| \| |  | Vitvi11g00156\_t001 |  |  |  |  |  |  |  |  |
| 2 | Vitvi04g00089\_t001 |  | \| \| |  | \| \| |  |  |  |  |  |  |  |  |
| 2 | Vitvi04g00090\_t001 |  | \| \| |  | \| \| |  |  |  |  |  |  |  |  |
| 2 | Vitvi04g00091\_t001 |  | \| \| |  | \| \| |  |  |  |  |  |  |  |  |
| 2 | Vitvi04g00092\_t001 |  | \| \| |  | Vitvi11g00159\_t001 |  |  |  |  |  |  |  |  |
| 2 | Vitvi04g04033\_t001 |  | \| \| |  | \| \| |  |  |  |  |  |  |  |  |
| 2 | Vitvi04g01788\_t001 |  | \| \| |  | \| \| |  |  |  |  |  |  |  |  |
| 2 | Vitvi04g00093\_t001 |  | \| \| |  | \| \| |  |  |  |  |  |  |  |  |
| 2 | Vitvi04g00094\_t001 |  | \| \| |  | \| \| |  |  |  |  |  |  |  |  |
| 2 | Vitvi04g00095\_t001 |  | \| \| |  | \| \| |  |  |  |  |  |  |  |  |
| 2 | Vitvi04g00096\_t001 |  | \| \| |  | \| \| |  |  |  |  |  |  |  |  |
| 2 | Vitvi04g04034\_t001 |  | \| \| |  | \| \| |  |  |  |  |  |  |  |  |
| 2 | Vitvi04g00098\_t001 |  | \| \| |  | \| \| |  |  |  |  |  |  |  |  |
| 2 | Vitvi04g04035\_t001 |  | \| \| |  | \| \| |  |  |  |  |  |  |  |  |
| 2 | Vitvi04g00099\_t001 |  | \| \| |  | Vitvi11g01369\_t001 |  |  |  |  |  |  |  |  |
| 2 | Vitvi04g04036\_t001 |  | \| \| |  | Vitvi11g00168\_t001 |  |  |  |  |  |  |  |  |
| 2 | Vitvi04g00103\_t001 |  | \| \| |  | \| \| |  |  |  |  |  |  |  |  |
| 2 | Vitvi04g01768\_t001 |  | \| \| |  | \| \| |  |  |  |  |  |  |  |  |
| 2 | Vitvi04g00104\_t001 |  | \| \| |  | \| \| |  |  |  |  |  |  |  |  |
| 2 | Vitvi04g00105\_t001 |  | \| \| |  | \| \| |  |  |  |  |  |  |  |  |
| 2 | Vitvi04g00106\_t001 |  | \| \| |  | \| \| |  |  |  |  |  |  |  |  |
| 2 | Vitvi04g00107\_t001 |  | \| \| |  | Vitvi11g01372\_t001 |  |  |  |  |  |  |  |  |
| 2 | Vitvi04g00109\_t001 |  | \| \| |  | \| \| |  |  |  |  |  |  |  |  |
| 2 | Vitvi04g00110\_t001 |  | \| \| |  | \| \| |  |  |  |  |  |  |  |  |
| 2 | Vitvi04g01789\_t001 |  | \| \| |  | \| \| |  |  |  |  |  |  |  |  |
| 2 | Vitvi04g00111\_t001 |  | Vitvi11g00180\_t001 |  | Vitvi11g00180\_t001 |  |  |  |  |  |  |  |  |
| 1 | Vitvi04g00112\_t001 |  |  |  | Vitvi11g00186\_t001 |  |  |  |  |  |  |  |  |
| 0 | Vitvi04g00113\_t001 |  |  |  |  |  |  |  |  |  |  |  |  |
| 0 | Vitvi04g00114\_t001 |  |  |  |  |  |  |  |  |  |  |  |  |
| 0 | Vitvi04g00115\_t002 |  |  |  |  |  |  |  |  |  |  |  |  |
| 0 | Vitvi04g00116\_t001 |  |  |  |  |  |  |  |  |  |  |  |  |
| 0 | Vitvi04g00117\_t001 |  |  |  |  |  |  |  |  |  |  |  |  |
| 0 | Vitvi04g04037\_t001 |  |  |  |  |  |  |  |  |  |  |  |  |
| 1 | Vitvi04g00118\_t001 |  | Vitvi11g00142\_t001 |  |  |  |  |  |  |  |  |  |  |
| 1 | Vitvi04g00119\_t001 |  | \| \| |  |  |  |  |  |  |  |  |  |  |
| 1 | Vitvi04g00121\_t001 |  | Vitvi11g00140\_t001 |  |  |  |  |  |  |  |  |  |  |
| 1 | Vitvi04g01791\_t001 |  | \| \| |  |  |  |  |  |  |  |  |  |  |
| 1 | Vitvi04g00124\_t001 |  | Vitvi11g00139\_t001 |  |  |  |  |  |  |  |  |  |  |
| 1 | Vitvi04g00125\_t001 |  | \| \| |  |  |  |  |  |  |  |  |  |  |
| 1 | Vitvi04g00127\_t001 |  | Vitvi11g00137\_t002 |  |  |  |  |  |  |  |  |  |  |
| 1 | Vitvi04g00128\_t001 |  | \| \| |  |  |  |  |  |  |  |  |  |  |
| 1 | Vitvi04g00129\_t001 |  | \| \| |  |  |  |  |  |  |  |  |  |  |
| 1 | Vitvi04g00130\_t001 |  | \| \| |  |  |  |  |  |  |  |  |  |  |
| 1 | Vitvi04g00131\_t001 |  | \| \| |  |  |  |  |  |  |  |  |  |  |
| 1 | Vitvi04g00132\_t002 |  | Vitvi11g00127\_t002 |  |  |  |  |  |  |  |  |  |  |
| 1 | Vitvi04g00133\_t001 |  | \| \| |  |  |  |  |  |  |  |  |  |  |
| 1 | Vitvi04g00134\_t001 |  | \| \| |  |  |  |  |  |  |  |  |  |  |
| 1 | Vitvi04g01792\_t001 |  | \| \| |  |  |  |  |  |  |  |  |  |  |
| 1 | Vitvi04g01793\_t001 |  | \| \| |  |  |  |  |  |  |  |  |  |  |
| 1 | Vitvi04g01794\_t001 |  | \| \| |  |  |  |  |  |  |  |  |  |  |
| 1 | Vitvi04g01795\_t001 |  | \| \| |  |  |  |  |  |  |  |  |  |  |
| 1 | Vitvi04g04038\_t001 |  | \| \| |  |  |  |  |  |  |  |  |  |  |
| 1 | Vitvi04g04039\_t001 |  | \| \| |  |  |  |  |  |  |  |  |  |  |
| 1 | Vitvi04g01796\_t001 |  | \| \| |  |  |  |  |  |  |  |  |  |  |
| 1 | Vitvi04g01797\_t001 |  | \| \| |  |  |  |  |  |  |  |  |  |  |
| 1 | Vitvi04g01799\_t001 |  | \| \| |  |  |  |  |  |  |  |  |  |  |
| 1 | Vitvi04g04040\_t001 |  | \| \| |  |  |  |  |  |  |  |  |  |  |
| 1 | Vitvi04g04041\_t001 |  | \| \| |  |  |  |  |  |  |  |  |  |  |
| 1 | Vitvi04g01802\_t001 |  | \| \| |  |  |  |  |  |  |  |  |  |  |
| 1 | Vitvi04g00135\_t001 |  | \| \| |  |  |  |  |  |  |  |  |  |  |
| 1 | Vitvi04g04042\_t001 |  | \| \| |  |  |  |  |  |  |  |  |  |  |
| 1 | Vitvi04g04043\_t001 |  | \| \| |  |  |  |  |  |  |  |  |  |  |
| 1 | Vitvi04g00136\_t001 |  | \| \| |  |  |  |  |  |  |  |  |  |  |
| 1 | Vitvi04g01803\_t001 |  | \| \| |  |  |  |  |  |  |  |  |  |  |
| 1 | Vitvi04g01804\_t001 |  | \| \| |  |  |  |  |  |  |  |  |  |  |
| 1 | Vitvi04g00137\_t001 |  | \| \| |  |  |  |  |  |  |  |  |  |  |
| 1 | Vitvi04g00138\_t001 |  | \| \| |  |  |  |  |  |  |  |  |  |  |
| 1 | Vitvi04g04044\_t001 |  | \| \| |  |  |  |  |  |  |  |  |  |  |
| 1 | Vitvi04g04045\_t001 |  | Vitvi11g01360\_t001 |  |  |  |  |  |  |  |  |  |  |
| 1 | Vitvi04g00139\_t001 |  | Vitvi11g04026\_t001 |  |  |  |  |  |  |  |  |  |  |
| 2 | Vitvi04g01805\_t001 |  | \| \| |  | Vitvi09g00134\_t001 |  |  |  |  |  |  |  |  |
| 2 | Vitvi04g00140\_t001 |  | Vitvi11g00113\_t001 |  | Vitvi09g01518\_t001 |  |  |  |  |  |  |  |  |
| 2 | Vitvi04g04046\_t001 |  | \| \| |  | \| \| |  |  |  |  |  |  |  |  |
| 2 | Vitvi04g00141\_t001 |  | \| \| |  | \| \| |  |  |  |  |  |  |  |  |
| 2 | Vitvi04g00142\_t001 |  | \| \| |  | \| \| |  |  |  |  |  |  |  |  |
| 2 | Vitvi04g00143\_t001 |  | \| \| |  | \| \| |  |  |  |  |  |  |  |  |
| 2 | Vitvi04g00144\_t001 |  | \| \| |  | \| \| |  |  |  |  |  |  |  |  |
| 2 | Vitvi04g00145\_t001 |  | Vitvi11g00106\_t001 |  | Vitvi09g00126\_t001 |  |  |  |  |  |  |  |  |
| 2 | Vitvi04g00146\_t001 |  | \| \| |  | \| \| |  |  |  |  |  |  |  |  |
| 2 | Vitvi04g00147\_t001 |  | Vitvi11g01346\_t001 |  | \| \| |  |  |  |  |  |  |  |  |
| 2 | Vitvi04g01806\_t001 |  | Vitvi11g01345\_t001 |  | \| \| |  |  |  |  |  |  |  |  |
| 2 | Vitvi04g00148\_t001 |  | \| \| |  | \| \| |  |  |  |  |  |  |  |  |
| 2 | Vitvi04g00149\_t001 |  | Vitvi11g00103\_t001 |  | Vitvi09g00119\_t001 |  |  |  |  |  |  |  |  |
| 2 | Vitvi04g00150\_t001 |  | \| \| |  | \| \| |  |  |  |  |  |  |  |  |
| 2 | Vitvi04g00151\_t001 |  | \| \| |  | \| \| |  |  |  |  |  |  |  |  |
| 2 | Vitvi04g00152\_t001 |  | \| \| |  | \| \| |  |  |  |  |  |  |  |  |
| 2 | Vitvi04g04047\_t001 |  | \| \| |  | \| \| |  |  |  |  |  |  |  |  |
| 2 | Vitvi04g00153\_t001 |  | Vitvi11g00097\_t001 |  | \| \| |  |  |  |  |  |  |  |  |
| 2 | Vitvi04g00155\_t001 |  | \| \| |  | Vitvi09g00112\_t001 |  |  |  |  |  |  |  |  |
| 2 | Vitvi04g00156\_t001 |  | \| \| |  | \| \| |  |  |  |  |  |  |  |  |
| 2 | Vitvi04g00157\_t001 |  | \| \| |  | \| \| |  |  |  |  |  |  |  |  |
| 2 | Vitvi04g00158\_t001 |  | \| \| |  | \| \| |  |  |  |  |  |  |  |  |
| 2 | Vitvi04g01808\_t001 |  | \| \| |  | \| \| |  |  |  |  |  |  |  |  |
| 2 | Vitvi04g00159\_t001 |  | Vitvi11g00096\_t001 |  | \| \| |  |  |  |  |  |  |  |  |
| 2 | Vitvi04g00160\_t001 |  | \| \| |  | Vitvi09g00110\_t001 |  |  |  |  |  |  |  |  |
| 1 | Vitvi04g00161\_t001 |  | \| \| |  |  |  |  |  |  |  |  |  |  |
| 1 | Vitvi04g00162\_t001 |  | \| \| |  |  |  |  |  |  |  |  |  |  |
| 1 | Vitvi04g00163\_t001 |  | \| \| |  |  |  |  |  |  |  |  |  |  |
| 1 | Vitvi04g00164\_t001 |  | \| \| |  |  |  |  |  |  |  |  |  |  |
| 1 | Vitvi04g00165\_t001 |  | Vitvi11g00085\_t001 |  |  |  |  |  |  |  |  |  |  |
| 1 | Vitvi04g01809\_t001 |  | \| \| |  |  |  |  |  |  |  |  |  |  |
| 1 | Vitvi04g00166\_t001 |  | \| \| |  |  |  |  |  |  |  |  |  |  |
| 1 | Vitvi04g00167\_t001 |  | \| \| |  |  |  |  |  |  |  |  |  |  |
| 1 | Vitvi04g00168\_t001 |  | \| \| |  |  |  |  |  |  |  |  |  |  |
| 1 | Vitvi04g00171\_t001 |  | \| \| |  |  |  |  |  |  |  |  |  |  |
| 1 | Vitvi04g00172\_t001 |  | \| \| |  |  |  |  |  |  |  |  |  |  |
| 1 | Vitvi04g00173\_t001 |  | Vitvi11g00080\_t001 |  |  |  |  |  |  |  |  |  |  |
| 1 | Vitvi04g00174\_t001 |  | \| \| |  |  |  |  |  |  |  |  |  |  |
| 1 | Vitvi04g01810\_t001 |  | \| \| |  |  |  |  |  |  |  |  |  |  |
| 1 | Vitvi04g00175\_t002 |  | \| \| |  |  |  |  |  |  |  |  |  |  |
| 1 | Vitvi04g00176\_t002 |  | \| \| |  |  |  |  |  |  |  |  |  |  |
| 1 | Vitvi04g00178\_t001 |  | \| \| |  |  |  |  |  |  |  |  |  |  |
| 1 | Vitvi04g00179\_t001 |  | Vitvi11g01336\_t001 |  |  |  |  |  |  |  |  |  |  |
| 1 | Vitvi04g00181\_t001 |  | \| \| |  |  |  |  |  |  |  |  |  |  |
| 1 | Vitvi04g01813\_t001 |  | Vitvi11g00068\_t001 |  |  |  |  |  |  |  |  |  |  |
| 1 | Vitvi04g00182\_t001 |  | Vitvi11g01331\_t001 |  |  |  |  |  |  |  |  |  |  |
| 1 | Vitvi04g00183\_t001 |  | \| \| |  |  |  |  |  |  |  |  |  |  |
| 1 | Vitvi04g00184\_t001 |  | \| \| |  |  |  |  |  |  |  |  |  |  |
| 1 | Vitvi04g00185\_t001 |  | \| \| |  |  |  |  |  |  |  |  |  |  |
| 1 | Vitvi04g00186\_t001 |  | \| \| |  |  |  |  |  |  |  |  |  |  |
| 1 | Vitvi04g00187\_t001 |  | \| \| |  |  |  |  |  |  |  |  |  |  |
| 1 | Vitvi04g01815\_t001 |  | \| \| |  |  |  |  |  |  |  |  |  |  |
| 1 | Vitvi04g04048\_t001 |  | \| \| |  |  |  |  |  |  |  |  |  |  |
| 1 | Vitvi04g01817\_t002 |  | \| \| |  |  |  |  |  |  |  |  |  |  |
| 1 | Vitvi04g00189\_t001 |  | \| \| |  |  |  |  |  |  |  |  |  |  |
| 1 | Vitvi04g00190\_t001 |  | Vitvi11g00045\_t001 |  |  |  |  |  |  |  |  |  |  |
| 1 | Vitvi04g04049\_t001 |  | \| \| |  |  |  |  |  |  |  |  |  |  |
| 1 | Vitvi04g00192\_t001 |  | \| \| |  |  |  |  |  |  |  |  |  |  |
| 1 | Vitvi04g00193\_t001 |  | Vitvi11g00039\_t001 |  |  |  |  |  |  |  |  |  |  |
| 0 | Vitvi04g00194\_t001 |  |  |  |  |  |  |  |  |  |  |  |  |
| 0 | Vitvi04g00195\_t002 |  |  |  |  |  |  |  |  |  |  |  |  |
| 0 | Vitvi04g00196\_t001 |  |  |  |  |  |  |  |  |  |  |  |  |
| 0 | Vitvi04g04050\_t001 |  |  |  |  |  |  |  |  |  |  |  |  |
| 0 | Vitvi04g00197\_t001 |  |  |  |  |  |  |  |  |  |  |  |  |
| 0 | Vitvi04g00198\_t001 |  |  |  |  |  |  |  |  |  |  |  |  |
| 0 | Vitvi04g00199\_t001 |  |  |  |  |  |  |  |  |  |  |  |  |
| 0 | Vitvi04g00200\_t001 |  |  |  |  |  |  |  |  |  |  |  |  |
| 0 | Vitvi04g01819\_t001 |  |  |  |  |  |  |  |  |  |  |  |  |
| 0 | Vitvi04g00201\_t001 |  |  |  |  |  |  |  |  |  |  |  |  |
| 0 | Vitvi04g01820\_t001 |  |  |  |  |  |  |  |  |  |  |  |  |
| 0 | Vitvi04g04051\_t001 |  |  |  |  |  |  |  |  |  |  |  |  |
| 0 | Vitvi04g01821\_t001 |  |  |  |  |  |  |  |  |  |  |  |  |
| 0 | Vitvi04g00202\_t001 |  |  |  |  |  |  |  |  |  |  |  |  |
| 0 | Vitvi04g04052\_t001 |  |  |  |  |  |  |  |  |  |  |  |  |
| 0 | Vitvi04g00205\_t002 |  |  |  |  |  |  |  |  |  |  |  |  |
| 0 | Vitvi04g04053\_t001 |  |  |  |  |  |  |  |  |  |  |  |  |
| 0 | Vitvi04g00206\_t001 |  |  |  |  |  |  |  |  |  |  |  |  |
| 0 | Vitvi04g00209\_t002 |  |  |  |  |  |  |  |  |  |  |  |  |
| 0 | Vitvi04g00210\_t001 |  |  |  |  |  |  |  |  |  |  |  |  |
| 0 | Vitvi04g00211\_t001 |  |  |  |  |  |  |  |  |  |  |  |  |
| 0 | Vitvi04g00212\_t001 |  |  |  |  |  |  |  |  |  |  |  |  |
| 0 | Vitvi04g04054\_t001 |  |  |  |  |  |  |  |  |  |  |  |  |
| 0 | Vitvi04g00214\_t001 |  |  |  |  |  |  |  |  |  |  |  |  |
| 0 | Vitvi04g01824\_t001 |  |  |  |  |  |  |  |  |  |  |  |  |
| 0 | Vitvi04g00216\_t001 |  |  |  |  |  |  |  |  |  |  |  |  |
| 0 | Vitvi04g00217\_t001 |  |  |  |  |  |  |  |  |  |  |  |  |
| 0 | Vitvi04g04055\_t001 |  |  |  |  |  |  |  |  |  |  |  |  |
| 0 | Vitvi04g00219\_t001 |  |  |  |  |  |  |  |  |  |  |  |  |
| 0 | Vitvi04g00220\_t001 |  |  |  |  |  |  |  |  |  |  |  |  |
| 0 | Vitvi04g04056\_t001 |  |  |  |  |  |  |  |  |  |  |  |  |
| 0 | Vitvi04g00221\_t001 |  |  |  |  |  |  |  |  |  |  |  |  |
| 0 | Vitvi04g00223\_t001 |  |  |  |  |  |  |  |  |  |  |  |  |
| 0 | Vitvi04g00224\_t001 |  |  |  |  |  |  |  |  |  |  |  |  |
| 0 | Vitvi04g01825\_t001 |  |  |  |  |  |  |  |  |  |  |  |  |
| 0 | Vitvi04g00225\_t001 |  |  |  |  |  |  |  |  |  |  |  |  |
| 0 | Vitvi04g00226\_t001 |  |  |  |  |  |  |  |  |  |  |  |  |
| 0 | Vitvi04g04057\_t001 |  |  |  |  |  |  |  |  |  |  |  |  |
| 0 | Vitvi04g00228\_t001 |  |  |  |  |  |  |  |  |  |  |  |  |
| 0 | Vitvi04g04058\_t001 |  |  |  |  |  |  |  |  |  |  |  |  |
| 0 | Vitvi04g00229\_t001 |  |  |  |  |  |  |  |  |  |  |  |  |
| 0 | Vitvi04g01826\_t001 |  |  |  |  |  |  |  |  |  |  |  |  |
| 0 | Vitvi04g00230\_t001 |  |  |  |  |  |  |  |  |  |  |  |  |
| 0 | Vitvi04g00231\_t001 |  |  |  |  |  |  |  |  |  |  |  |  |
| 0 | Vitvi04g00232\_t001 |  |  |  |  |  |  |  |  |  |  |  |  |
| 0 | Vitvi04g01827\_t001 |  |  |  |  |  |  |  |  |  |  |  |  |
| 0 | Vitvi04g01828\_t001 |  |  |  |  |  |  |  |  |  |  |  |  |
| 0 | Vitvi04g00234\_t001 |  |  |  |  |  |  |  |  |  |  |  |  |
| 0 | Vitvi04g00235\_t001 |  |  |  |  |  |  |  |  |  |  |  |  |
| 0 | Vitvi04g00236\_t001 |  |  |  |  |  |  |  |  |  |  |  |  |
| 0 | Vitvi04g00237\_t001 |  |  |  |  |  |  |  |  |  |  |  |  |
| 0 | Vitvi04g04059\_t001 |  |  |  |  |  |  |  |  |  |  |  |  |
| 0 | Vitvi04g00239\_t001 |  |  |  |  |  |  |  |  |  |  |  |  |
| 0 | Vitvi04g01830\_t001 |  |  |  |  |  |  |  |  |  |  |  |  |
| 0 | Vitvi04g00240\_t001 |  |  |  |  |  |  |  |  |  |  |  |  |
| 0 | Vitvi04g00241\_t001 |  |  |  |  |  |  |  |  |  |  |  |  |
| 0 | Vitvi04g00242\_t001 |  |  |  |  |  |  |  |  |  |  |  |  |
| 0 | Vitvi04g00243\_t001 |  |  |  |  |  |  |  |  |  |  |  |  |
| 0 | Vitvi04g00244\_t001 |  |  |  |  |  |  |  |  |  |  |  |  |
| 0 | Vitvi04g01831\_t001 |  |  |  |  |  |  |  |  |  |  |  |  |
| 0 | Vitvi04g00247\_t001 |  |  |  |  |  |  |  |  |  |  |  |  |
| 0 | Vitvi04g00248\_t001 |  |  |  |  |  |  |  |  |  |  |  |  |
| 0 | Vitvi04g00249\_t001 |  |  |  |  |  |  |  |  |  |  |  |  |
| 0 | Vitvi04g00250\_t001 |  |  |  |  |  |  |  |  |  |  |  |  |
| 0 | Vitvi04g00251\_t001 |  |  |  |  |  |  |  |  |  |  |  |  |
| 0 | Vitvi04g00252\_t001 |  |  |  |  |  |  |  |  |  |  |  |  |
| 0 | Vitvi04g00253\_t003 |  |  |  |  |  |  |  |  |  |  |  |  |
| 0 | Vitvi04g00254\_t001 |  |  |  |  |  |  |  |  |  |  |  |  |
| 0 | Vitvi04g00255\_t001 |  |  |  |  |  |  |  |  |  |  |  |  |
| 0 | Vitvi04g00256\_t002 |  |  |  |  |  |  |  |  |  |  |  |  |
| 0 | Vitvi04g00257\_t001 |  |  |  |  |  |  |  |  |  |  |  |  |
| 0 | Vitvi04g00259\_t001 |  |  |  |  |  |  |  |  |  |  |  |  |
| 0 | Vitvi04g00260\_t001 |  |  |  |  |  |  |  |  |  |  |  |  |
| 0 | Vitvi04g00261\_t001 |  |  |  |  |  |  |  |  |  |  |  |  |
| 0 | Vitvi04g00262\_t001 |  |  |  |  |  |  |  |  |  |  |  |  |
| 0 | Vitvi04g00263\_t001 |  |  |  |  |  |  |  |  |  |  |  |  |
| 0 | Vitvi04g01832\_t001 |  |  |  |  |  |  |  |  |  |  |  |  |
| 0 | Vitvi04g01833\_t001 |  |  |  |  |  |  |  |  |  |  |  |  |
| 0 | Vitvi04g00264\_t001 |  |  |  |  |  |  |  |  |  |  |  |  |
| 0 | Vitvi04g00265\_t002 |  |  |  |  |  |  |  |  |  |  |  |  |
| 0 | Vitvi04g00266\_t001 |  |  |  |  |  |  |  |  |  |  |  |  |
| 0 | Vitvi04g04060\_t001 |  |  |  |  |  |  |  |  |  |  |  |  |
| 0 | Vitvi04g01834\_t001 |  |  |  |  |  |  |  |  |  |  |  |  |
| 0 | Vitvi04g00267\_t001 |  |  |  |  |  |  |  |  |  |  |  |  |
| 0 | Vitvi04g00268\_t001 |  |  |  |  |  |  |  |  |  |  |  |  |
| 0 | Vitvi04g00269\_t001 |  |  |  |  |  |  |  |  |  |  |  |  |
| 0 | Vitvi04g04061\_t001 |  |  |  |  |  |  |  |  |  |  |  |  |
| 0 | Vitvi04g00270\_t001 |  |  |  |  |  |  |  |  |  |  |  |  |
| 0 | Vitvi04g00272\_t001 |  |  |  |  |  |  |  |  |  |  |  |  |
| 0 | Vitvi04g00273\_t001 |  |  |  |  |  |  |  |  |  |  |  |  |
| 0 | Vitvi04g00275\_t001 |  |  |  |  |  |  |  |  |  |  |  |  |
| 0 | Vitvi04g00276\_t001 |  |  |  |  |  |  |  |  |  |  |  |  |
| 0 | Vitvi04g00277\_t001.1.6037826d |  |  |  |  |  |  |  |  |  |  |  |  |
| 0 | Vitvi04g00278\_t001 |  |  |  |  |  |  |  |  |  |  |  |  |
| 0 | Vitvi04g01835\_t001 |  |  |  |  |  |  |  |  |  |  |  |  |
| 0 | Vitvi04g00279\_t001 |  |  |  |  |  |  |  |  |  |  |  |  |
| 0 | Vitvi04g00280\_t001 |  |  |  |  |  |  |  |  |  |  |  |  |
| 0 | Vitvi04g00281\_t001 |  |  |  |  |  |  |  |  |  |  |  |  |
| 1 | Vitvi04g00282\_t001 |  | Vitvi11g00276\_t001 |  |  |  |  |  |  |  |  |  |  |
| 1 | Vitvi04g01836\_t001 |  | \| \| |  |  |  |  |  |  |  |  |  |  |
| 1 | Vitvi04g00283\_t001 |  | \| \| |  |  |  |  |  |  |  |  |  |  |
| 2 | Vitvi04g00284\_t001 |  | \| \| |  | Vitvi09g00308\_t001 |  |  |  |  |  |  |  |  |
| 2 | Vitvi04g00285\_t001 |  | \| \| |  | \| \| |  |  |  |  |  |  |  |  |
| 2 | Vitvi04g00286\_t001 |  | \| \| |  | \| \| |  |  |  |  |  |  |  |  |
| 2 | Vitvi04g00287\_t001 |  | \| \| |  | Vitvi09g00310\_t001 |  |  |  |  |  |  |  |  |
| 2 | Vitvi04g00288\_t001 |  | \| \| |  | \| \| |  |  |  |  |  |  |  |  |
| 2 | Vitvi04g00289\_t001 |  | \| \| |  | Vitvi09g00311\_t001 |  |  |  |  |  |  |  |  |
| 2 | Vitvi04g01837\_t001 |  | \| \| |  | \| \| |  |  |  |  |  |  |  |  |
| 2 | Vitvi04g00290\_t001 |  | \| \| |  | \| \| |  |  |  |  |  |  |  |  |
| 2 | Vitvi04g01838\_t001 |  | \| \| |  | \| \| |  |  |  |  |  |  |  |  |
| 2 | Vitvi04g00291\_t001 |  | \| \| |  | \| \| |  |  |  |  |  |  |  |  |
| 2 | Vitvi04g00292\_t001 |  | \| \| |  | \| \| |  |  |  |  |  |  |  |  |
| 2 | Vitvi04g00293\_t001 |  | \| \| |  | \| \| |  |  |  |  |  |  |  |  |
| 2 | Vitvi04g00294\_t002 |  | Vitvi11g00283\_t001 |  | \| \| |  |  |  |  |  |  |  |  |
| 2 | Vitvi04g04062\_t001 |  | \| \| |  | \| \| |  |  |  |  |  |  |  |  |
| 2 | Vitvi04g04063\_t001 |  | \| \| |  | \| \| |  |  |  |  |  |  |  |  |
| 2 | Vitvi04g00295\_t001 |  | \| \| |  | \| \| |  |  |  |  |  |  |  |  |
| 2 | Vitvi04g00297\_t001 |  | \| \| |  | \| \| |  |  |  |  |  |  |  |  |
| 2 | Vitvi04g00298\_t001 |  | \| \| |  | \| \| |  |  |  |  |  |  |  |  |
| 2 | Vitvi04g04064\_t001 |  | \| \| |  | \| \| |  |  |  |  |  |  |  |  |
| 2 | Vitvi04g00300\_t001 |  | Vitvi11g00285\_t001 |  | \| \| |  |  |  |  |  |  |  |  |
| 2 | Vitvi04g01840\_t001 |  | \| \| |  | \| \| |  |  |  |  |  |  |  |  |
| 2 | Vitvi04g01841\_t001 |  | Vitvi11g01394\_t001 |  | \| \| |  |  |  |  |  |  |  |  |
| 2 | Vitvi04g00301\_t001 |  | \| \| |  | \| \| |  |  |  |  |  |  |  |  |
| 2 | Vitvi04g00302\_t002 |  | \| \| |  | \| \| |  |  |  |  |  |  |  |  |
| 2 | Vitvi04g00303\_t001 |  | \| \| |  | \| \| |  |  |  |  |  |  |  |  |
| 2 | Vitvi04g00304\_t001 |  | \| \| |  | \| \| |  |  |  |  |  |  |  |  |
| 2 | Vitvi04g01842\_t001 |  | \| \| |  | \| \| |  |  |  |  |  |  |  |  |
| 2 | Vitvi04g04065\_t001 |  | \| \| |  | \| \| |  |  |  |  |  |  |  |  |
| 2 | Vitvi04g01843\_t001 |  | \| \| |  | \| \| |  |  |  |  |  |  |  |  |
| 2 | Vitvi04g04066\_t001 |  | \| \| |  | \| \| |  |  |  |  |  |  |  |  |
| 2 | Vitvi04g01845\_t001 |  | \| \| |  | Vitvi09g00321\_t001 |  |  |  |  |  |  |  |  |
| 2 | Vitvi04g01846\_t001 |  | \| \| |  | \| \| |  |  |  |  |  |  |  |  |
| 2 | Vitvi04g04067\_t001 |  | Vitvi11g00293\_t001 |  | \| \| |  |  |  |  |  |  |  |  |
| 2 | Vitvi04g01848\_t001 |  | \| \| |  | \| \| |  |  |  |  |  |  |  |  |
| 2 | Vitvi04g00306\_t001 |  | \| \| |  | Vitvi09g00331\_t001 |  |  |  |  |  |  |  |  |
| 2 | Vitvi04g00307\_t001 |  | \| \| |  | \| \| |  |  |  |  |  |  |  |  |
| 2 | Vitvi04g01849\_t001 |  | Vitvi11g00295\_t001 |  | \| \| |  |  |  |  |  |  |  |  |
| 2 | Vitvi04g00308\_t001 |  | \| \| |  | \| \| |  |  |  |  |  |  |  |  |
| 2 | Vitvi04g00309\_t001 |  | Vitvi11g00297\_t001 |  | \| \| |  |  |  |  |  |  |  |  |
| 2 | Vitvi04g00310\_t001 |  | \| \| |  | Vitvi09g00340\_t001 |  |  |  |  |  |  |  |  |
| 2 | Vitvi04g04068\_t001 |  | \| \| |  | \| \| |  |  |  |  |  |  |  |  |
| 2 | Vitvi04g00312\_t001 |  | Vitvi11g00304\_t001 |  | \| \| |  |  |  |  |  |  |  |  |
| 2 | Vitvi04g00313\_t001 |  | \| \| |  | \| \| |  |  |  |  |  |  |  |  |
| 2 | Vitvi04g04069\_t001 |  | \| \| |  | \| \| |  |  |  |  |  |  |  |  |
| 2 | Vitvi04g00314\_t001 |  | \| \| |  | \| \| |  |  |  |  |  |  |  |  |
| 2 | Vitvi04g00316\_t001 |  | \| \| |  | \| \| |  |  |  |  |  |  |  |  |
| 2 | Vitvi04g00317\_t001 |  | \| \| |  | \| \| |  |  |  |  |  |  |  |  |
| 2 | Vitvi04g00318\_t001 |  | \| \| |  | \| \| |  |  |  |  |  |  |  |  |
| 2 | Vitvi04g01850\_t001 |  | \| \| |  | Vitvi09g00343\_t001 |  |  |  |  |  |  |  |  |
| 2 | Vitvi04g04070\_t001 |  | \| \| |  | \| \| |  |  |  |  |  |  |  |  |
| 2 | Vitvi04g01852\_t001 |  | \| \| |  | \| \| |  |  |  |  |  |  |  |  |
| 2 | Vitvi04g04071\_t001 |  | \| \| |  | \| \| |  |  |  |  |  |  |  |  |
| 2 | Vitvi04g04072\_t001 |  | \| \| |  | \| \| |  |  |  |  |  |  |  |  |
| 2 | Vitvi04g04073\_t001 |  | \| \| |  | \| \| |  |  |  |  |  |  |  |  |
| 2 | Vitvi04g00320\_t001 |  | Vitvi11g00314\_t001 |  | \| \| |  |  |  |  |  |  |  |  |
| 2 | Vitvi04g00321\_t001 |  | \| \| |  | \| \| |  |  |  |  |  |  |  |  |
| 2 | Vitvi04g00322\_t002 |  | \| \| |  | \| \| |  |  |  |  |  |  |  |  |
| 2 | Vitvi04g00323\_t001 |  | \| \| |  | \| \| |  |  |  |  |  |  |  |  |
| 2 | Vitvi04g00324\_t001 |  | \| \| |  | \| \| |  |  |  |  |  |  |  |  |
| 2 | Vitvi04g00325\_t001 |  | \| \| |  | \| \| |  |  |  |  |  |  |  |  |
| 2 | Vitvi04g00326\_t001 |  | \| \| |  | \| \| |  |  |  |  |  |  |  |  |
| 2 | Vitvi04g00327\_t001 |  | \| \| |  | \| \| |  |  |  |  |  |  |  |  |
| 2 | Vitvi04g01854\_t001 |  | \| \| |  | \| \| |  |  |  |  |  |  |  |  |
| 2 | Vitvi04g00328\_t001 |  | Vitvi11g00318\_t001 |  | \| \| |  |  |  |  |  |  |  |  |
| 2 | Vitvi04g01855\_t001 |  | Vitvi11g00320\_t001 |  | \| \| |  |  |  |  |  |  |  |  |
| 2 | Vitvi04g04074\_t001 |  | \| \| |  | \| \| |  |  |  |  |  |  |  |  |
| 2 | Vitvi04g00331\_t001 |  | \| \| |  | \| \| |  |  |  |  |  |  |  |  |
| 2 | Vitvi04g00332\_t001 |  | \| \| |  | \| \| |  |  |  |  |  |  |  |  |
| 2 | Vitvi04g00333\_t001 |  | \| \| |  | \| \| |  |  |  |  |  |  |  |  |
| 2 | Vitvi04g00334\_t001 |  | \| \| |  | Vitvi09g00350\_t001 |  |  |  |  |  |  |  |  |
| 2 | Vitvi04g00335\_t001 |  | \| \| |  | \| \| |  |  |  |  |  |  |  |  |
| 2 | Vitvi04g01856\_t001 |  | \| \| |  | \| \| |  |  |  |  |  |  |  |  |
| 2 | Vitvi04g00336\_t001 |  | Vitvi11g00328\_t001 |  | \| \| |  |  |  |  |  |  |  |  |
| 2 | Vitvi04g00337\_t001 |  | Vitvi11g00329\_t001 |  | \| \| |  |  |  |  |  |  |  |  |
| 2 | Vitvi04g01857\_t001 |  | \| \| |  | \| \| |  |  |  |  |  |  |  |  |
| 2 | Vitvi04g00338\_t001 |  | \| \| |  | \| \| |  |  |  |  |  |  |  |  |
| 2 | Vitvi04g00339\_t001 |  | \| \| |  | \| \| |  |  |  |  |  |  |  |  |
| 2 | Vitvi04g01858\_t001 |  | \| \| |  | Vitvi09g00370\_t001 |  |  |  |  |  |  |  |  |
| 1 | Vitvi04g00340\_t001 |  | Vitvi11g00338\_t001 |  |  |  |  |  |  |  |  |  |  |
| 1 | Vitvi04g00341\_t001 |  | \| \| |  |  |  |  |  |  |  |  |  |  |
| 1 | Vitvi04g04075\_t001 |  | \| \| |  |  |  |  |  |  |  |  |  |  |
| 1 | Vitvi04g00342\_t001 |  | \| \| |  |  |  |  |  |  |  |  |  |  |
| 1 | Vitvi04g04076\_t001 |  | \| \| |  |  |  |  |  |  |  |  |  |  |
| 1 | Vitvi04g00343\_t001 |  | \| \| |  |  |  |  |  |  |  |  |  |  |
| 1 | Vitvi04g04077\_t001 |  | \| \| |  |  |  |  |  |  |  |  |  |  |
| 1 | Vitvi04g00344\_t001 |  | \| \| |  |  |  |  |  |  |  |  |  |  |
| 1 | Vitvi04g00345\_t001 |  | \| \| |  |  |  |  |  |  |  |  |  |  |
| 1 | Vitvi04g04078\_t001 |  | \| \| |  |  |  |  |  |  |  |  |  |  |
| 1 | Vitvi04g00346\_t001 |  | \| \| |  |  |  |  |  |  |  |  |  |  |
| 1 | Vitvi04g00347\_t001 |  | \| \| |  |  |  |  |  |  |  |  |  |  |
| 1 | Vitvi04g04079\_t001 |  | \| \| |  |  |  |  |  |  |  |  |  |  |
| 1 | Vitvi04g04080\_t001 |  | \| \| |  |  |  |  |  |  |  |  |  |  |
| 1 | Vitvi04g04081\_t001 |  | \| \| |  |  |  |  |  |  |  |  |  |  |
| 1 | Vitvi04g00350\_t001 |  | \| \| |  |  |  |  |  |  |  |  |  |  |
| 1 | Vitvi04g00352\_t001 |  | \| \| |  |  |  |  |  |  |  |  |  |  |
| 1 | Vitvi04g00354\_t001 |  | \| \| |  |  |  |  |  |  |  |  |  |  |
| 1 | Vitvi04g04082\_t001 |  | \| \| |  |  |  |  |  |  |  |  |  |  |
| 1 | Vitvi04g04083\_t001 |  | \| \| |  |  |  |  |  |  |  |  |  |  |
| 1 | Vitvi04g01866\_t001 |  | \| \| |  |  |  |  |  |  |  |  |  |  |
| 1 | Vitvi04g04084\_t001 |  | \| \| |  |  |  |  |  |  |  |  |  |  |
| 1 | Vitvi04g04085\_t001 |  | \| \| |  |  |  |  |  |  |  |  |  |  |
| 1 | Vitvi04g01870\_t001 |  | \| \| |  |  |  |  |  |  |  |  |  |  |
| 1 | Vitvi04g04086\_t001 |  | \| \| |  |  |  |  |  |  |  |  |  |  |
| 1 | Vitvi04g01873\_t006 |  | Vitvi11g00340\_t001 |  |  |  |  |  |  |  |  |  |  |
| 1 | Vitvi04g00357\_t001 |  | \| \| |  |  |  |  |  |  |  |  |  |  |
| 1 | Vitvi04g04087\_t001 |  | \| \| |  |  |  |  |  |  |  |  |  |  |
| 1 | Vitvi04g00359\_t001 |  | \| \| |  |  |  |  |  |  |  |  |  |  |
| 1 | Vitvi04g00360\_t001 |  | \| \| |  |  |  |  |  |  |  |  |  |  |
| 1 | Vitvi04g00361\_t001 |  | \| \| |  |  |  |  |  |  |  |  |  |  |
| 1 | Vitvi04g04088\_t001 |  | Vitvi11g01409\_t001 |  |  |  |  |  |  |  |  |  |  |
| 1 | Vitvi04g04089\_t001 |  | \| \| |  |  |  |  |  |  |  |  |  |  |
| 1 | Vitvi04g04090\_t002 |  | \| \| |  |  |  |  |  |  |  |  |  |  |
| 1 | Vitvi04g04091\_t001 |  | \| \| |  |  |  |  |  |  |  |  |  |  |
| 1 | Vitvi04g04092\_t001 |  | \| \| |  |  |  |  |  |  |  |  |  |  |
| 2 | Vitvi04g00365\_t001 |  | \| \| |  | Vitvi11g00398\_t001 |  |  |  |  |  |  |  |  |
| 2 | Vitvi04g00366\_t001 |  | \| \| |  | \| \| |  |  |  |  |  |  |  |  |
| 2 | Vitvi04g00367\_t001 |  | \| \| |  | \| \| |  |  |  |  |  |  |  |  |
| 2 | Vitvi04g04093\_t001 |  | \| \| |  | \| \| |  |  |  |  |  |  |  |  |
| 2 | Vitvi04g00369\_t001 |  | \| \| |  | \| \| |  |  |  |  |  |  |  |  |
| 2 | Vitvi04g00371\_t001 |  | \| \| |  | Vitvi11g00395\_t002 |  |  |  |  |  |  |  |  |
| 2 | Vitvi04g00372\_t001 |  | \| \| |  | Vitvi11g00390\_t001 |  |  |  |  |  |  |  |  |
| 2 | Vitvi04g04094\_t001 |  | \| \| |  | \| \| |  |  |  |  |  |  |  |  |
| 2 | Vitvi04g00373\_t001 |  | \| \| |  | \| \| |  |  |  |  |  |  |  |  |
| 2 | Vitvi04g00374\_t001 |  | \| \| |  | \| \| |  |  |  |  |  |  |  |  |
| 2 | Vitvi04g04095\_t001 |  | \| \| |  | \| \| |  |  |  |  |  |  |  |  |
| 2 | Vitvi04g00375\_t001 |  | \| \| |  | \| \| |  |  |  |  |  |  |  |  |
| 2 | Vitvi04g00376\_t001 |  | \| \| |  | \| \| |  |  |  |  |  |  |  |  |
| 2 | Vitvi04g00377\_t001 |  | \| \| |  | \| \| |  |  |  |  |  |  |  |  |
| 2 | Vitvi04g01874\_t001 |  | \| \| |  | Vitvi11g00385\_t001 |  |  |  |  |  |  |  |  |
| 2 | Vitvi04g00378\_t001 |  | \| \| |  | Vitvi11g00381\_t001 |  |  |  |  |  |  |  |  |
| 2 | Vitvi04g00380\_t001 |  | \| \| |  | \| \| |  |  |  |  |  |  |  |  |
| 2 | Vitvi04g00381\_t002 |  | Vitvi11g00367\_t001 |  | \| \| |  |  |  |  |  |  |  |  |
| 1 | Vitvi04g04096\_t001 |  |  |  | \| \| |  |  |  |  |  |  |  |  |
| 1 | Vitvi04g04097\_t001 |  |  |  | \| \| |  |  |  |  |  |  |  |  |
| 1 | Vitvi04g00383\_t001 |  |  |  | \| \| |  |  |  |  |  |  |  |  |
| 1 | Vitvi04g00384\_t001 |  |  |  | Vitvi11g00363\_t001 |  |  |  |  |  |  |  |  |
| 0 | Vitvi04g00385\_t001 |  |  |  |  |  |  |  |  |  |  |  |  |
| 0 | Vitvi04g00386\_t001 |  |  |  |  |  |  |  |  |  |  |  |  |
| 0 | Vitvi04g00387\_t001 |  |  |  |  |  |  |  |  |  |  |  |  |
| 0 | Vitvi04g00388\_t003 |  |  |  |  |  |  |  |  |  |  |  |  |
| 0 | Vitvi04g00390\_t003 |  |  |  |  |  |  |  |  |  |  |  |  |
| 0 | Vitvi04g01877\_t001 |  |  |  |  |  |  |  |  |  |  |  |  |
| 0 | Vitvi04g00391\_t001 |  |  |  |  |  |  |  |  |  |  |  |  |
| 0 | Vitvi04g00392\_t001 |  |  |  |  |  |  |  |  |  |  |  |  |
| 0 | Vitvi04g00394\_t001 |  |  |  |  |  |  |  |  |  |  |  |  |
| 0 | Vitvi04g01878\_t001 |  |  |  |  |  |  |  |  |  |  |  |  |
| 0 | Vitvi04g01880\_t001 |  |  |  |  |  |  |  |  |  |  |  |  |
| 0 | Vitvi04g00397\_t001 |  |  |  |  |  |  |  |  |  |  |  |  |
| 0 | Vitvi04g04098\_t001 |  |  |  |  |  |  |  |  |  |  |  |  |
| 0 | Vitvi04g00399\_t001 |  |  |  |  |  |  |  |  |  |  |  |  |
| 0 | Vitvi04g01882\_t001 |  |  |  |  |  |  |  |  |  |  |  |  |
| 0 | Vitvi04g00400\_t001 |  |  |  |  |  |  |  |  |  |  |  |  |
| 0 | Vitvi04g00401\_t001 |  |  |  |  |  |  |  |  |  |  |  |  |
| 0 | Vitvi04g01883\_t001 |  |  |  |  |  |  |  |  |  |  |  |  |
| 0 | Vitvi04g01884\_t001 |  |  |  |  |  |  |  |  |  |  |  |  |
| 0 | Vitvi04g04099\_t001 |  |  |  |  |  |  |  |  |  |  |  |  |
| 0 | Vitvi04g01885\_t001 |  |  |  |  |  |  |  |  |  |  |  |  |
| 0 | Vitvi04g04100\_t001 |  |  |  |  |  |  |  |  |  |  |  |  |
| 0 | Vitvi04g04101\_t001 |  |  |  |  |  |  |  |  |  |  |  |  |
| 0 | Vitvi04g00405\_t001 |  |  |  |  |  |  |  |  |  |  |  |  |
| 0 | Vitvi04g00406\_t001 |  |  |  |  |  |  |  |  |  |  |  |  |
| 0 | Vitvi04g04102\_t001 |  |  |  |  |  |  |  |  |  |  |  |  |
| 1 | Vitvi04g00408\_t001 |  | Vitvi09g00499\_t001 |  |  |  |  |  |  |  |  |  |  |
| 1 | Vitvi04g00409\_t001 |  | Vitvi09g04160\_t001 |  |  |  |  |  |  |  |  |  |  |
| 1 | Vitvi04g00410\_t001 |  | \| \| |  |  |  |  |  |  |  |  |  |  |
| 1 | Vitvi04g04103\_t001 |  | \| \| |  |  |  |  |  |  |  |  |  |  |
| 1 | Vitvi04g00411\_t001 |  | \| \| |  |  |  |  |  |  |  |  |  |  |
| 1 | Vitvi04g00412\_t001 |  | \| \| |  |  |  |  |  |  |  |  |  |  |
| 1 | Vitvi04g00414\_t001 |  | \| \| |  |  |  |  |  |  |  |  |  |  |
| 1 | Vitvi04g00415\_t001 |  | \| \| |  |  |  |  |  |  |  |  |  |  |
| 1 | Vitvi04g00416\_t001 |  | Vitvi09g00490\_t001 |  |  |  |  |  |  |  |  |  |  |
| 1 | Vitvi04g01887\_t001 |  | \| \| |  |  |  |  |  |  |  |  |  |  |
| 1 | Vitvi04g00418\_t001 |  | \| \| |  |  |  |  |  |  |  |  |  |  |
| 1 | Vitvi04g00419\_t001 |  | \| \| |  |  |  |  |  |  |  |  |  |  |
| 1 | Vitvi04g04104\_t001 |  | \| \| |  |  |  |  |  |  |  |  |  |  |
| 3 | Vitvi04g00421\_t001 |  | \| \| |  | Vitvi09g04147\_t001 |  | Vitvi11g00398\_t001 |  |  |  |  |  |  |
| 3 | Vitvi04g00422\_t001 |  | \| \| |  | \| \| |  | \| \| |  |  |  |  |  |  |
| 3 | Vitvi04g04105\_t001 |  | \| \| |  | \| \| |  | \| \| |  |  |  |  |  |  |
| 3 | Vitvi04g04106\_t001 |  | \| \| |  | \| \| |  | \| \| |  |  |  |  |  |  |
| 3 | Vitvi04g01888\_t001 |  | \| \| |  | \| \| |  | \| \| |  |  |  |  |  |  |
| 3 | Vitvi04g00423\_t001 |  | Vitvi09g00462\_t001 |  | Vitvi09g00462\_t001 |  | \| \| |  |  |  |  |  |  |
| 3 | Vitvi04g04107\_t001 |  | \| \| |  | \| \| |  | \| \| |  |  |  |  |  |  |
| 3 | Vitvi04g00425\_t001 |  | \| \| |  | \| \| |  | \| \| |  |  |  |  |  |  |
| 3 | Vitvi04g04108\_t001 |  | Vitvi09g04148\_t001 |  | Vitvi09g04152\_t001 |  | \| \| |  |  |  |  |  |  |
| 3 | Vitvi04g04109\_t001 |  | \| \| |  | \| \| |  | \| \| |  |  |  |  |  |  |
| 3 | Vitvi04g01889\_t001 |  | \| \| |  | \| \| |  | \| \| |  |  |  |  |  |  |
| 3 | Vitvi04g00430\_t001 |  | \| \| |  | \| \| |  | \| \| |  |  |  |  |  |  |
| 3 | Vitvi04g04110\_t001 |  | \| \| |  | \| \| |  | \| \| |  |  |  |  |  |  |
| 3 | Vitvi04g04111\_t001 |  | \| \| |  | \| \| |  | \| \| |  |  |  |  |  |  |
| 3 | Vitvi04g00431\_t003 |  | \| \| |  | \| \| |  | Vitvi11g00399\_t001 |  |  |  |  |  |  |
| 3 | Vitvi04g00433\_t001 |  | Vitvi09g00454\_t001 |  | \| \| |  | \| \| |  |  |  |  |  |  |
| 3 | Vitvi04g00434\_t001 |  | \| \| |  | \| \| |  | \| \| |  |  |  |  |  |  |
| 3 | Vitvi04g00435\_t001 |  | Vitvi09g00452\_t001 |  | \| \| |  | \| \| |  |  |  |  |  |  |
| 2 | Vitvi04g00436\_t001 |  |  |  | \| \| |  | \| \| |  |  |  |  |  |  |
| 2 | Vitvi04g00437\_t001 |  |  |  | \| \| |  | \| \| |  |  |  |  |  |  |
| 2 | Vitvi04g00438\_t001 |  |  |  | \| \| |  | \| \| |  |  |  |  |  |  |
| 2 | Vitvi04g00439\_t001 |  |  |  | Vitvi09g00500\_t001 |  | Vitvi11g00419\_t001 |  |  |  |  |  |  |
| 2 | Vitvi04g00440\_t001 |  |  |  | \| \| |  | Vitvi11g00420\_t001 |  |  |  |  |  |  |
| 2 | Vitvi04g00441\_t001 |  |  |  | \| \| |  | \| \| |  |  |  |  |  |  |
| 2 | Vitvi04g00442\_t001 |  |  |  | Vitvi09g00512\_t001 |  | \| \| |  |  |  |  |  |  |
| 2 | Vitvi04g04112\_t001 |  |  |  | Vitvi09g00513\_t001 |  | Vitvi11g04093\_t001 |  |  |  |  |  |  |
| 2 | Vitvi04g01893\_t001 |  |  |  | \| \| |  | \| \| |  |  |  |  |  |  |
| 2 | Vitvi04g04113\_t001 |  |  |  | \| \| |  | \| \| |  |  |  |  |  |  |
| 2 | Vitvi04g00444\_t001 |  |  |  | \| \| |  | Vitvi11g00425\_t003 |  |  |  |  |  |  |
| 2 | Vitvi04g04114\_t001 |  |  |  | \| \| |  | \| \| |  |  |  |  |  |  |
| 2 | Vitvi04g00445\_t001 |  |  |  | \| \| |  | \| \| |  |  |  |  |  |  |
| 2 | Vitvi04g00446\_t001 |  |  |  | \| \| |  | \| \| |  |  |  |  |  |  |
| 2 | Vitvi04g00447\_t001 |  |  |  | \| \| |  | \| \| |  |  |  |  |  |  |
| 2 | Vitvi04g00449\_t001 |  |  |  | \| \| |  | \| \| |  |  |  |  |  |  |
| 2 | Vitvi04g00450\_t001 |  |  |  | \| \| |  | \| \| |  |  |  |  |  |  |
| 2 | Vitvi04g00451\_t001 |  |  |  | Vitvi09g01645\_t001 |  | \| \| |  |  |  |  |  |  |
| 2 | Vitvi04g00452\_t001 |  |  |  | \| \| |  | \| \| |  |  |  |  |  |  |
| 2 | Vitvi04g00454\_t001 |  |  |  | Vitvi09g00518\_t001 |  | Vitvi11g00428\_t001 |  |  |  |  |  |  |
| 2 | Vitvi04g04115\_t001 |  |  |  | \| \| |  | \| \| |  |  |  |  |  |  |
| 2 | Vitvi04g00455\_t001 |  |  |  | Vitvi09g00519\_t001 |  | \| \| |  |  |  |  |  |  |
| 2 | Vitvi04g00459\_t001 |  |  |  | \| \| |  | Vitvi11g00433\_t001 |  |  |  |  |  |  |
| 2 | Vitvi04g01894\_t001 |  |  |  | Vitvi09g04170\_t001 |  | Vitvi11g04099\_t001 |  |  |  |  |  |  |
| 2 | Vitvi04g01895\_t001 |  |  |  | \| \| |  | \| \| |  |  |  |  |  |  |
| 2 | Vitvi04g04116\_t001 |  |  |  | \| \| |  | \| \| |  |  |  |  |  |  |
| 2 | Vitvi04g04117\_t001 |  |  |  | \| \| |  | \| \| |  |  |  |  |  |  |
| 2 | Vitvi04g00461\_t001 |  |  |  | \| \| |  | \| \| |  |  |  |  |  |  |
| 2 | Vitvi04g04118\_t001 |  |  |  | \| \| |  | \| \| |  |  |  |  |  |  |
| 2 | Vitvi04g04119\_t001 |  |  |  | \| \| |  | \| \| |  |  |  |  |  |  |
| 2 | Vitvi04g01897\_t001 |  |  |  | Vitvi09g00530\_t001 |  | \| \| |  |  |  |  |  |  |
| 2 | Vitvi04g00462\_t001 |  |  |  | Vitvi09g00550\_t001 |  | \| \| |  |  |  |  |  |  |
| 2 | Vitvi04g00463\_t001 |  |  |  | \| \| |  | \| \| |  |  |  |  |  |  |
| 2 | Vitvi04g01898\_t001 |  |  |  | \| \| |  | Vitvi11g01429\_t001 |  |  |  |  |  |  |
| 2 | Vitvi04g00464\_t001 |  |  |  | \| \| |  | \| \| |  |  |  |  |  |  |
| 2 | Vitvi04g00465\_t001 |  |  |  | \| \| |  | \| \| |  |  |  |  |  |  |
| 2 | Vitvi04g01899\_t001 |  |  |  | Vitvi09g04178\_t001 |  | Vitvi11g00450\_t001 |  |  |  |  |  |  |
| 2 | Vitvi04g00466\_t001 |  |  |  | \| \| |  | Vitvi11g00451\_t001 |  |  |  |  |  |  |
| 2 | Vitvi04g04120\_t001 |  |  |  | \| \| |  | \| \| |  |  |  |  |  |  |
| 2 | Vitvi04g00467\_t001 |  |  |  | Vitvi09g00573\_t001 |  | Vitvi11g04102\_t001 |  |  |  |  |  |  |
| 2 | Vitvi04g00468\_t001 |  |  |  | \| \| |  | \| \| |  |  |  |  |  |  |
| 2 | Vitvi04g00469\_t002 |  |  |  | \| \| |  | \| \| |  |  |  |  |  |  |
| 2 | Vitvi04g01900\_t001 |  |  |  | \| \| |  | \| \| |  |  |  |  |  |  |
| 2 | Vitvi04g04121\_t001 |  |  |  | \| \| |  | Vitvi11g00454\_t002 |  |  |  |  |  |  |
| 2 | Vitvi04g00471\_t001 |  |  |  | \| \| |  | Vitvi11g01435\_t001 |  |  |  |  |  |  |
| 2 | Vitvi04g00472\_t001 |  |  |  | \| \| |  | \| \| |  |  |  |  |  |  |
| 2 | Vitvi04g01901\_t001 |  |  |  | \| \| |  | \| \| |  |  |  |  |  |  |
| 2 | Vitvi04g00473\_t001 |  |  |  | \| \| |  | \| \| |  |  |  |  |  |  |
| 2 | Vitvi04g00474\_t001 |  |  |  | Vitvi09g00588\_t001 |  | \| \| |  |  |  |  |  |  |
| 2 | Vitvi04g01902\_t001 |  |  |  | \| \| |  | Vitvi11g04105\_t003 |  |  |  |  |  |  |
| 2 | Vitvi04g01903\_t001 |  |  |  | \| \| |  | \| \| |  |  |  |  |  |  |
| 2 | Vitvi04g04122\_t001 |  |  |  | \| \| |  | \| \| |  |  |  |  |  |  |
| 2 | Vitvi04g01904\_t001 |  |  |  | \| \| |  | \| \| |  |  |  |  |  |  |
| 2 | Vitvi04g01905\_t001 |  |  |  | \| \| |  | \| \| |  |  |  |  |  |  |
| 2 | Vitvi04g01906\_t001 |  |  |  | \| \| |  | \| \| |  |  |  |  |  |  |
| 2 | Vitvi04g01907\_t001 |  |  |  | \| \| |  | \| \| |  |  |  |  |  |  |
| 2 | Vitvi04g01908\_t001 |  |  |  | \| \| |  | \| \| |  |  |  |  |  |  |
| 2 | Vitvi04g01909\_t001 |  |  |  | \| \| |  | \| \| |  |  |  |  |  |  |
| 2 | Vitvi04g00476\_t001 |  |  |  | Vitvi09g00591\_t001 |  | Vitvi11g00466\_t001 |  |  |  |  |  |  |
| 2 | Vitvi04g04123\_t001 |  |  |  | \| \| |  | \| \| |  |  |  |  |  |  |
| 2 | Vitvi04g04124\_t001 |  |  |  | \| \| |  | \| \| |  |  |  |  |  |  |
| 2 | Vitvi04g00477\_t001 |  |  |  | \| \| |  | \| \| |  |  |  |  |  |  |
| 2 | Vitvi04g00479\_t001 |  |  |  | Vitvi09g00593\_t001 |  | Vitvi11g00467\_t001 |  |  |  |  |  |  |
| 2 | Vitvi04g00480\_t001 |  |  |  | Vitvi09g00595\_t001 |  | Vitvi11g00469\_t001 |  |  |  |  |  |  |
| 2 | Vitvi04g00481\_t003 |  |  |  | Vitvi09g04185\_t001 |  | Vitvi11g00474\_t001 |  |  |  |  |  |  |
| 2 | Vitvi04g00482\_t001 |  |  |  | \| \| |  | Vitvi11g00475\_t001 |  |  |  |  |  |  |
| 2 | Vitvi04g00483\_t002 |  |  |  | \| \| |  | Vitvi11g00480\_t001 |  |  |  |  |  |  |
| 2 | Vitvi04g00484\_t001 |  |  |  | \| \| |  | \| \| |  |  |  |  |  |  |
| 2 | Vitvi04g00485\_t001 |  |  |  | Vitvi09g00604\_t001 |  | \| \| |  |  |  |  |  |  |
| 2 | Vitvi04g00486\_t001 |  |  |  | \| \| |  | \| \| |  |  |  |  |  |  |
| 2 | Vitvi04g00487\_t001 |  |  |  | \| \| |  | Vitvi11g00484\_t001 |  |  |  |  |  |  |
| 2 | Vitvi04g00489\_t001 |  |  |  | \| \| |  | \| \| |  |  |  |  |  |  |
| 2 | Vitvi04g00490\_t001 |  |  |  | \| \| |  | \| \| |  |  |  |  |  |  |
| 2 | Vitvi04g00491\_t001 |  |  |  | \| \| |  | \| \| |  |  |  |  |  |  |
| 2 | Vitvi04g00493\_t001 |  |  |  | \| \| |  | Vitvi11g00497\_t001 |  |  |  |  |  |  |
| 2 | Vitvi04g04125\_t001 |  |  |  | \| \| |  | \| \| |  |  |  |  |  |  |
| 2 | Vitvi04g00494\_t001 |  |  |  | \| \| |  | Vitvi11g00505\_t001 |  |  |  |  |  |  |
| 2 | Vitvi04g00495\_t001 |  |  |  | Vitvi09g00625\_t001 |  | Vitvi11g01447\_t001 |  |  |  |  |  |  |
| 2 | Vitvi04g00496\_t001 |  |  |  | \| \| |  | \| \| |  |  |  |  |  |  |
| 2 | Vitvi04g00497\_t001 |  |  |  | Vitvi09g00627\_t001 |  | \| \| |  |  |  |  |  |  |
| 2 | Vitvi04g00498\_t001 |  |  |  | \| \| |  | \| \| |  |  |  |  |  |  |
| 2 | Vitvi04g04126\_t001 |  |  |  | \| \| |  | \| \| |  |  |  |  |  |  |
| 2 | Vitvi04g00499\_t001 |  |  |  | Vitvi09g00629\_t002 |  | \| \| |  |  |  |  |  |  |
| 2 | Vitvi04g00500\_t001 |  |  |  | Vitvi09g00644\_t001 |  | \| \| |  |  |  |  |  |  |
| 2 | Vitvi04g00501\_t001 |  |  |  | \| \| |  | Vitvi11g00518\_t001 |  |  |  |  |  |  |
| 2 | Vitvi04g00503\_t001 |  |  |  | \| \| |  | Vitvi11g04113\_t001 |  |  |  |  |  |  |
| 2 | Vitvi04g00504\_t001 |  |  |  | Vitvi09g00649\_t001 |  | \| \| |  |  |  |  |  |  |
| 1 | Vitvi04g04127\_t001 |  |  |  |  |  | \| \| |  |  |  |  |  |  |
| 1 | Vitvi04g00505\_t001 |  |  |  |  |  | Vitvi11g00521\_t001 |  |  |  |  |  |  |
| 1 | Vitvi04g00506\_t001 |  |  |  |  |  | \| \| |  |  |  |  |  |  |
| 1 | Vitvi04g00507\_t001 |  |  |  |  |  | Vitvi11g00528\_t001 |  |  |  |  |  |  |
| 1 | Vitvi04g01910\_t001 |  |  |  |  |  | Vitvi11g00538\_t001 |  |  |  |  |  |  |
| 1 | Vitvi04g04128\_t001 |  |  |  |  |  | \| \| |  |  |  |  |  |  |
| 1 | Vitvi04g00508\_t001 |  |  |  |  |  | Vitvi11g00542\_t001 |  |  |  |  |  |  |
| 1 | Vitvi04g00509\_t001 |  |  |  |  |  | Vitvi11g00547\_t001 |  |  |  |  |  |  |
| 1 | Vitvi04g00510\_t001 |  |  |  |  |  | \| \| |  |  |  |  |  |  |
| 1 | Vitvi04g00511\_t001 |  |  |  |  |  | \| \| |  |  |  |  |  |  |
| 1 | Vitvi04g00512\_t001 |  |  |  |  |  | \| \| |  |  |  |  |  |  |
| 1 | Vitvi04g00513\_t001 |  |  |  |  |  | \| \| |  |  |  |  |  |  |
| 1 | Vitvi04g00514\_t001 |  |  |  |  |  | \| \| |  |  |  |  |  |  |
| 1 | Vitvi04g00515\_t001 |  |  |  |  |  | \| \| |  |  |  |  |  |  |
| 1 | Vitvi04g04129\_t001 |  |  |  |  |  | \| \| |  |  |  |  |  |  |
| 1 | Vitvi04g04130\_t001 |  |  |  |  |  | \| \| |  |  |  |  |  |  |
| 1 | Vitvi04g00517\_t001 |  |  |  |  |  | \| \| |  |  |  |  |  |  |
| 1 | Vitvi04g00519\_t001 |  |  |  |  |  | \| \| |  |  |  |  |  |  |
| 1 | Vitvi04g00520\_t001 |  |  |  |  |  | \| \| |  |  |  |  |  |  |
| 1 | Vitvi04g00521\_t001 |  |  |  |  |  | \| \| |  |  |  |  |  |  |
| 1 | Vitvi04g00522\_t001 |  |  |  |  |  | \| \| |  |  |  |  |  |  |
| 1 | Vitvi04g00523\_t001 |  |  |  |  |  | \| \| |  |  |  |  |  |  |
| 1 | Vitvi04g00524\_t001 |  |  |  |  |  | \| \| |  |  |  |  |  |  |
| 1 | Vitvi04g00525\_t001 |  |  |  |  |  | \| \| |  |  |  |  |  |  |
| 1 | Vitvi04g00526\_t001 |  |  |  |  |  | \| \| |  |  |  |  |  |  |
| 2 | Vitvi04g00527\_t001 |  | Vitvi09g00827\_t001 |  |  |  | \| \| |  |  |  |  |  |  |
| 2 | Vitvi04g00528\_t002 |  | Vitvi09g00826\_t001 |  |  |  | \| \| |  |  |  |  |  |  |
| 2 | Vitvi04g00530\_t002 |  | Vitvi09g00817\_t001 |  |  |  | \| \| |  |  |  |  |  |  |
| 2 | Vitvi04g00531\_t001 |  | \| \| |  |  |  | \| \| |  |  |  |  |  |  |
| 2 | Vitvi04g00532\_t001 |  | \| \| |  |  |  | Vitvi11g00548\_t001 |  |  |  |  |  |  |
| 1 | Vitvi04g01914\_t001 |  | \| \| |  |  |  |  |  |  |  |  |  |  |
| 1 | Vitvi04g00533\_t001 |  | \| \| |  |  |  |  |  |  |  |  |  |  |
| 1 | Vitvi04g00535\_t001 |  | \| \| |  |  |  |  |  |  |  |  |  |  |
| 1 | Vitvi04g00536\_t001 |  | \| \| |  |  |  |  |  |  |  |  |  |  |
| 1 | Vitvi04g00537\_t001 |  | \| \| |  |  |  |  |  |  |  |  |  |  |
| 1 | Vitvi04g00538\_t001 |  | \| \| |  |  |  |  |  |  |  |  |  |  |
| 1 | Vitvi04g04131\_t001 |  | \| \| |  |  |  |  |  |  |  |  |  |  |
| 1 | Vitvi04g01916\_t001 |  | \| \| |  |  |  |  |  |  |  |  |  |  |
| 1 | Vitvi04g04132\_t001 |  | \| \| |  |  |  |  |  |  |  |  |  |  |
| 1 | Vitvi04g00540\_t001 |  | Vitvi09g00805\_t001 |  |  |  |  |  |  |  |  |  |  |
| 1 | Vitvi04g00541\_t001 |  | \| \| |  |  |  |  |  |  |  |  |  |  |
| 1 | Vitvi04g00543\_t003 |  | \| \| |  |  |  |  |  |  |  |  |  |  |
| 1 | Vitvi04g00545\_t001 |  | \| \| |  |  |  |  |  |  |  |  |  |  |
| 1 | Vitvi04g04133\_t001 |  | \| \| |  |  |  |  |  |  |  |  |  |  |
| 2 | Vitvi04g00546\_t001 |  | \| \| |  | Vitvi11g00601\_t002 |  |  |  |  |  |  |  |  |
| 2 | Vitvi04g01917\_t001 |  | \| \| |  | \| \| |  |  |  |  |  |  |  |  |
| 2 | Vitvi04g01918\_t001 |  | \| \| |  | Vitvi11g01474\_t001 |  |  |  |  |  |  |  |  |
| 2 | Vitvi04g01919\_t001 |  | \| \| |  | \| \| |  |  |  |  |  |  |  |  |
| 2 | Vitvi04g01920\_t001 |  | \| \| |  | \| \| |  |  |  |  |  |  |  |  |
| 2 | Vitvi04g00548\_t002 |  | \| \| |  | \| \| |  |  |  |  |  |  |  |  |
| 2 | Vitvi04g04134\_t001 |  | \| \| |  | \| \| |  |  |  |  |  |  |  |  |
| 2 | Vitvi04g00549\_t001 |  | \| \| |  | \| \| |  |  |  |  |  |  |  |  |
| 2 | Vitvi04g00550\_t001 |  | \| \| |  | \| \| |  |  |  |  |  |  |  |  |
| 2 | Vitvi04g00552\_t001 |  | Vitvi09g00791\_t001 |  | \| \| |  |  |  |  |  |  |  |  |
| 2 | Vitvi04g00554\_t001 |  | \| \| |  | Vitvi11g00590\_t001 |  |  |  |  |  |  |  |  |
| 2 | Vitvi04g00555\_t001 |  | \| \| |  | Vitvi11g00588\_t001 |  |  |  |  |  |  |  |  |
| 2 | Vitvi04g00556\_t001 |  | Vitvi09g00773\_t001 |  | Vitvi11g00584\_t001 |  |  |  |  |  |  |  |  |
| 2 | Vitvi04g00557\_t001 |  | Vitvi09g00772\_t001 |  | Vitvi11g00582\_t001 |  |  |  |  |  |  |  |  |
| 2 | Vitvi04g04135\_t001 |  | \| \| |  | \| \| |  |  |  |  |  |  |  |  |
| 2 | Vitvi04g00558\_t002 |  | \| \| |  | \| \| |  |  |  |  |  |  |  |  |
| 2 | Vitvi04g00559\_t001 |  | \| \| |  | Vitvi11g00579\_t001 |  |  |  |  |  |  |  |  |
| 2 | Vitvi04g00564\_t001 |  | \| \| |  | \| \| |  |  |  |  |  |  |  |  |
| 2 | Vitvi04g00565\_t001 |  | \| \| |  | Vitvi11g00575\_t001 |  |  |  |  |  |  |  |  |
| 2 | Vitvi04g04136\_t002 |  | \| \| |  | \| \| |  |  |  |  |  |  |  |  |
| 2 | Vitvi04g00567\_t001 |  | Vitvi09g00753\_t002 |  | \| \| |  |  |  |  |  |  |  |  |
| 1 | Vitvi04g04137\_t001 |  |  |  | \| \| |  |  |  |  |  |  |  |  |
| 1 | Vitvi04g00568\_t001 |  |  |  | \| \| |  |  |  |  |  |  |  |  |
| 1 | Vitvi04g00570\_t001 |  |  |  | \| \| |  |  |  |  |  |  |  |  |
| 1 | Vitvi04g01922\_t001 |  |  |  | \| \| |  |  |  |  |  |  |  |  |
| 1 | Vitvi04g00571\_t001 |  |  |  | \| \| |  |  |  |  |  |  |  |  |
| 1 | Vitvi04g00572\_t001 |  |  |  | \| \| |  |  |  |  |  |  |  |  |
| 1 | Vitvi04g00573\_t001 |  |  |  | \| \| |  |  |  |  |  |  |  |  |
| 1 | Vitvi04g01923\_t001 |  |  |  | \| \| |  |  |  |  |  |  |  |  |
| 1 | Vitvi04g04138\_t001 |  |  |  | \| \| |  |  |  |  |  |  |  |  |
| 1 | Vitvi04g04139\_t001 |  |  |  | \| \| |  |  |  |  |  |  |  |  |
| 1 | Vitvi04g01925\_t001 |  |  |  | Vitvi11g01463\_t001 |  |  |  |  |  |  |  |  |
| 1 | Vitvi04g01926\_t001 |  |  |  | \| \| |  |  |  |  |  |  |  |  |
| 1 | Vitvi04g00580\_t001 |  |  |  | \| \| |  |  |  |  |  |  |  |  |
| 1 | Vitvi04g00581\_t001 |  |  |  | \| \| |  |  |  |  |  |  |  |  |
| 1 | Vitvi04g00582\_t001 |  |  |  | \| \| |  |  |  |  |  |  |  |  |
| 1 | Vitvi04g00583\_t001 |  |  |  | \| \| |  |  |  |  |  |  |  |  |
| 1 | Vitvi04g00584\_t001 |  |  |  | Vitvi11g00549\_t001 |  |  |  |  |  |  |  |  |
| 1 | Vitvi04g00585\_t001 |  |  |  | Vitvi11g00548\_t001 |  |  |  |  |  |  |  |  |
| 1 | Vitvi04g00586\_t001 |  | Vitvi11g00673\_t001 |  |  |  |  |  |  |  |  |  |  |
| 1 | Vitvi04g00590\_t001 |  | \| \| |  |  |  |  |  |  |  |  |  |  |
| 1 | Vitvi04g00591\_t001 |  | \| \| |  |  |  |  |  |  |  |  |  |  |
| 1 | Vitvi04g00592\_t001 |  | \| \| |  |  |  |  |  |  |  |  |  |  |
| 1 | Vitvi04g00594\_t001 |  | \| \| |  |  |  |  |  |  |  |  |  |  |
| 1 | Vitvi04g04140\_t001 |  | \| \| |  |  |  |  |  |  |  |  |  |  |
| 1 | Vitvi04g01927\_t001 |  | \| \| |  |  |  |  |  |  |  |  |  |  |
| 1 | Vitvi04g04141\_t001 |  | \| \| |  |  |  |  |  |  |  |  |  |  |
| 1 | Vitvi04g00595\_t001 |  | \| \| |  |  |  |  |  |  |  |  |  |  |
| 1 | Vitvi04g00596\_t001 |  | Vitvi11g00694\_t002 |  |  |  |  |  |  |  |  |  |  |
| 1 | Vitvi04g00597\_t001 |  | \| \| |  |  |  |  |  |  |  |  |  |  |
| 1 | Vitvi04g00598\_t001 |  | \| \| |  |  |  |  |  |  |  |  |  |  |
| 1 | Vitvi04g00599\_t001 |  | \| \| |  |  |  |  |  |  |  |  |  |  |
| 1 | Vitvi04g04142\_t001 |  | \| \| |  |  |  |  |  |  |  |  |  |  |
| 1 | Vitvi04g00600\_t001 |  | Vitvi11g00700\_t001 |  |  |  |  |  |  |  |  |  |  |
| 1 | Vitvi04g00601\_t001 |  | Vitvi11g04176\_t001 |  |  |  |  |  |  |  |  |  |  |
| 1 | Vitvi04g00602\_t001 |  | Vitvi11g00708\_t001 |  |  |  |  |  |  |  |  |  |  |
| 1 | Vitvi04g00603\_t001 |  | \| \| |  |  |  |  |  |  |  |  |  |  |
| 1 | Vitvi04g00605\_t001 |  | \| \| |  |  |  |  |  |  |  |  |  |  |
| 1 | Vitvi04g01929\_t001 |  | Vitvi11g01505\_t001 |  |  |  |  |  |  |  |  |  |  |
| 1 | Vitvi04g01930\_t001 |  | Vitvi11g00715\_t001 |  |  |  |  |  |  |  |  |  |  |
| 1 | Vitvi04g00606\_t001 |  | \| \| |  |  |  |  |  |  |  |  |  |  |
| 1 | Vitvi04g04143\_t001 |  | \| \| |  |  |  |  |  |  |  |  |  |  |
| 1 | Vitvi04g00607\_t001 |  | \| \| |  |  |  |  |  |  |  |  |  |  |
| 1 | Vitvi04g00608\_t001 |  | \| \| |  |  |  |  |  |  |  |  |  |  |
| 1 | Vitvi04g00609\_t001 |  | Vitvi11g00723\_t001 |  |  |  |  |  |  |  |  |  |  |
| 0 | Vitvi04g00610\_t001 |  |  |  |  |  |  |  |  |  |  |  |  |
| 0 | Vitvi04g00611\_t001 |  |  |  |  |  |  |  |  |  |  |  |  |
| 0 | Vitvi04g00612\_t001 |  |  |  |  |  |  |  |  |  |  |  |  |
| 0 | Vitvi04g00613\_t001 |  |  |  |  |  |  |  |  |  |  |  |  |
| 0 | Vitvi04g00614\_t001 |  |  |  |  |  |  |  |  |  |  |  |  |
| 0 | Vitvi04g00616\_t002 |  |  |  |  |  |  |  |  |  |  |  |  |
| 0 | Vitvi04g00617\_t001 |  |  |  |  |  |  |  |  |  |  |  |  |
| 0 | Vitvi04g00618\_t001 |  |  |  |  |  |  |  |  |  |  |  |  |
| 0 | Vitvi04g00619\_t001 |  |  |  |  |  |  |  |  |  |  |  |  |
| 0 | Vitvi04g00620\_t001 |  |  |  |  |  |  |  |  |  |  |  |  |
| 0 | Vitvi04g04144\_t001 |  |  |  |  |  |  |  |  |  |  |  |  |
| 0 | Vitvi04g04145\_t001 |  |  |  |  |  |  |  |  |  |  |  |  |
| 0 | Vitvi04g00623\_t001 |  |  |  |  |  |  |  |  |  |  |  |  |
| 0 | Vitvi04g00625\_t001 |  |  |  |  |  |  |  |  |  |  |  |  |
| 0 | Vitvi04g00626\_t001 |  |  |  |  |  |  |  |  |  |  |  |  |
| 0 | Vitvi04g00627\_t001 |  |  |  |  |  |  |  |  |  |  |  |  |
| 0 | Vitvi04g00628\_t001 |  |  |  |  |  |  |  |  |  |  |  |  |
| 0 | Vitvi04g00629\_t001 |  |  |  |  |  |  |  |  |  |  |  |  |
| 0 | Vitvi04g00632\_t001 |  |  |  |  |  |  |  |  |  |  |  |  |
| 0 | Vitvi04g00633\_t001 |  |  |  |  |  |  |  |  |  |  |  |  |
| 0 | Vitvi04g00635\_t001 |  |  |  |  |  |  |  |  |  |  |  |  |
| 0 | Vitvi04g00636\_t001 |  |  |  |  |  |  |  |  |  |  |  |  |
| 0 | Vitvi04g04146\_t001 |  |  |  |  |  |  |  |  |  |  |  |  |
| 0 | Vitvi04g04147\_t001 |  |  |  |  |  |  |  |  |  |  |  |  |
| 0 | Vitvi04g00639\_t001 |  |  |  |  |  |  |  |  |  |  |  |  |
| 0 | Vitvi04g00640\_t001 |  |  |  |  |  |  |  |  |  |  |  |  |
| 0 | Vitvi04g00641\_t001 |  |  |  |  |  |  |  |  |  |  |  |  |
| 0 | Vitvi04g04148\_t001 |  |  |  |  |  |  |  |  |  |  |  |  |
| 0 | Vitvi04g01934\_t001 |  |  |  |  |  |  |  |  |  |  |  |  |
| 0 | Vitvi04g01935\_t001 |  |  |  |  |  |  |  |  |  |  |  |  |
| 0 | Vitvi04g01936\_t001 |  |  |  |  |  |  |  |  |  |  |  |  |
| 0 | Vitvi04g04149\_t001 |  |  |  |  |  |  |  |  |  |  |  |  |
| 0 | Vitvi04g01937\_t001 |  |  |  |  |  |  |  |  |  |  |  |  |
| 0 | Vitvi04g04150\_t001 |  |  |  |  |  |  |  |  |  |  |  |  |
| 0 | Vitvi04g00644\_t001 |  |  |  |  |  |  |  |  |  |  |  |  |
| 0 | Vitvi04g00645\_t001 |  |  |  |  |  |  |  |  |  |  |  |  |
| 0 | Vitvi04g00646\_t001 |  |  |  |  |  |  |  |  |  |  |  |  |
| 0 | Vitvi04g04151\_t001 |  |  |  |  |  |  |  |  |  |  |  |  |
| 0 | Vitvi04g04152\_t001 |  |  |  |  |  |  |  |  |  |  |  |  |
| 0 | Vitvi04g04153\_t001 |  |  |  |  |  |  |  |  |  |  |  |  |
| 0 | Vitvi04g00653\_t001 |  |  |  |  |  |  |  |  |  |  |  |  |
| 0 | Vitvi04g04154\_t001 |  |  |  |  |  |  |  |  |  |  |  |  |
| 0 | Vitvi04g04155\_t001 |  |  |  |  |  |  |  |  |  |  |  |  |
| 0 | Vitvi04g01940\_t001 |  |  |  |  |  |  |  |  |  |  |  |  |
| 0 | Vitvi04g04156\_t001 |  |  |  |  |  |  |  |  |  |  |  |  |
| 0 | Vitvi04g01941\_t001 |  |  |  |  |  |  |  |  |  |  |  |  |
| 0 | Vitvi04g04157\_t001 |  |  |  |  |  |  |  |  |  |  |  |  |
| 0 | Vitvi04g00658\_t001 |  |  |  |  |  |  |  |  |  |  |  |  |
| 0 | Vitvi04g04158\_t001 |  |  |  |  |  |  |  |  |  |  |  |  |
| 0 | Vitvi04g01943\_t001 |  |  |  |  |  |  |  |  |  |  |  |  |
| 0 | Vitvi04g04159\_t001 |  |  |  |  |  |  |  |  |  |  |  |  |
| 0 | Vitvi04g00660\_t001 |  |  |  |  |  |  |  |  |  |  |  |  |
| 0 | Vitvi04g00661\_t001 |  |  |  |  |  |  |  |  |  |  |  |  |
| 0 | Vitvi04g00662\_t001 |  |  |  |  |  |  |  |  |  |  |  |  |
| 0 | Vitvi04g00664\_t001 |  |  |  |  |  |  |  |  |  |  |  |  |
| 0 | Vitvi04g00665\_t001 |  |  |  |  |  |  |  |  |  |  |  |  |
| 0 | Vitvi04g00669\_t001 |  |  |  |  |  |  |  |  |  |  |  |  |
| 0 | Vitvi04g00670\_t001 |  |  |  |  |  |  |  |  |  |  |  |  |
| 0 | Vitvi04g00671\_t001 |  |  |  |  |  |  |  |  |  |  |  |  |
| 0 | Vitvi04g00672\_t001 |  |  |  |  |  |  |  |  |  |  |  |  |
| 0 | Vitvi04g00673\_t001 |  |  |  |  |  |  |  |  |  |  |  |  |
| 0 | Vitvi04g00680\_t001 |  |  |  |  |  |  |  |  |  |  |  |  |
| 0 | Vitvi04g04160\_t001 |  |  |  |  |  |  |  |  |  |  |  |  |
| 0 | Vitvi04g00681\_t001 |  |  |  |  |  |  |  |  |  |  |  |  |
| 0 | Vitvi04g00685\_t001 |  |  |  |  |  |  |  |  |  |  |  |  |
| 0 | Vitvi04g00688\_t001 |  |  |  |  |  |  |  |  |  |  |  |  |
| 0 | Vitvi04g01948\_t001 |  |  |  |  |  |  |  |  |  |  |  |  |
| 0 | Vitvi04g00689\_t001 |  |  |  |  |  |  |  |  |  |  |  |  |
| 0 | Vitvi04g00693\_t001 |  |  |  |  |  |  |  |  |  |  |  |  |
| 0 | Vitvi04g04161\_t001 |  |  |  |  |  |  |  |  |  |  |  |  |
| 0 | Vitvi04g00696\_t001 |  |  |  |  |  |  |  |  |  |  |  |  |
| 0 | Vitvi04g04162\_t001 |  |  |  |  |  |  |  |  |  |  |  |  |
| 0 | Vitvi04g04163\_t001 |  |  |  |  |  |  |  |  |  |  |  |  |
| 0 | Vitvi04g04164\_t001 |  |  |  |  |  |  |  |  |  |  |  |  |
| 0 | Vitvi04g00703\_t001 |  |  |  |  |  |  |  |  |  |  |  |  |
| 0 | Vitvi04g04165\_t001 |  |  |  |  |  |  |  |  |  |  |  |  |
| 0 | Vitvi04g04166\_t001 |  |  |  |  |  |  |  |  |  |  |  |  |
| 0 | Vitvi04g00707\_t001 |  |  |  |  |  |  |  |  |  |  |  |  |
| 0 | Vitvi04g01955\_t001 |  |  |  |  |  |  |  |  |  |  |  |  |
| 0 | Vitvi04g04167\_t001 |  |  |  |  |  |  |  |  |  |  |  |  |
| 0 | Vitvi04g04168\_t001 |  |  |  |  |  |  |  |  |  |  |  |  |
| 0 | Vitvi04g04169\_t001 |  |  |  |  |  |  |  |  |  |  |  |  |
| 0 | Vitvi04g00710\_t001 |  |  |  |  |  |  |  |  |  |  |  |  |
| 0 | Vitvi04g04170\_t001 |  |  |  |  |  |  |  |  |  |  |  |  |
| 0 | Vitvi04g04171\_t001 |  |  |  |  |  |  |  |  |  |  |  |  |
| 0 | Vitvi04g04172\_t001 |  |  |  |  |  |  |  |  |  |  |  |  |
| 0 | Vitvi04g00712\_t001 |  |  |  |  |  |  |  |  |  |  |  |  |
| 0 | Vitvi04g04173\_t001 |  |  |  |  |  |  |  |  |  |  |  |  |
| 0 | Vitvi04g04174\_t001 |  |  |  |  |  |  |  |  |  |  |  |  |
| 0 | Vitvi04g04175\_t001 |  |  |  |  |  |  |  |  |  |  |  |  |
| 0 | Vitvi04g04176\_t001 |  |  |  |  |  |  |  |  |  |  |  |  |
| 0 | Vitvi04g01965\_t001 |  |  |  |  |  |  |  |  |  |  |  |  |
| 0 | Vitvi04g04177\_t001 |  |  |  |  |  |  |  |  |  |  |  |  |
| 0 | Vitvi04g04178\_t001 |  |  |  |  |  |  |  |  |  |  |  |  |
| 0 | Vitvi04g00715\_t001 |  |  |  |  |  |  |  |  |  |  |  |  |
| 0 | Vitvi04g00716\_t001 |  |  |  |  |  |  |  |  |  |  |  |  |
| 0 | Vitvi04g04179\_t001 |  |  |  |  |  |  |  |  |  |  |  |  |
| 0 | Vitvi04g04180\_t001 |  |  |  |  |  |  |  |  |  |  |  |  |
| 0 | Vitvi04g04181\_t001 |  |  |  |  |  |  |  |  |  |  |  |  |
| 0 | Vitvi04g04182\_t001 |  |  |  |  |  |  |  |  |  |  |  |  |
| 0 | Vitvi04g00725\_t001 |  |  |  |  |  |  |  |  |  |  |  |  |
| 0 | Vitvi04g04183\_t001 |  |  |  |  |  |  |  |  |  |  |  |  |
| 0 | Vitvi04g01974\_t001 |  |  |  |  |  |  |  |  |  |  |  |  |
| 0 | Vitvi04g04184\_t001 |  |  |  |  |  |  |  |  |  |  |  |  |
| 0 | Vitvi04g04185\_t001 |  |  |  |  |  |  |  |  |  |  |  |  |
| 0 | Vitvi04g00728\_t001 |  |  |  |  |  |  |  |  |  |  |  |  |
| 0 | Vitvi04g04186\_t001 |  |  |  |  |  |  |  |  |  |  |  |  |
| 0 | Vitvi04g00732\_t001 |  |  |  |  |  |  |  |  |  |  |  |  |
| 0 | Vitvi04g04187\_t001 |  |  |  |  |  |  |  |  |  |  |  |  |
| 0 | Vitvi04g00734\_t001 |  |  |  |  |  |  |  |  |  |  |  |  |
| 0 | Vitvi04g00735\_t003 |  |  |  |  |  |  |  |  |  |  |  |  |
| 0 | Vitvi04g00736\_t001 |  |  |  |  |  |  |  |  |  |  |  |  |
| 0 | Vitvi04g04188\_t003 |  |  |  |  |  |  |  |  |  |  |  |  |
| 0 | Vitvi04g01978\_t001 |  |  |  |  |  |  |  |  |  |  |  |  |
| 0 | Vitvi04g04189\_t001 |  |  |  |  |  |  |  |  |  |  |  |  |
| 0 | Vitvi04g00737\_t001 |  |  |  |  |  |  |  |  |  |  |  |  |
| 0 | Vitvi04g00740\_t001 |  |  |  |  |  |  |  |  |  |  |  |  |
| 0 | Vitvi04g00741\_t001 |  |  |  |  |  |  |  |  |  |  |  |  |
| 0 | Vitvi04g00742\_t001 |  |  |  |  |  |  |  |  |  |  |  |  |
| 0 | Vitvi04g04190\_t001 |  |  |  |  |  |  |  |  |  |  |  |  |
| 0 | Vitvi04g04191\_t001 |  |  |  |  |  |  |  |  |  |  |  |  |
| 0 | Vitvi04g00747\_t001 |  |  |  |  |  |  |  |  |  |  |  |  |
| 0 | Vitvi04g00748\_t002 |  |  |  |  |  |  |  |  |  |  |  |  |
| 0 | Vitvi04g01979\_t001 |  |  |  |  |  |  |  |  |  |  |  |  |
| 0 | Vitvi04g04192\_t001 |  |  |  |  |  |  |  |  |  |  |  |  |
| 0 | Vitvi04g00749\_t001 |  |  |  |  |  |  |  |  |  |  |  |  |
| 0 | Vitvi04g04193\_t001 |  |  |  |  |  |  |  |  |  |  |  |  |
| 0 | Vitvi04g04194\_t001 |  |  |  |  |  |  |  |  |  |  |  |  |
| 0 | Vitvi04g00750\_t001 |  |  |  |  |  |  |  |  |  |  |  |  |
| 0 | Vitvi04g01980\_t001 |  |  |  |  |  |  |  |  |  |  |  |  |
| 0 | Vitvi04g04195\_t001 |  |  |  |  |  |  |  |  |  |  |  |  |
| 0 | Vitvi04g04196\_t001 |  |  |  |  |  |  |  |  |  |  |  |  |
| 0 | Vitvi04g04197\_t001 |  |  |  |  |  |  |  |  |  |  |  |  |
| 0 | Vitvi04g04198\_t001 |  |  |  |  |  |  |  |  |  |  |  |  |
| 0 | Vitvi04g04199\_t001 |  |  |  |  |  |  |  |  |  |  |  |  |
| 0 | Vitvi04g04200\_t001 |  |  |  |  |  |  |  |  |  |  |  |  |
| 0 | Vitvi04g04201\_t001 |  |  |  |  |  |  |  |  |  |  |  |  |
| 0 | Vitvi04g04202\_t001 |  |  |  |  |  |  |  |  |  |  |  |  |
| 0 | Vitvi04g01983\_t001 |  |  |  |  |  |  |  |  |  |  |  |  |
| 0 | Vitvi04g04203\_t001 |  |  |  |  |  |  |  |  |  |  |  |  |
| 0 | Vitvi04g00756\_t001 |  |  |  |  |  |  |  |  |  |  |  |  |
| 0 | Vitvi04g04204\_t001 |  |  |  |  |  |  |  |  |  |  |  |  |
| 0 | Vitvi04g00758\_t001 |  |  |  |  |  |  |  |  |  |  |  |  |
| 0 | Vitvi04g00759\_t001 |  |  |  |  |  |  |  |  |  |  |  |  |
| 0 | Vitvi04g04205\_t001 |  |  |  |  |  |  |  |  |  |  |  |  |
| 0 | Vitvi04g01984\_t001.1.6037826d |  |  |  |  |  |  |  |  |  |  |  |  |
| 0 | Vitvi04g00760\_t001 |  |  |  |  |  |  |  |  |  |  |  |  |
| 0 | Vitvi04g01985\_t001 |  |  |  |  |  |  |  |  |  |  |  |  |
| 0 | Vitvi04g00761\_t001 |  |  |  |  |  |  |  |  |  |  |  |  |
| 0 | Vitvi04g00762\_t001 |  |  |  |  |  |  |  |  |  |  |  |  |
| 0 | Vitvi04g00764\_t001 |  |  |  |  |  |  |  |  |  |  |  |  |
| 0 | Vitvi04g00765\_t001 |  |  |  |  |  |  |  |  |  |  |  |  |
| 0 | Vitvi04g01986\_t001 |  |  |  |  |  |  |  |  |  |  |  |  |
| 0 | Vitvi04g00767\_t001 |  |  |  |  |  |  |  |  |  |  |  |  |
| 0 | Vitvi04g00769\_t002 |  |  |  |  |  |  |  |  |  |  |  |  |
| 0 | Vitvi04g04206\_t001 |  |  |  |  |  |  |  |  |  |  |  |  |
| 0 | Vitvi04g04207\_t001 |  |  |  |  |  |  |  |  |  |  |  |  |
| 0 | Vitvi04g04208\_t001 |  |  |  |  |  |  |  |  |  |  |  |  |
| 0 | Vitvi04g01989\_t001 |  |  |  |  |  |  |  |  |  |  |  |  |
| 0 | Vitvi04g00770\_t001 |  |  |  |  |  |  |  |  |  |  |  |  |
| 0 | Vitvi04g00771\_t001 |  |  |  |  |  |  |  |  |  |  |  |  |
| 0 | Vitvi04g04209\_t001 |  |  |  |  |  |  |  |  |  |  |  |  |
| 0 | Vitvi04g04210\_t001 |  |  |  |  |  |  |  |  |  |  |  |  |
| 0 | Vitvi04g04211\_t001 |  |  |  |  |  |  |  |  |  |  |  |  |
| 0 | Vitvi04g04212\_t001 |  |  |  |  |  |  |  |  |  |  |  |  |
| 0 | Vitvi04g00781\_t001 |  |  |  |  |  |  |  |  |  |  |  |  |
| 0 | Vitvi04g04213\_t001 |  |  |  |  |  |  |  |  |  |  |  |  |
| 0 | Vitvi04g04214\_t001 |  |  |  |  |  |  |  |  |  |  |  |  |
| 0 | Vitvi04g01992\_t001 |  |  |  |  |  |  |  |  |  |  |  |  |
| 0 | Vitvi04g00785\_t001 |  |  |  |  |  |  |  |  |  |  |  |  |
| 0 | Vitvi04g00786\_t001 |  |  |  |  |  |  |  |  |  |  |  |  |
| 0 | Vitvi04g01993\_t001 |  |  |  |  |  |  |  |  |  |  |  |  |
| 0 | Vitvi04g00803\_t001 |  |  |  |  |  |  |  |  |  |  |  |  |
| 0 | Vitvi04g00807\_t001 |  |  |  |  |  |  |  |  |  |  |  |  |
| 0 | Vitvi04g04215\_t001 |  |  |  |  |  |  |  |  |  |  |  |  |
| 0 | Vitvi04g04216\_t001 |  |  |  |  |  |  |  |  |  |  |  |  |
| 0 | Vitvi04g04217\_t001 |  |  |  |  |  |  |  |  |  |  |  |  |
| 0 | Vitvi04g04218\_t001 |  |  |  |  |  |  |  |  |  |  |  |  |
| 0 | Vitvi04g00809\_t001 |  |  |  |  |  |  |  |  |  |  |  |  |
| 0 | Vitvi04g00815\_t001 |  |  |  |  |  |  |  |  |  |  |  |  |
| 0 | Vitvi04g00817\_t001 |  |  |  |  |  |  |  |  |  |  |  |  |
| 0 | Vitvi04g01999\_t001 |  |  |  |  |  |  |  |  |  |  |  |  |
| 0 | Vitvi04g04219\_t001 |  |  |  |  |  |  |  |  |  |  |  |  |
| 0 | Vitvi04g04220\_t001 |  |  |  |  |  |  |  |  |  |  |  |  |
| 0 | Vitvi04g04221\_t001 |  |  |  |  |  |  |  |  |  |  |  |  |
| 0 | Vitvi04g04222\_t001 |  |  |  |  |  |  |  |  |  |  |  |  |
| 0 | Vitvi04g00824\_t001 |  |  |  |  |  |  |  |  |  |  |  |  |
| 0 | Vitvi04g04223\_t001 |  |  |  |  |  |  |  |  |  |  |  |  |
| 0 | Vitvi04g02000\_t001 |  |  |  |  |  |  |  |  |  |  |  |  |
| 0 | Vitvi04g00827\_t001 |  |  |  |  |  |  |  |  |  |  |  |  |
| 0 | Vitvi04g04224\_t001 |  |  |  |  |  |  |  |  |  |  |  |  |
| 0 | Vitvi04g00829\_t001 |  |  |  |  |  |  |  |  |  |  |  |  |
| 0 | Vitvi04g04225\_t001 |  |  |  |  |  |  |  |  |  |  |  |  |
| 0 | Vitvi04g00831\_t001 |  |  |  |  |  |  |  |  |  |  |  |  |
| 0 | Vitvi04g00832\_t001 |  |  |  |  |  |  |  |  |  |  |  |  |
| 0 | Vitvi04g04226\_t001 |  |  |  |  |  |  |  |  |  |  |  |  |
| 0 | Vitvi04g00836\_t001 |  |  |  |  |  |  |  |  |  |  |  |  |
| 0 | Vitvi04g04227\_t001 |  |  |  |  |  |  |  |  |  |  |  |  |
| 0 | Vitvi04g04228\_t001 |  |  |  |  |  |  |  |  |  |  |  |  |
| 0 | Vitvi04g00837\_t001 |  |  |  |  |  |  |  |  |  |  |  |  |
| 0 | Vitvi04g04229\_t001 |  |  |  |  |  |  |  |  |  |  |  |  |
| 0 | Vitvi04g00838\_t001 |  |  |  |  |  |  |  |  |  |  |  |  |
| 0 | Vitvi04g02004\_t001 |  |  |  |  |  |  |  |  |  |  |  |  |
| 0 | Vitvi04g00839\_t001 |  |  |  |  |  |  |  |  |  |  |  |  |
| 0 | Vitvi04g04230\_t001 |  |  |  |  |  |  |  |  |  |  |  |  |
| 0 | Vitvi04g00840\_t001 |  |  |  |  |  |  |  |  |  |  |  |  |
| 0 | Vitvi04g04231\_t001 |  |  |  |  |  |  |  |  |  |  |  |  |
| 0 | Vitvi04g00841\_t001 |  |  |  |  |  |  |  |  |  |  |  |  |
| 0 | Vitvi04g04232\_t001 |  |  |  |  |  |  |  |  |  |  |  |  |
| 0 | Vitvi04g04233\_t001 |  |  |  |  |  |  |  |  |  |  |  |  |
| 0 | Vitvi04g04234\_t001 |  |  |  |  |  |  |  |  |  |  |  |  |
| 0 | Vitvi04g00844\_t001 |  |  |  |  |  |  |  |  |  |  |  |  |
| 0 | Vitvi04g04235\_t001 |  |  |  |  |  |  |  |  |  |  |  |  |
| 0 | Vitvi04g04236\_t001 |  |  |  |  |  |  |  |  |  |  |  |  |
| 0 | Vitvi04g04237\_t001 |  |  |  |  |  |  |  |  |  |  |  |  |
| 0 | Vitvi04g04238\_t001 |  |  |  |  |  |  |  |  |  |  |  |  |
| 0 | Vitvi04g00845\_t001 |  |  |  |  |  |  |  |  |  |  |  |  |
| 0 | Vitvi04g00846\_t001 |  |  |  |  |  |  |  |  |  |  |  |  |
| 0 | Vitvi04g04239\_t001 |  |  |  |  |  |  |  |  |  |  |  |  |
| 0 | Vitvi04g00847\_t001 |  |  |  |  |  |  |  |  |  |  |  |  |
| 0 | Vitvi04g00854\_t001 |  |  |  |  |  |  |  |  |  |  |  |  |
| 0 | Vitvi04g00859\_t001 |  |  |  |  |  |  |  |  |  |  |  |  |
| 0 | Vitvi04g00860\_t001 |  |  |  |  |  |  |  |  |  |  |  |  |
| 0 | Vitvi04g00861\_t001 |  |  |  |  |  |  |  |  |  |  |  |  |
| 0 | Vitvi04g00862\_t001 |  |  |  |  |  |  |  |  |  |  |  |  |
| 0 | Vitvi04g00863\_t001 |  |  |  |  |  |  |  |  |  |  |  |  |
| 0 | Vitvi04g04240\_t001 |  |  |  |  |  |  |  |  |  |  |  |  |
| 0 | Vitvi04g00870\_t001 |  |  |  |  |  |  |  |  |  |  |  |  |
| 0 | Vitvi04g00871\_t001 |  |  |  |  |  |  |  |  |  |  |  |  |
| 0 | Vitvi04g02010\_t001 |  |  |  |  |  |  |  |  |  |  |  |  |
| 0 | Vitvi04g04241\_t001 |  |  |  |  |  |  |  |  |  |  |  |  |
| 0 | Vitvi04g00878\_t001 |  |  |  |  |  |  |  |  |  |  |  |  |
| 0 | Vitvi04g04242\_t001 |  |  |  |  |  |  |  |  |  |  |  |  |
| 0 | Vitvi04g02011\_t001 |  |  |  |  |  |  |  |  |  |  |  |  |
| 0 | Vitvi04g00880\_t001 |  |  |  |  |  |  |  |  |  |  |  |  |
| 0 | Vitvi04g04243\_t001 |  |  |  |  |  |  |  |  |  |  |  |  |
| 0 | Vitvi04g02013\_t001 |  |  |  |  |  |  |  |  |  |  |  |  |
| 0 | Vitvi04g04244\_t001 |  |  |  |  |  |  |  |  |  |  |  |  |
| 0 | Vitvi04g04245\_t001 |  |  |  |  |  |  |  |  |  |  |  |  |
| 0 | Vitvi04g00884\_t001 |  |  |  |  |  |  |  |  |  |  |  |  |
| 0 | Vitvi04g04246\_t001 |  |  |  |  |  |  |  |  |  |  |  |  |
| 0 | Vitvi04g00886\_t001 |  |  |  |  |  |  |  |  |  |  |  |  |
| 0 | Vitvi04g02015\_t001 |  |  |  |  |  |  |  |  |  |  |  |  |
| 0 | Vitvi04g04247\_t001 |  |  |  |  |  |  |  |  |  |  |  |  |
| 0 | Vitvi04g00889\_t001 |  |  |  |  |  |  |  |  |  |  |  |  |
| 0 | Vitvi04g00890\_t001 |  |  |  |  |  |  |  |  |  |  |  |  |
| 0 | Vitvi04g04248\_t001 |  |  |  |  |  |  |  |  |  |  |  |  |
| 0 | Vitvi04g04249\_t001 |  |  |  |  |  |  |  |  |  |  |  |  |
| 0 | Vitvi04g04250\_t001 |  |  |  |  |  |  |  |  |  |  |  |  |
| 0 | Vitvi04g00897\_t001 |  |  |  |  |  |  |  |  |  |  |  |  |
| 0 | Vitvi04g04251\_t001 |  |  |  |  |  |  |  |  |  |  |  |  |
| 0 | Vitvi04g02017\_t001 |  |  |  |  |  |  |  |  |  |  |  |  |
| 0 | Vitvi04g04252\_t001 |  |  |  |  |  |  |  |  |  |  |  |  |
| 0 | Vitvi04g00901\_t001 |  |  |  |  |  |  |  |  |  |  |  |  |
| 0 | Vitvi04g04253\_t001 |  |  |  |  |  |  |  |  |  |  |  |  |
| 0 | Vitvi04g04254\_t001 |  |  |  |  |  |  |  |  |  |  |  |  |
| 0 | Vitvi04g04255\_t001 |  |  |  |  |  |  |  |  |  |  |  |  |
| 0 | Vitvi04g04256\_t001 |  |  |  |  |  |  |  |  |  |  |  |  |
| 0 | Vitvi04g04257\_t001 |  |  |  |  |  |  |  |  |  |  |  |  |
| 0 | Vitvi04g04258\_t001 |  |  |  |  |  |  |  |  |  |  |  |  |
| 0 | Vitvi04g04259\_t001 |  |  |  |  |  |  |  |  |  |  |  |  |
| 0 | Vitvi04g04260\_t001 |  |  |  |  |  |  |  |  |  |  |  |  |
| 0 | Vitvi04g00907\_t003 |  |  |  |  |  |  |  |  |  |  |  |  |
| 0 | Vitvi04g00908\_t001 |  |  |  |  |  |  |  |  |  |  |  |  |
| 0 | Vitvi04g00909\_t001 |  |  |  |  |  |  |  |  |  |  |  |  |
| 0 | Vitvi04g00911\_t001 |  |  |  |  |  |  |  |  |  |  |  |  |
| 0 | Vitvi04g00912\_t001 |  |  |  |  |  |  |  |  |  |  |  |  |
| 0 | Vitvi04g00913\_t001 |  |  |  |  |  |  |  |  |  |  |  |  |
| 0 | Vitvi04g04261\_t001 |  |  |  |  |  |  |  |  |  |  |  |  |
| 0 | Vitvi04g04262\_t001 |  |  |  |  |  |  |  |  |  |  |  |  |
| 0 | Vitvi04g04263\_t001 |  |  |  |  |  |  |  |  |  |  |  |  |
| 0 | Vitvi04g04264\_t001 |  |  |  |  |  |  |  |  |  |  |  |  |
| 0 | Vitvi04g04265\_t001 |  |  |  |  |  |  |  |  |  |  |  |  |
| 0 | Vitvi04g04266\_t001 |  |  |  |  |  |  |  |  |  |  |  |  |
| 0 | Vitvi04g02022\_t001 |  |  |  |  |  |  |  |  |  |  |  |  |
| 0 | Vitvi04g04267\_t001 |  |  |  |  |  |  |  |  |  |  |  |  |
| 0 | Vitvi04g04268\_t001 |  |  |  |  |  |  |  |  |  |  |  |  |
| 0 | Vitvi04g00929\_t001 |  |  |  |  |  |  |  |  |  |  |  |  |
| 0 | Vitvi04g00932\_t001 |  |  |  |  |  |  |  |  |  |  |  |  |
| 0 | Vitvi04g04269\_t001 |  |  |  |  |  |  |  |  |  |  |  |  |
| 0 | Vitvi04g00937\_t001 |  |  |  |  |  |  |  |  |  |  |  |  |
| 0 | Vitvi04g04270\_t001 |  |  |  |  |  |  |  |  |  |  |  |  |
| 0 | Vitvi04g04271\_t001 |  |  |  |  |  |  |  |  |  |  |  |  |
| 0 | Vitvi04g04272\_t001 |  |  |  |  |  |  |  |  |  |  |  |  |
| 0 | Vitvi04g00943\_t001 |  |  |  |  |  |  |  |  |  |  |  |  |
| 0 | Vitvi04g00947\_t001 |  |  |  |  |  |  |  |  |  |  |  |  |
| 0 | Vitvi04g00949\_t001 |  |  |  |  |  |  |  |  |  |  |  |  |
| 0 | Vitvi04g02024\_t001 |  |  |  |  |  |  |  |  |  |  |  |  |
| 0 | Vitvi04g00953\_t001 |  |  |  |  |  |  |  |  |  |  |  |  |
| 0 | Vitvi04g04273\_t001 |  |  |  |  |  |  |  |  |  |  |  |  |
| 0 | Vitvi04g00956\_t001 |  |  |  |  |  |  |  |  |  |  |  |  |
| 0 | Vitvi04g04274\_t001 |  |  |  |  |  |  |  |  |  |  |  |  |
| 0 | Vitvi04g00958\_t001 |  |  |  |  |  |  |  |  |  |  |  |  |
| 0 | Vitvi04g00960\_t001 |  |  |  |  |  |  |  |  |  |  |  |  |
| 0 | Vitvi04g00962\_t001 |  |  |  |  |  |  |  |  |  |  |  |  |
| 0 | Vitvi04g04275\_t001 |  |  |  |  |  |  |  |  |  |  |  |  |
| 0 | Vitvi04g02027\_t001 |  |  |  |  |  |  |  |  |  |  |  |  |
| 0 | Vitvi04g00965\_t002 |  |  |  |  |  |  |  |  |  |  |  |  |
| 0 | Vitvi04g00970\_t001 |  |  |  |  |  |  |  |  |  |  |  |  |
| 0 | Vitvi04g00972\_t001 |  |  |  |  |  |  |  |  |  |  |  |  |
| 0 | Vitvi04g00973\_t001 |  |  |  |  |  |  |  |  |  |  |  |  |
| 0 | Vitvi04g00974\_t001 |  |  |  |  |  |  |  |  |  |  |  |  |
| 0 | Vitvi04g02028\_t001 |  |  |  |  |  |  |  |  |  |  |  |  |
| 0 | Vitvi04g00979\_t001 |  |  |  |  |  |  |  |  |  |  |  |  |
| 0 | Vitvi04g00980\_t001 |  |  |  |  |  |  |  |  |  |  |  |  |
| 0 | Vitvi04g04276\_t001 |  |  |  |  |  |  |  |  |  |  |  |  |
| 0 | Vitvi04g04277\_t001 |  |  |  |  |  |  |  |  |  |  |  |  |
| 0 | Vitvi04g02031\_t001 |  |  |  |  |  |  |  |  |  |  |  |  |
| 0 | Vitvi04g00983\_t001 |  |  |  |  |  |  |  |  |  |  |  |  |
| 0 | Vitvi04g04278\_t001 |  |  |  |  |  |  |  |  |  |  |  |  |
| 0 | Vitvi04g04279\_t001 |  |  |  |  |  |  |  |  |  |  |  |  |
| 0 | Vitvi04g02033\_t001 |  |  |  |  |  |  |  |  |  |  |  |  |
| 0 | Vitvi04g04280\_t001 |  |  |  |  |  |  |  |  |  |  |  |  |
| 0 | Vitvi04g04281\_t001 |  |  |  |  |  |  |  |  |  |  |  |  |
| 0 | Vitvi04g00988\_t001 |  |  |  |  |  |  |  |  |  |  |  |  |
| 0 | Vitvi04g00989\_t001 |  |  |  |  |  |  |  |  |  |  |  |  |
| 0 | Vitvi04g00990\_t001 |  |  |  |  |  |  |  |  |  |  |  |  |
| 0 | Vitvi04g00991\_t001 |  |  |  |  |  |  |  |  |  |  |  |  |
| 0 | Vitvi04g00992\_t001 |  |  |  |  |  |  |  |  |  |  |  |  |
| 0 | Vitvi04g04282\_t001 |  |  |  |  |  |  |  |  |  |  |  |  |
| 0 | Vitvi04g00994\_t001 |  |  |  |  |  |  |  |  |  |  |  |  |
| 0 | Vitvi04g00997\_t001 |  |  |  |  |  |  |  |  |  |  |  |  |
| 0 | Vitvi04g00998\_t001 |  |  |  |  |  |  |  |  |  |  |  |  |
| 0 | Vitvi04g00999\_t001 |  |  |  |  |  |  |  |  |  |  |  |  |
| 0 | Vitvi04g01001\_t001 |  |  |  |  |  |  |  |  |  |  |  |  |
| 0 | Vitvi04g01004\_t001 |  |  |  |  |  |  |  |  |  |  |  |  |
| 0 | Vitvi04g01007\_t001 |  |  |  |  |  |  |  |  |  |  |  |  |
| 0 | Vitvi04g01011\_t001 |  |  |  |  |  |  |  |  |  |  |  |  |
| 0 | Vitvi04g01013\_t001 |  |  |  |  |  |  |  |  |  |  |  |  |
| 0 | Vitvi04g01014\_t001 |  |  |  |  |  |  |  |  |  |  |  |  |
| 0 | Vitvi04g02037\_t001 |  |  |  |  |  |  |  |  |  |  |  |  |
| 0 | Vitvi04g04283\_t001 |  |  |  |  |  |  |  |  |  |  |  |  |
| 0 | Vitvi04g01016\_t001 |  |  |  |  |  |  |  |  |  |  |  |  |
| 0 | Vitvi04g02038\_t001 |  |  |  |  |  |  |  |  |  |  |  |  |
| 0 | Vitvi04g04284\_t001 |  |  |  |  |  |  |  |  |  |  |  |  |
| 0 | Vitvi04g04285\_t001 |  |  |  |  |  |  |  |  |  |  |  |  |
| 0 | Vitvi04g01024\_t001 |  |  |  |  |  |  |  |  |  |  |  |  |
| 0 | Vitvi04g04286\_t001 |  |  |  |  |  |  |  |  |  |  |  |  |
| 0 | Vitvi04g02039\_t001 |  |  |  |  |  |  |  |  |  |  |  |  |
| 0 | Vitvi04g01026\_t001 |  |  |  |  |  |  |  |  |  |  |  |  |
| 0 | Vitvi04g02040\_t001 |  |  |  |  |  |  |  |  |  |  |  |  |
| 0 | Vitvi04g01029\_t001 |  |  |  |  |  |  |  |  |  |  |  |  |
| 0 | Vitvi04g04287\_t001 |  |  |  |  |  |  |  |  |  |  |  |  |
| 0 | Vitvi04g04288\_t001 |  |  |  |  |  |  |  |  |  |  |  |  |
| 0 | Vitvi04g01034\_t001 |  |  |  |  |  |  |  |  |  |  |  |  |
| 0 | Vitvi04g01035\_t001 |  |  |  |  |  |  |  |  |  |  |  |  |
| 0 | Vitvi04g01037\_t001 |  |  |  |  |  |  |  |  |  |  |  |  |
| 0 | Vitvi04g01039\_t001 |  |  |  |  |  |  |  |  |  |  |  |  |
| 0 | Vitvi04g01041\_t001 |  |  |  |  |  |  |  |  |  |  |  |  |
| 0 | Vitvi04g01044\_t001 |  |  |  |  |  |  |  |  |  |  |  |  |
| 0 | Vitvi04g01045\_t001 |  |  |  |  |  |  |  |  |  |  |  |  |
| 0 | Vitvi04g01047\_t001 |  |  |  |  |  |  |  |  |  |  |  |  |
| 0 | Vitvi04g01049\_t001 |  |  |  |  |  |  |  |  |  |  |  |  |
| 0 | Vitvi04g04289\_t001 |  |  |  |  |  |  |  |  |  |  |  |  |
| 0 | Vitvi04g01051\_t001 |  |  |  |  |  |  |  |  |  |  |  |  |
| 0 | Vitvi04g04290\_t001 |  |  |  |  |  |  |  |  |  |  |  |  |
| 0 | Vitvi04g01052\_t001 |  |  |  |  |  |  |  |  |  |  |  |  |
| 0 | Vitvi04g01054\_t001 |  |  |  |  |  |  |  |  |  |  |  |  |
| 0 | Vitvi04g01056\_t001 |  |  |  |  |  |  |  |  |  |  |  |  |
| 0 | Vitvi04g01057\_t001 |  |  |  |  |  |  |  |  |  |  |  |  |
| 0 | Vitvi04g01059\_t001 |  |  |  |  |  |  |  |  |  |  |  |  |
| 0 | Vitvi04g01061\_t001 |  |  |  |  |  |  |  |  |  |  |  |  |
| 0 | Vitvi04g04291\_t001 |  |  |  |  |  |  |  |  |  |  |  |  |
| 0 | Vitvi04g04292\_t001 |  |  |  |  |  |  |  |  |  |  |  |  |
| 0 | Vitvi04g02046\_t001 |  |  |  |  |  |  |  |  |  |  |  |  |
| 0 | Vitvi04g01068\_t001 |  |  |  |  |  |  |  |  |  |  |  |  |
| 0 | Vitvi04g01075\_t001 |  |  |  |  |  |  |  |  |  |  |  |  |
| 0 | Vitvi04g01077\_t001 |  |  |  |  |  |  |  |  |  |  |  |  |
| 0 | Vitvi04g01078\_t001 |  |  |  |  |  |  |  |  |  |  |  |  |
| 0 | Vitvi04g01080\_t001 |  |  |  |  |  |  |  |  |  |  |  |  |
| 0 | Vitvi04g04293\_t001 |  |  |  |  |  |  |  |  |  |  |  |  |
| 0 | Vitvi04g01082\_t003 |  |  |  |  |  |  |  |  |  |  |  |  |
| 0 | Vitvi04g04294\_t001 |  |  |  |  |  |  |  |  |  |  |  |  |
| 0 | Vitvi04g01084\_t001 |  |  |  |  |  |  |  |  |  |  |  |  |
| 0 | Vitvi04g01088\_t001 |  |  |  |  |  |  |  |  |  |  |  |  |
| 0 | Vitvi04g01089\_t001 |  |  |  |  |  |  |  |  |  |  |  |  |
| 0 | Vitvi04g04295\_t001 |  |  |  |  |  |  |  |  |  |  |  |  |
| 0 | Vitvi04g01092\_t001 |  |  |  |  |  |  |  |  |  |  |  |  |
| 0 | Vitvi04g01093\_t001 |  |  |  |  |  |  |  |  |  |  |  |  |
| 0 | Vitvi04g01095\_t001 |  |  |  |  |  |  |  |  |  |  |  |  |
| 0 | Vitvi04g02051\_t001 |  |  |  |  |  |  |  |  |  |  |  |  |
| 0 | Vitvi04g01097\_t001 |  |  |  |  |  |  |  |  |  |  |  |  |
| 0 | Vitvi04g01098\_t001 |  |  |  |  |  |  |  |  |  |  |  |  |
| 0 | Vitvi04g01100\_t001 |  |  |  |  |  |  |  |  |  |  |  |  |
| 0 | Vitvi04g01101\_t001 |  |  |  |  |  |  |  |  |  |  |  |  |
| 0 | Vitvi04g04296\_t001 |  |  |  |  |  |  |  |  |  |  |  |  |
| 0 | Vitvi04g01103\_t001 |  |  |  |  |  |  |  |  |  |  |  |  |
| 0 | Vitvi04g01104\_t001 |  |  |  |  |  |  |  |  |  |  |  |  |
| 0 | Vitvi04g01105\_t001 |  |  |  |  |  |  |  |  |  |  |  |  |
| 0 | Vitvi04g01106\_t001 |  |  |  |  |  |  |  |  |  |  |  |  |
| 0 | Vitvi04g01107\_t001 |  |  |  |  |  |  |  |  |  |  |  |  |
| 0 | Vitvi04g01109\_t001 |  |  |  |  |  |  |  |  |  |  |  |  |
| 0 | Vitvi04g01110\_t001 |  |  |  |  |  |  |  |  |  |  |  |  |
| 0 | Vitvi04g01111\_t001 |  |  |  |  |  |  |  |  |  |  |  |  |
| 0 | Vitvi04g01112\_t001 |  |  |  |  |  |  |  |  |  |  |  |  |
| 0 | Vitvi04g01113\_t001 |  |  |  |  |  |  |  |  |  |  |  |  |
| 0 | Vitvi04g04297\_t001 |  |  |  |  |  |  |  |  |  |  |  |  |
| 0 | Vitvi04g02053\_t001 |  |  |  |  |  |  |  |  |  |  |  |  |
| 0 | Vitvi04g01116\_t001 |  |  |  |  |  |  |  |  |  |  |  |  |
| 0 | Vitvi04g02054\_t001 |  |  |  |  |  |  |  |  |  |  |  |  |
| 0 | Vitvi04g01117\_t001 |  |  |  |  |  |  |  |  |  |  |  |  |
| 0 | Vitvi04g04298\_t001 |  |  |  |  |  |  |  |  |  |  |  |  |
| 0 | Vitvi04g04299\_t001 |  |  |  |  |  |  |  |  |  |  |  |  |
| 0 | Vitvi04g04300\_t001 |  |  |  |  |  |  |  |  |  |  |  |  |
| 0 | Vitvi04g04301\_t001 |  |  |  |  |  |  |  |  |  |  |  |  |
| 0 | Vitvi04g01121\_t001 |  |  |  |  |  |  |  |  |  |  |  |  |
| 0 | Vitvi04g01122\_t001 |  |  |  |  |  |  |  |  |  |  |  |  |
| 0 | Vitvi04g01124\_t001 |  |  |  |  |  |  |  |  |  |  |  |  |
| 0 | Vitvi04g01125\_t001 |  |  |  |  |  |  |  |  |  |  |  |  |
| 0 | Vitvi04g01126\_t002 |  |  |  |  |  |  |  |  |  |  |  |  |
| 0 | Vitvi04g01127\_t001 |  |  |  |  |  |  |  |  |  |  |  |  |
| 0 | Vitvi04g01128\_t001 |  |  |  |  |  |  |  |  |  |  |  |  |
| 0 | Vitvi04g01129\_t001 |  |  |  |  |  |  |  |  |  |  |  |  |
| 0 | Vitvi04g01130\_t001 |  |  |  |  |  |  |  |  |  |  |  |  |
| 0 | Vitvi04g01131\_t001 |  |  |  |  |  |  |  |  |  |  |  |  |
| 0 | Vitvi04g01133\_t001 |  |  |  |  |  |  |  |  |  |  |  |  |
| 0 | Vitvi04g01134\_t001 |  |  |  |  |  |  |  |  |  |  |  |  |
| 0 | Vitvi04g04302\_t001 |  |  |  |  |  |  |  |  |  |  |  |  |
| 0 | Vitvi04g01136\_t001 |  |  |  |  |  |  |  |  |  |  |  |  |
| 0 | Vitvi04g04303\_t001 |  |  |  |  |  |  |  |  |  |  |  |  |
| 0 | Vitvi04g04304\_t001 |  |  |  |  |  |  |  |  |  |  |  |  |
| 0 | Vitvi04g01138\_t001 |  |  |  |  |  |  |  |  |  |  |  |  |
| 0 | Vitvi04g01139\_t001 |  |  |  |  |  |  |  |  |  |  |  |  |
| 0 | Vitvi04g04305\_t001 |  |  |  |  |  |  |  |  |  |  |  |  |
| 0 | Vitvi04g01140\_t001 |  |  |  |  |  |  |  |  |  |  |  |  |
| 0 | Vitvi04g01141\_t001 |  |  |  |  |  |  |  |  |  |  |  |  |
| 0 | Vitvi04g01142\_t001 |  |  |  |  |  |  |  |  |  |  |  |  |
| 0 | Vitvi04g01143\_t001 |  |  |  |  |  |  |  |  |  |  |  |  |
| 0 | Vitvi04g01149\_t001 |  |  |  |  |  |  |  |  |  |  |  |  |
| 0 | Vitvi04g01151\_t001 |  |  |  |  |  |  |  |  |  |  |  |  |
| 0 | Vitvi04g01153\_t001 |  |  |  |  |  |  |  |  |  |  |  |  |
| 0 | Vitvi04g01155\_t001 |  |  |  |  |  |  |  |  |  |  |  |  |
| 0 | Vitvi04g01156\_t002 |  |  |  |  |  |  |  |  |  |  |  |  |
| 0 | Vitvi04g01157\_t002 |  |  |  |  |  |  |  |  |  |  |  |  |
| 0 | Vitvi04g01158\_t001 |  |  |  |  |  |  |  |  |  |  |  |  |
| 0 | Vitvi04g01159\_t001 |  |  |  |  |  |  |  |  |  |  |  |  |
| 0 | Vitvi04g02062\_t001 |  |  |  |  |  |  |  |  |  |  |  |  |
| 0 | Vitvi04g02063\_t001 |  |  |  |  |  |  |  |  |  |  |  |  |
| 0 | Vitvi04g01160\_t001 |  |  |  |  |  |  |  |  |  |  |  |  |
| 0 | Vitvi04g01161\_t001 |  |  |  |  |  |  |  |  |  |  |  |  |
| 0 | Vitvi04g01162\_t001 |  |  |  |  |  |  |  |  |  |  |  |  |
| 0 | Vitvi04g04306\_t001 |  |  |  |  |  |  |  |  |  |  |  |  |
| 0 | Vitvi04g01163\_t001 |  |  |  |  |  |  |  |  |  |  |  |  |
| 0 | Vitvi04g01164\_t001 |  |  |  |  |  |  |  |  |  |  |  |  |
| 0 | Vitvi04g04307\_t001 |  |  |  |  |  |  |  |  |  |  |  |  |
| 0 | Vitvi04g01165\_t001 |  |  |  |  |  |  |  |  |  |  |  |  |
| 0 | Vitvi04g01167\_t001 |  |  |  |  |  |  |  |  |  |  |  |  |
| 0 | Vitvi04g02064\_t001 |  |  |  |  |  |  |  |  |  |  |  |  |
| 0 | Vitvi04g02065\_t001 |  |  |  |  |  |  |  |  |  |  |  |  |
| 0 | Vitvi04g04308\_t001 |  |  |  |  |  |  |  |  |  |  |  |  |
| 0 | Vitvi04g04309\_t001 |  |  |  |  |  |  |  |  |  |  |  |  |
| 0 | Vitvi04g01168\_t001 |  |  |  |  |  |  |  |  |  |  |  |  |
| 0 | Vitvi04g04310\_t001 |  |  |  |  |  |  |  |  |  |  |  |  |
| 0 | Vitvi04g04311\_t001 |  |  |  |  |  |  |  |  |  |  |  |  |
| 0 | Vitvi04g01172\_t001 |  |  |  |  |  |  |  |  |  |  |  |  |
| 0 | Vitvi04g02068\_t001 |  |  |  |  |  |  |  |  |  |  |  |  |
| 0 | Vitvi04g04312\_t001 |  |  |  |  |  |  |  |  |  |  |  |  |
| 0 | Vitvi04g02069\_t001 |  |  |  |  |  |  |  |  |  |  |  |  |
| 0 | Vitvi04g02070\_t001 |  |  |  |  |  |  |  |  |  |  |  |  |
| 0 | Vitvi04g02071\_t001 |  |  |  |  |  |  |  |  |  |  |  |  |
| 0 | Vitvi04g02072\_t001 |  |  |  |  |  |  |  |  |  |  |  |  |
| 0 | Vitvi04g04313\_t001 |  |  |  |  |  |  |  |  |  |  |  |  |
| 0 | Vitvi04g02073\_t001 |  |  |  |  |  |  |  |  |  |  |  |  |
| 0 | Vitvi04g02074\_t001 |  |  |  |  |  |  |  |  |  |  |  |  |
| 0 | Vitvi04g02075\_t001 |  |  |  |  |  |  |  |  |  |  |  |  |
| 0 | Vitvi04g01174\_t001 |  |  |  |  |  |  |  |  |  |  |  |  |
| 0 | Vitvi04g01175\_t001 |  |  |  |  |  |  |  |  |  |  |  |  |
| 0 | Vitvi04g02076\_t001 |  |  |  |  |  |  |  |  |  |  |  |  |
| 0 | Vitvi04g01176\_t002 |  |  |  |  |  |  |  |  |  |  |  |  |
| 0 | Vitvi04g04314\_t001 |  |  |  |  |  |  |  |  |  |  |  |  |
| 0 | Vitvi04g04315\_t001 |  |  |  |  |  |  |  |  |  |  |  |  |
| 0 | Vitvi04g01180\_t001 |  |  |  |  |  |  |  |  |  |  |  |  |
| 0 | Vitvi04g01182\_t001 |  |  |  |  |  |  |  |  |  |  |  |  |
| 0 | Vitvi04g01183\_t001 |  |  |  |  |  |  |  |  |  |  |  |  |
| 0 | Vitvi04g01184\_t002 |  |  |  |  |  |  |  |  |  |  |  |  |
| 0 | Vitvi04g01185\_t001 |  |  |  |  |  |  |  |  |  |  |  |  |
| 0 | Vitvi04g01186\_t001 |  |  |  |  |  |  |  |  |  |  |  |  |
| 0 | Vitvi04g04316\_t001 |  |  |  |  |  |  |  |  |  |  |  |  |
| 0 | Vitvi04g04317\_t001 |  |  |  |  |  |  |  |  |  |  |  |  |
| 0 | Vitvi04g04318\_t001 |  |  |  |  |  |  |  |  |  |  |  |  |
| 0 | Vitvi04g01189\_t002 |  |  |  |  |  |  |  |  |  |  |  |  |
| 0 | Vitvi04g01190\_t001 |  |  |  |  |  |  |  |  |  |  |  |  |
| 0 | Vitvi04g04319\_t001 |  |  |  |  |  |  |  |  |  |  |  |  |
| 0 | Vitvi04g01192\_t001 |  |  |  |  |  |  |  |  |  |  |  |  |
| 0 | Vitvi04g01193\_t002 |  |  |  |  |  |  |  |  |  |  |  |  |
| 0 | Vitvi04g01194\_t001 |  |  |  |  |  |  |  |  |  |  |  |  |
| 0 | Vitvi04g04320\_t001 |  |  |  |  |  |  |  |  |  |  |  |  |
| 0 | Vitvi04g01195\_t001 |  |  |  |  |  |  |  |  |  |  |  |  |
| 0 | Vitvi04g01196\_t001 |  |  |  |  |  |  |  |  |  |  |  |  |
| 0 | Vitvi04g01197\_t001 |  |  |  |  |  |  |  |  |  |  |  |  |
| 0 | Vitvi04g02083\_t001 |  |  |  |  |  |  |  |  |  |  |  |  |
| 0 | Vitvi04g01198\_t001 |  |  |  |  |  |  |  |  |  |  |  |  |
| 0 | Vitvi04g01199\_t001 |  |  |  |  |  |  |  |  |  |  |  |  |
| 0 | Vitvi04g02084\_t001 |  |  |  |  |  |  |  |  |  |  |  |  |
| 0 | Vitvi04g01200\_t001 |  |  |  |  |  |  |  |  |  |  |  |  |
| 0 | Vitvi04g01201\_t001 |  |  |  |  |  |  |  |  |  |  |  |  |
| 0 | Vitvi04g04321\_t001 |  |  |  |  |  |  |  |  |  |  |  |  |
| 0 | Vitvi04g04322\_t001 |  |  |  |  |  |  |  |  |  |  |  |  |
| 0 | Vitvi04g04323\_t001 |  |  |  |  |  |  |  |  |  |  |  |  |
| 0 | Vitvi04g04324\_t001 |  |  |  |  |  |  |  |  |  |  |  |  |
| 0 | Vitvi04g01202\_t001 |  |  |  |  |  |  |  |  |  |  |  |  |
| 0 | Vitvi04g01205\_t001 |  |  |  |  |  |  |  |  |  |  |  |  |
| 0 | Vitvi04g04325\_t001 |  |  |  |  |  |  |  |  |  |  |  |  |
| 0 | Vitvi04g01206\_t001 |  |  |  |  |  |  |  |  |  |  |  |  |
| 0 | Vitvi04g01207\_t001 |  |  |  |  |  |  |  |  |  |  |  |  |
| 0 | Vitvi04g02087\_t001 |  |  |  |  |  |  |  |  |  |  |  |  |
| 0 | Vitvi04g04326\_t001 |  |  |  |  |  |  |  |  |  |  |  |  |
| 0 | Vitvi04g01208\_t002 |  |  |  |  |  |  |  |  |  |  |  |  |
| 0 | Vitvi04g02090\_t001 |  |  |  |  |  |  |  |  |  |  |  |  |
| 0 | Vitvi04g01209\_t001 |  |  |  |  |  |  |  |  |  |  |  |  |
| 1 | Vitvi04g01210\_t001 |  | Vitvi18g00902\_t001 |  |  |  |  |  |  |  |  |  |  |
| 1 | Vitvi04g04327\_t001 |  | \| \| |  |  |  |  |  |  |  |  |  |  |
| 1 | Vitvi04g02091\_t001 |  | Vitvi18g00903\_t001 |  |  |  |  |  |  |  |  |  |  |
| 1 | Vitvi04g02092\_t001 |  | \| \| |  |  |  |  |  |  |  |  |  |  |
| 1 | Vitvi04g01212\_t001 |  | \| \| |  |  |  |  |  |  |  |  |  |  |
| 1 | Vitvi04g01214\_t001 |  | \| \| |  |  |  |  |  |  |  |  |  |  |
| 1 | Vitvi04g01216\_t001 |  | Vitvi18g00904\_t001 |  |  |  |  |  |  |  |  |  |  |
| 1 | Vitvi04g01218\_t001 |  | \| \| |  |  |  |  |  |  |  |  |  |  |
| 2 | Vitvi04g02094\_t001 |  | \| \| |  | Vitvi03g00389\_t001 |  |  |  |  |  |  |  |  |
| 2 | Vitvi04g01220\_t001 |  | Vitvi18g00905\_t001 |  | \| \| |  |  |  |  |  |  |  |  |
| 2 | Vitvi04g04328\_t001 |  | \| \| |  | \| \| |  |  |  |  |  |  |  |  |
| 2 | Vitvi04g01221\_t002 |  | \| \| |  | \| \| |  |  |  |  |  |  |  |  |
| 2 | Vitvi04g01222\_t001 |  | Vitvi18g00909\_t001 |  | \| \| |  |  |  |  |  |  |  |  |
| 2 | Vitvi04g01223\_t001 |  | Vitvi18g00912\_t001 |  | Vitvi03g00372\_t001 |  |  |  |  |  |  |  |  |
| 2 | Vitvi04g04329\_t001 |  | \| \| |  | \| \| |  |  |  |  |  |  |  |  |
| 2 | Vitvi04g01224\_t001 |  | Vitvi18g00913\_t001 |  | \| \| |  |  |  |  |  |  |  |  |
| 2 | Vitvi04g01225\_t001 |  | Vitvi18g02730\_t001 |  | \| \| |  |  |  |  |  |  |  |  |
| 2 | Vitvi04g01226\_t001 |  | \| \| |  | \| \| |  |  |  |  |  |  |  |  |
| 2 | Vitvi04g04330\_t001 |  | \| \| |  | \| \| |  |  |  |  |  |  |  |  |
| 2 | Vitvi04g01227\_t001 |  | Vitvi18g00919\_t001 |  | \| \| |  |  |  |  |  |  |  |  |
| 2 | Vitvi04g01228\_t001 |  | \| \| |  | Vitvi03g00369\_t001 |  |  |  |  |  |  |  |  |
| 2 | Vitvi04g04331\_t001 |  | \| \| |  | \| \| |  |  |  |  |  |  |  |  |
| 2 | Vitvi04g02095\_t001 |  | \| \| |  | \| \| |  |  |  |  |  |  |  |  |
| 2 | Vitvi04g01230\_t001 |  | Vitvi18g00922\_t001 |  | \| \| |  |  |  |  |  |  |  |  |
| 2 | Vitvi04g01231\_t001 |  | \| \| |  | Vitvi03g00368\_t001 |  |  |  |  |  |  |  |  |
| 2 | Vitvi04g01232\_t001 |  | \| \| |  | \| \| |  |  |  |  |  |  |  |  |
| 2 | Vitvi04g01233\_t001 |  | \| \| |  | \| \| |  |  |  |  |  |  |  |  |
| 2 | Vitvi04g04332\_t001 |  | \| \| |  | \| \| |  |  |  |  |  |  |  |  |
| 2 | Vitvi04g01234\_t001 |  | Vitvi18g00924\_t001 |  | \| \| |  |  |  |  |  |  |  |  |
| 2 | Vitvi04g02096\_t001 |  | \| \| |  | \| \| |  |  |  |  |  |  |  |  |
| 2 | Vitvi04g01235\_t001 |  | \| \| |  | \| \| |  |  |  |  |  |  |  |  |
| 2 | Vitvi04g01236\_t003 |  | \| \| |  | Vitvi03g04128\_t001 |  |  |  |  |  |  |  |  |
| 2 | Vitvi04g01237\_t001 |  | Vitvi18g04218\_t001 |  | \| \| |  |  |  |  |  |  |  |  |
| 2 | Vitvi04g01238\_t001 |  | \| \| |  | \| \| |  |  |  |  |  |  |  |  |
| 2 | Vitvi04g04333\_t001 |  | \| \| |  | \| \| |  |  |  |  |  |  |  |  |
| 2 | Vitvi04g04334\_t001 |  | \| \| |  | \| \| |  |  |  |  |  |  |  |  |
| 2 | Vitvi04g01242\_t001 |  | \| \| |  | \| \| |  |  |  |  |  |  |  |  |
| 2 | Vitvi04g01243\_t001 |  | \| \| |  | Vitvi03g00365\_t001 |  |  |  |  |  |  |  |  |
| 2 | Vitvi04g01244\_t001 |  | \| \| |  | \| \| |  |  |  |  |  |  |  |  |
| 2 | Vitvi04g01246\_t001 |  | \| \| |  | \| \| |  |  |  |  |  |  |  |  |
| 2 | Vitvi04g04335\_t001 |  | \| \| |  | \| \| |  |  |  |  |  |  |  |  |
| 2 | Vitvi04g02098\_t001 |  | Vitvi18g00930\_t003 |  | Vitvi03g04127\_t001 |  |  |  |  |  |  |  |  |
| 2 | Vitvi04g02099\_t001 |  | \| \| |  | Vitvi03g01512\_t001 |  |  |  |  |  |  |  |  |
| 2 | Vitvi04g01247\_t001 |  | \| \| |  | \| \| |  |  |  |  |  |  |  |  |
| 2 | Vitvi04g01248\_t001 |  | \| \| |  | \| \| |  |  |  |  |  |  |  |  |
| 2 | Vitvi04g01249\_t001 |  | Vitvi18g00933\_t005 |  | \| \| |  |  |  |  |  |  |  |  |
| 2 | Vitvi04g04336\_t001 |  | \| \| |  | \| \| |  |  |  |  |  |  |  |  |
| 2 | Vitvi04g01251\_t001 |  | \| \| |  | \| \| |  |  |  |  |  |  |  |  |
| 2 | Vitvi04g04337\_t001 |  | \| \| |  | \| \| |  |  |  |  |  |  |  |  |
| 2 | Vitvi04g01252\_t001 |  | Vitvi18g00935\_t001 |  | \| \| |  |  |  |  |  |  |  |  |
| 2 | Vitvi04g04338\_t001 |  | \| \| |  | \| \| |  |  |  |  |  |  |  |  |
| 2 | Vitvi04g01254\_t001 |  | \| \| |  | \| \| |  |  |  |  |  |  |  |  |
| 2 | Vitvi04g01255\_t001 |  | \| \| |  | \| \| |  |  |  |  |  |  |  |  |
| 2 | Vitvi04g01256\_t002 |  | \| \| |  | \| \| |  |  |  |  |  |  |  |  |
| 2 | Vitvi04g01257\_t001 |  | \| \| |  | \| \| |  |  |  |  |  |  |  |  |
| 2 | Vitvi04g01258\_t001 |  | \| \| |  | \| \| |  |  |  |  |  |  |  |  |
| 2 | Vitvi04g01260\_t001 |  | \| \| |  | \| \| |  |  |  |  |  |  |  |  |
| 2 | Vitvi04g01261\_t001 |  | \| \| |  | \| \| |  |  |  |  |  |  |  |  |
| 2 | Vitvi04g01262\_t001 |  | Vitvi18g00946\_t001 |  | \| \| |  |  |  |  |  |  |  |  |
| 2 | Vitvi04g01263\_t001 |  | \| \| |  | Vitvi03g00359\_t001 |  |  |  |  |  |  |  |  |
| 2 | Vitvi04g01264\_t001 |  | Vitvi18g00947\_t001 |  | \| \| |  |  |  |  |  |  |  |  |
| 2 | Vitvi04g01265\_t001 |  | Vitvi18g00948\_t001 |  | \| \| |  |  |  |  |  |  |  |  |
| 2 | Vitvi04g01266\_t001 |  | \| \| |  | \| \| |  |  |  |  |  |  |  |  |
| 2 | Vitvi04g02101\_t001 |  | \| \| |  | Vitvi03g00355\_t001 |  |  |  |  |  |  |  |  |
| 2 | Vitvi04g01267\_t001 |  | \| \| |  | \| \| |  |  |  |  |  |  |  |  |
| 2 | Vitvi04g01268\_t001 |  | \| \| |  | \| \| |  |  |  |  |  |  |  |  |
| 2 | Vitvi04g01269\_t001 |  | \| \| |  | Vitvi03g00345\_t001 |  |  |  |  |  |  |  |  |
| 2 | Vitvi04g01271\_t001 |  | \| \| |  | \| \| |  |  |  |  |  |  |  |  |
| 2 | Vitvi04g01274\_t001 |  | \| \| |  | \| \| |  |  |  |  |  |  |  |  |
| 2 | Vitvi04g02103\_t001 |  | \| \| |  | \| \| |  |  |  |  |  |  |  |  |
| 2 | Vitvi04g01275\_t001 |  | \| \| |  | \| \| |  |  |  |  |  |  |  |  |
| 2 | Vitvi04g01276\_t003 |  | \| \| |  | \| \| |  |  |  |  |  |  |  |  |
| 2 | Vitvi04g01278\_t001 |  | \| \| |  | \| \| |  |  |  |  |  |  |  |  |
| 2 | Vitvi04g01280\_t001 |  | \| \| |  | \| \| |  |  |  |  |  |  |  |  |
| 2 | Vitvi04g01281\_t001 |  | \| \| |  | \| \| |  |  |  |  |  |  |  |  |
| 2 | Vitvi04g04339\_t001 |  | \| \| |  | \| \| |  |  |  |  |  |  |  |  |
| 2 | Vitvi04g01282\_t001 |  | \| \| |  | \| \| |  |  |  |  |  |  |  |  |
| 2 | Vitvi04g04340\_t001 |  | \| \| |  | \| \| |  |  |  |  |  |  |  |  |
| 2 | Vitvi04g04341\_t001 |  | \| \| |  | \| \| |  |  |  |  |  |  |  |  |
| 2 | Vitvi04g04342\_t001 |  | \| \| |  | \| \| |  |  |  |  |  |  |  |  |
| 2 | Vitvi04g01287\_t001 |  | Vitvi18g00957\_t003 |  | \| \| |  |  |  |  |  |  |  |  |
| 2 | Vitvi04g04343\_t001 |  | \| \| |  | \| \| |  |  |  |  |  |  |  |  |
| 2 | Vitvi04g01289\_t001 |  | \| \| |  | \| \| |  |  |  |  |  |  |  |  |
| 2 | Vitvi04g01290\_t001 |  | \| \| |  | \| \| |  |  |  |  |  |  |  |  |
| 2 | Vitvi04g01291\_t001 |  | \| \| |  | \| \| |  |  |  |  |  |  |  |  |
| 2 | Vitvi04g01292\_t001 |  | \| \| |  | \| \| |  |  |  |  |  |  |  |  |
| 2 | Vitvi04g01293\_t001 |  | \| \| |  | \| \| |  |  |  |  |  |  |  |  |
| 2 | Vitvi04g01296\_t001 |  | Vitvi18g00968\_t002 |  | \| \| |  |  |  |  |  |  |  |  |
| 2 | Vitvi04g04344\_t001 |  | \| \| |  | \| \| |  |  |  |  |  |  |  |  |
| 2 | Vitvi04g01297\_t002 |  | \| \| |  | \| \| |  |  |  |  |  |  |  |  |
| 2 | Vitvi04g01298\_t001 |  | \| \| |  | \| \| |  |  |  |  |  |  |  |  |
| 2 | Vitvi04g01299\_t001 |  | \| \| |  | \| \| |  |  |  |  |  |  |  |  |
| 2 | Vitvi04g01300\_t001 |  | \| \| |  | Vitvi03g00321\_t001 |  |  |  |  |  |  |  |  |
| 2 | Vitvi04g01302\_t001 |  | Vitvi18g00970\_t002 |  | \| \| |  |  |  |  |  |  |  |  |
| 2 | Vitvi04g01303\_t001 |  | Vitvi18g00971\_t001 |  | Vitvi03g00319\_t001 |  |  |  |  |  |  |  |  |
| 2 | Vitvi04g01304\_t001 |  | Vitvi18g00972\_t001 |  | \| \| |  |  |  |  |  |  |  |  |
| 2 | Vitvi04g01306\_t001 |  | \| \| |  | \| \| |  |  |  |  |  |  |  |  |
| 2 | Vitvi04g01307\_t001 |  | \| \| |  | \| \| |  |  |  |  |  |  |  |  |
| 2 | Vitvi04g01309\_t001 |  | Vitvi18g00973\_t001 |  | Vitvi03g01495\_t001 |  |  |  |  |  |  |  |  |
| 2 | Vitvi04g04345\_t001 |  | \| \| |  | \| \| |  |  |  |  |  |  |  |  |
| 2 | Vitvi04g01311\_t001 |  | Vitvi18g00974\_t001 |  | \| \| |  |  |  |  |  |  |  |  |
| 2 | Vitvi04g01312\_t001 |  | \| \| |  | Vitvi03g00315\_t001 |  |  |  |  |  |  |  |  |
| 2 | Vitvi04g01313\_t001 |  | \| \| |  | \| \| |  |  |  |  |  |  |  |  |
| 2 | Vitvi04g01315\_t001 |  | Vitvi18g00976\_t001 |  | \| \| |  |  |  |  |  |  |  |  |
| 2 | Vitvi04g04346\_t001 |  | \| \| |  | \| \| |  |  |  |  |  |  |  |  |
| 2 | Vitvi04g01317\_t001 |  | \| \| |  | \| \| |  |  |  |  |  |  |  |  |
| 2 | Vitvi04g01318\_t001 |  | \| \| |  | \| \| |  |  |  |  |  |  |  |  |
| 2 | Vitvi04g01319\_t001 |  | \| \| |  | Vitvi03g00311\_t001 |  |  |  |  |  |  |  |  |
| 2 | Vitvi04g01320\_t003 |  | \| \| |  | \| \| |  |  |  |  |  |  |  |  |
| 2 | Vitvi04g01321\_t001 |  | \| \| |  | \| \| |  |  |  |  |  |  |  |  |
| 2 | Vitvi04g01322\_t001 |  | \| \| |  | \| \| |  |  |  |  |  |  |  |  |
| 2 | Vitvi04g02109\_t001 |  | \| \| |  | \| \| |  |  |  |  |  |  |  |  |
| 2 | Vitvi04g01323\_t002 |  | Vitvi18g00980\_t001 |  | Vitvi03g00307\_t003 |  |  |  |  |  |  |  |  |
| 2 | Vitvi04g01324\_t002 |  | \| \| |  | \| \| |  |  |  |  |  |  |  |  |
| 2 | Vitvi04g01325\_t001 |  | Vitvi18g00981\_t001 |  | \| \| |  |  |  |  |  |  |  |  |
| 2 | Vitvi04g01327\_t002 |  | Vitvi18g00983\_t001 |  | \| \| |  |  |  |  |  |  |  |  |
| 2 | Vitvi04g04347\_t001 |  | Vitvi18g04232\_t001 |  | \| \| |  |  |  |  |  |  |  |  |
| 2 | Vitvi04g04348\_t001 |  | \| \| |  | \| \| |  |  |  |  |  |  |  |  |
| 2 | Vitvi04g01330\_t001 |  | \| \| |  | \| \| |  |  |  |  |  |  |  |  |
| 2 | Vitvi04g01332\_t001 |  | \| \| |  | \| \| |  |  |  |  |  |  |  |  |
| 2 | Vitvi04g04349\_t001 |  | \| \| |  | \| \| |  |  |  |  |  |  |  |  |
| 2 | Vitvi04g01334\_t001 |  | Vitvi18g00991\_t001 |  | \| \| |  |  |  |  |  |  |  |  |
| 2 | Vitvi04g01335\_t001 |  | \| \| |  | \| \| |  |  |  |  |  |  |  |  |
| 2 | Vitvi04g04350\_t001 |  | \| \| |  | \| \| |  |  |  |  |  |  |  |  |
| 2 | Vitvi04g01336\_t001 |  | \| \| |  | \| \| |  |  |  |  |  |  |  |  |
| 2 | Vitvi04g01337\_t001 |  | Vitvi18g00992\_t001 |  | Vitvi03g00302\_t001 |  |  |  |  |  |  |  |  |
| 2 | Vitvi04g01338\_t001 |  | \| \| |  | \| \| |  |  |  |  |  |  |  |  |
| 2 | Vitvi04g04351\_t001 |  | \| \| |  | \| \| |  |  |  |  |  |  |  |  |
| 2 | Vitvi04g02111\_t001 |  | \| \| |  | \| \| |  |  |  |  |  |  |  |  |
| 2 | Vitvi04g04352\_t001 |  | \| \| |  | \| \| |  |  |  |  |  |  |  |  |
| 2 | Vitvi04g01339\_t001 |  | \| \| |  | \| \| |  |  |  |  |  |  |  |  |
| 2 | Vitvi04g04353\_t001 |  | \| \| |  | \| \| |  |  |  |  |  |  |  |  |
| 2 | Vitvi04g02114\_t001 |  | \| \| |  | \| \| |  |  |  |  |  |  |  |  |
| 2 | Vitvi04g02116\_t001 |  | \| \| |  | Vitvi03g00301\_t001 |  |  |  |  |  |  |  |  |
| 2 | Vitvi04g02117\_t001 |  | \| \| |  | Vitvi03g00299\_t001 |  |  |  |  |  |  |  |  |
| 2 | Vitvi04g01342\_t001 |  | \| \| |  | \| \| |  |  |  |  |  |  |  |  |
| 2 | Vitvi04g04354\_t001 |  | \| \| |  | \| \| |  |  |  |  |  |  |  |  |
| 2 | Vitvi04g04355\_t001 |  | \| \| |  | \| \| |  |  |  |  |  |  |  |  |
| 2 | Vitvi04g02122\_t001 |  | \| \| |  | \| \| |  |  |  |  |  |  |  |  |
| 2 | Vitvi04g02123\_t001 |  | Vitvi18g02761\_t002 |  | \| \| |  |  |  |  |  |  |  |  |
| 2 | Vitvi04g04356\_t001 |  | \| \| |  | \| \| |  |  |  |  |  |  |  |  |
| 2 | Vitvi04g04357\_t001 |  | \| \| |  | \| \| |  |  |  |  |  |  |  |  |
| 2 | Vitvi04g02125\_t001 |  | \| \| |  | \| \| |  |  |  |  |  |  |  |  |
| 2 | Vitvi04g04358\_t001 |  | \| \| |  | \| \| |  |  |  |  |  |  |  |  |
| 2 | Vitvi04g04359\_t001 |  | \| \| |  | \| \| |  |  |  |  |  |  |  |  |
| 2 | Vitvi04g01345\_t001 |  | \| \| |  | \| \| |  |  |  |  |  |  |  |  |
| 2 | Vitvi04g04360\_t001 |  | \| \| |  | \| \| |  |  |  |  |  |  |  |  |
| 2 | Vitvi04g04361\_t001 |  | Vitvi18g00995\_t001 |  | \| \| |  |  |  |  |  |  |  |  |
| 2 | Vitvi04g04362\_t001 |  | \| \| |  | \| \| |  |  |  |  |  |  |  |  |
| 2 | Vitvi04g04363\_t001 |  | \| \| |  | \| \| |  |  |  |  |  |  |  |  |
| 2 | Vitvi04g01349\_t001 |  | \| \| |  | \| \| |  |  |  |  |  |  |  |  |
| 2 | Vitvi04g01350\_t001 |  | \| \| |  | \| \| |  |  |  |  |  |  |  |  |
| 2 | Vitvi04g02127\_t001 |  | \| \| |  | \| \| |  |  |  |  |  |  |  |  |
| 2 | Vitvi04g01351\_t001 |  | \| \| |  | \| \| |  |  |  |  |  |  |  |  |
| 2 | Vitvi04g01352\_t001 |  | \| \| |  | \| \| |  |  |  |  |  |  |  |  |
| 2 | Vitvi04g04364\_t001 |  | \| \| |  | \| \| |  |  |  |  |  |  |  |  |
| 2 | Vitvi04g01353\_t001 |  | \| \| |  | Vitvi03g00298\_t001 |  |  |  |  |  |  |  |  |
| 2 | Vitvi04g01356\_t001 |  | \| \| |  | \| \| |  |  |  |  |  |  |  |  |
| 2 | Vitvi04g01357\_t001 |  | \| \| |  | \| \| |  |  |  |  |  |  |  |  |
| 2 | Vitvi04g01358\_t001 |  | \| \| |  | \| \| |  |  |  |  |  |  |  |  |
| 2 | Vitvi04g01359\_t001 |  | \| \| |  | \| \| |  |  |  |  |  |  |  |  |
| 2 | Vitvi04g01360\_t001 |  | Vitvi18g00997\_t001 |  | Vitvi03g00294\_t001 |  |  |  |  |  |  |  |  |
| 2 | Vitvi04g01361\_t001 |  | Vitvi18g00999\_t001 |  | \| \| |  |  |  |  |  |  |  |  |
| 2 | Vitvi04g01362\_t001 |  | Vitvi18g01004\_t001 |  | \| \| |  |  |  |  |  |  |  |  |
| 2 | Vitvi04g01363\_t001 |  | Vitvi18g01005\_t001 |  | Vitvi03g00292\_t001 |  |  |  |  |  |  |  |  |
| 2 | Vitvi04g01364\_t001 |  | \| \| |  | \| \| |  |  |  |  |  |  |  |  |
| 2 | Vitvi04g01366\_t001 |  | \| \| |  | \| \| |  |  |  |  |  |  |  |  |
| 2 | Vitvi04g01367\_t002 |  | \| \| |  | \| \| |  |  |  |  |  |  |  |  |
| 2 | Vitvi04g04365\_t001 |  | \| \| |  | \| \| |  |  |  |  |  |  |  |  |
| 2 | Vitvi04g01368\_t001 |  | \| \| |  | Vitvi03g01481\_t001 |  |  |  |  |  |  |  |  |
| 1 | Vitvi04g01369\_t001 |  | \| \| |  |  |  |  |  |  |  |  |  |  |
| 1 | Vitvi04g01370\_t001 |  | \| \| |  |  |  |  |  |  |  |  |  |  |
| 1 | Vitvi04g01371\_t001 |  | Vitvi18g01013\_t002 |  |  |  |  |  |  |  |  |  |  |
| 1 | Vitvi04g04366\_t001 |  | \| \| |  |  |  |  |  |  |  |  |  |  |
| 1 | Vitvi04g01372\_t001 |  | \| \| |  |  |  |  |  |  |  |  |  |  |
| 1 | Vitvi04g04367\_t001 |  | \| \| |  |  |  |  |  |  |  |  |  |  |
| 1 | Vitvi04g04368\_t001 |  | \| \| |  |  |  |  |  |  |  |  |  |  |
| 1 | Vitvi04g04369\_t001 |  | \| \| |  |  |  |  |  |  |  |  |  |  |
| 1 | Vitvi04g01375\_t001 |  | \| \| |  |  |  |  |  |  |  |  |  |  |
| 1 | Vitvi04g04370\_t001 |  | \| \| |  |  |  |  |  |  |  |  |  |  |
| 1 | Vitvi04g01377\_t002 |  | \| \| |  |  |  |  |  |  |  |  |  |  |
| 1 | Vitvi04g01378\_t001 |  | Vitvi18g01014\_t001 |  |  |  |  |  |  |  |  |  |  |
| 1 | Vitvi04g01379\_t001 |  | \| \| |  |  |  |  |  |  |  |  |  |  |
| 1 | Vitvi04g02132\_t001 |  | \| \| |  |  |  |  |  |  |  |  |  |  |
| 1 | Vitvi04g01381\_t002 |  | \| \| |  |  |  |  |  |  |  |  |  |  |
| 2 | Vitvi04g01382\_t001 |  | \| \| |  | Vitvi03g04008\_t001 |  |  |  |  |  |  |  |  |
| 2 | Vitvi04g02133\_t001 |  | Vitvi18g04240\_t001 |  | \| \| |  |  |  |  |  |  |  |  |
| 2 | Vitvi04g01383\_t001 |  | \| \| |  | \| \| |  |  |  |  |  |  |  |  |
| 2 | Vitvi04g01384\_t001 |  | \| \| |  | \| \| |  |  |  |  |  |  |  |  |
| 2 | Vitvi04g01385\_t001 |  | \| \| |  | \| \| |  |  |  |  |  |  |  |  |
| 2 | Vitvi04g01386\_t001 |  | \| \| |  | \| \| |  |  |  |  |  |  |  |  |
| 2 | Vitvi04g04371\_t001 |  | \| \| |  | \| \| |  |  |  |  |  |  |  |  |
| 2 | Vitvi04g01388\_t001 |  | \| \| |  | \| \| |  |  |  |  |  |  |  |  |
| 2 | Vitvi04g01389\_t001 |  | Vitvi18g01022\_t002 |  | Vitvi03g00009\_t001 |  |  |  |  |  |  |  |  |
| 2 | Vitvi04g01390\_t001 |  | Vitvi18g02775\_t001 |  | \| \| |  |  |  |  |  |  |  |  |
| 2 | Vitvi04g01391\_t003 |  | \| \| |  | \| \| |  |  |  |  |  |  |  |  |
| 2 | Vitvi04g01392\_t001 |  | Vitvi18g01034\_t001 |  | \| \| |  |  |  |  |  |  |  |  |
| 2 | Vitvi04g01393\_t001 |  | \| \| |  | \| \| |  |  |  |  |  |  |  |  |
| 2 | Vitvi04g01396\_t001 |  | \| \| |  | \| \| |  |  |  |  |  |  |  |  |
| 2 | Vitvi04g01397\_t001 |  | \| \| |  | \| \| |  |  |  |  |  |  |  |  |
| 2 | Vitvi04g04372\_t001 |  | \| \| |  | \| \| |  |  |  |  |  |  |  |  |
| 2 | Vitvi04g01399\_t001 |  | Vitvi18g01041\_t001 |  | Vitvi03g00024\_t001 |  |  |  |  |  |  |  |  |
| 2 | Vitvi04g04373\_t001 |  | \| \| |  | \| \| |  |  |  |  |  |  |  |  |
| 2 | Vitvi04g01400\_t001 |  | \| \| |  | \| \| |  |  |  |  |  |  |  |  |
| 2 | Vitvi04g01401\_t001 |  | \| \| |  | \| \| |  |  |  |  |  |  |  |  |
| 2 | Vitvi04g02136\_t001 |  | \| \| |  | \| \| |  |  |  |  |  |  |  |  |
| 2 | Vitvi04g01402\_t001 |  | \| \| |  | \| \| |  |  |  |  |  |  |  |  |
| 2 | Vitvi04g04374\_t001 |  | \| \| |  | \| \| |  |  |  |  |  |  |  |  |
| 2 | Vitvi04g01403\_t001 |  | \| \| |  | \| \| |  |  |  |  |  |  |  |  |
| 2 | Vitvi04g04375\_t001 |  | \| \| |  | \| \| |  |  |  |  |  |  |  |  |
| 2 | Vitvi04g01404\_t001 |  | Vitvi18g01044\_t001 |  | \| \| |  |  |  |  |  |  |  |  |
| 2 | Vitvi04g01406\_t001 |  | Vitvi18g01045\_t001 |  | \| \| |  |  |  |  |  |  |  |  |
| 2 | Vitvi04g01407\_t001 |  | Vitvi18g01047\_t001 |  | \| \| |  |  |  |  |  |  |  |  |
| 2 | Vitvi04g02137\_t001 |  | \| \| |  | \| \| |  |  |  |  |  |  |  |  |
| 2 | Vitvi04g02138\_t001 |  | \| \| |  | \| \| |  |  |  |  |  |  |  |  |
| 2 | Vitvi04g01408\_t001 |  | \| \| |  | \| \| |  |  |  |  |  |  |  |  |
| 2 | Vitvi04g04376\_t001 |  | \| \| |  | \| \| |  |  |  |  |  |  |  |  |
| 2 | Vitvi04g01410\_t001 |  | \| \| |  | Vitvi03g00037\_t001 |  |  |  |  |  |  |  |  |
| 2 | Vitvi04g04377\_t001 |  | \| \| |  | \| \| |  |  |  |  |  |  |  |  |
| 2 | Vitvi04g01412\_t001 |  | \| \| |  | Vitvi03g00038\_t001 |  |  |  |  |  |  |  |  |
| 2 | Vitvi04g02143\_t002 |  | \| \| |  | Vitvi03g01326\_t001 |  |  |  |  |  |  |  |  |
| 2 | Vitvi04g02144\_t001 |  | \| \| |  | \| \| |  |  |  |  |  |  |  |  |
| 2 | Vitvi04g01413\_t001 |  | \| \| |  | \| \| |  |  |  |  |  |  |  |  |
| 2 | Vitvi04g01414\_t002 |  | \| \| |  | Vitvi03g00042\_t001 |  |  |  |  |  |  |  |  |
| 2 | Vitvi04g04378\_t001 |  | \| \| |  | \| \| |  |  |  |  |  |  |  |  |
| 2 | Vitvi04g04379\_t001 |  | \| \| |  | \| \| |  |  |  |  |  |  |  |  |
| 2 | Vitvi04g01416\_t001 |  | \| \| |  | \| \| |  |  |  |  |  |  |  |  |
| 2 | Vitvi04g01417\_t001 |  | \| \| |  | \| \| |  |  |  |  |  |  |  |  |
| 2 | Vitvi04g01418\_t001 |  | \| \| |  | \| \| |  |  |  |  |  |  |  |  |
| 2 | Vitvi04g01419\_t001 |  | \| \| |  | \| \| |  |  |  |  |  |  |  |  |
| 2 | Vitvi04g04380\_t001 |  | \| \| |  | \| \| |  |  |  |  |  |  |  |  |
| 2 | Vitvi04g01420\_t001 |  | \| \| |  | \| \| |  |  |  |  |  |  |  |  |
| 2 | Vitvi04g01421\_t001 |  | \| \| |  | Vitvi03g00048\_t001 |  |  |  |  |  |  |  |  |
| 2 | Vitvi04g01422\_t002 |  | \| \| |  | \| \| |  |  |  |  |  |  |  |  |
| 2 | Vitvi04g04381\_t001 |  | \| \| |  | \| \| |  |  |  |  |  |  |  |  |
| 2 | Vitvi04g01423\_t003 |  | Vitvi18g01048\_t001 |  | Vitvi03g00049\_t001 |  |  |  |  |  |  |  |  |
| 2 | Vitvi04g01424\_t001 |  | \| \| |  | \| \| |  |  |  |  |  |  |  |  |
| 2 | Vitvi04g01425\_t003 |  | Vitvi18g01055\_t001 |  | Vitvi03g00050\_t001 |  |  |  |  |  |  |  |  |
| 2 | Vitvi04g04382\_t001 |  | \| \| |  | \| \| |  |  |  |  |  |  |  |  |
| 2 | Vitvi04g01426\_t001 |  | Vitvi18g01056\_t001 |  | \| \| |  |  |  |  |  |  |  |  |
| 2 | Vitvi04g01429\_t001 |  | \| \| |  | Vitvi03g00057\_t001 |  |  |  |  |  |  |  |  |
| 2 | Vitvi04g04383\_t001 |  | \| \| |  | \| \| |  |  |  |  |  |  |  |  |
| 2 | Vitvi04g01430\_t001 |  | \| \| |  | \| \| |  |  |  |  |  |  |  |  |
| 2 | Vitvi04g04384\_t001 |  | Vitvi18g04248\_t001 |  | \| \| |  |  |  |  |  |  |  |  |
| 2 | Vitvi04g01432\_t001 |  | \| \| |  | \| \| |  |  |  |  |  |  |  |  |
| 2 | Vitvi04g04385\_t001 |  | \| \| |  | \| \| |  |  |  |  |  |  |  |  |
| 2 | Vitvi04g01433\_t001 |  | \| \| |  | \| \| |  |  |  |  |  |  |  |  |
| 2 | Vitvi04g01434\_t001 |  | \| \| |  | \| \| |  |  |  |  |  |  |  |  |
| 2 | Vitvi04g02147\_t001 |  | \| \| |  | \| \| |  |  |  |  |  |  |  |  |
| 2 | Vitvi04g01435\_t001 |  | \| \| |  | \| \| |  |  |  |  |  |  |  |  |
| 2 | Vitvi04g01436\_t001 |  | Vitvi18g01081\_t001 |  | \| \| |  |  |  |  |  |  |  |  |
| 2 | Vitvi04g01437\_t001 |  | \| \| |  | \| \| |  |  |  |  |  |  |  |  |
| 2 | Vitvi04g01438\_t001 |  | \| \| |  | \| \| |  |  |  |  |  |  |  |  |
| 2 | Vitvi04g01439\_t001 |  | \| \| |  | \| \| |  |  |  |  |  |  |  |  |
| 2 | Vitvi04g04386\_t001 |  | \| \| |  | \| \| |  |  |  |  |  |  |  |  |
| 2 | Vitvi04g04387\_t001 |  | \| \| |  | \| \| |  |  |  |  |  |  |  |  |
| 2 | Vitvi04g01440\_t001 |  | Vitvi18g01092\_t001.2.6037826c |  | \| \| |  |  |  |  |  |  |  |  |
| 2 | Vitvi04g01441\_t001 |  | Vitvi18g01093\_t001 |  | Vitvi03g00064\_t001 |  |  |  |  |  |  |  |  |
| 2 | Vitvi04g01442\_t001 |  | \| \| |  | Vitvi03g00065\_t001 |  |  |  |  |  |  |  |  |
| 1 | Vitvi04g01443\_t001 |  | \| \| |  |  |  |  |  |  |  |  |  |  |
| 1 | Vitvi04g01444\_t001 |  | Vitvi18g01098\_t001 |  |  |  |  |  |  |  |  |  |  |
| 1 | Vitvi04g01445\_t001 |  | \| \| |  |  |  |  |  |  |  |  |  |  |
| 1 | Vitvi04g01446\_t001 |  | \| \| |  |  |  |  |  |  |  |  |  |  |
| 1 | Vitvi04g01447\_t001 |  | \| \| |  |  |  |  |  |  |  |  |  |  |
| 1 | Vitvi04g02148\_t001 |  | \| \| |  |  |  |  |  |  |  |  |  |  |
| 1 | Vitvi04g01448\_t001 |  | \| \| |  |  |  |  |  |  |  |  |  |  |
| 1 | Vitvi04g01449\_t001 |  | \| \| |  |  |  |  |  |  |  |  |  |  |
| 1 | Vitvi04g01450\_t002 |  | \| \| |  |  |  |  |  |  |  |  |  |  |
| 1 | Vitvi04g02149\_t001 |  | \| \| |  |  |  |  |  |  |  |  |  |  |
| 1 | Vitvi04g02150\_t001 |  | Vitvi18g02792\_t001 |  |  |  |  |  |  |  |  |  |  |
| 1 | Vitvi04g01451\_t001 |  | Vitvi18g02794\_t001 |  |  |  |  |  |  |  |  |  |  |
| 1 | Vitvi04g01452\_t001 |  | \| \| |  |  |  |  |  |  |  |  |  |  |
| 1 | Vitvi04g01453\_t001 |  | \| \| |  |  |  |  |  |  |  |  |  |  |
| 1 | Vitvi04g01454\_t001 |  | Vitvi18g01119\_t001 |  |  |  |  |  |  |  |  |  |  |
| 1 | Vitvi04g01456\_t001 |  | \| \| |  |  |  |  |  |  |  |  |  |  |
| 1 | Vitvi04g04388\_t001 |  | \| \| |  |  |  |  |  |  |  |  |  |  |
| 1 | Vitvi04g01462\_t001 |  | \| \| |  |  |  |  |  |  |  |  |  |  |
| 1 | Vitvi04g01463\_t001 |  | \| \| |  |  |  |  |  |  |  |  |  |  |
| 1 | Vitvi04g01464\_t001 |  | \| \| |  |  |  |  |  |  |  |  |  |  |
| 1 | Vitvi04g02153\_t001 |  | \| \| |  |  |  |  |  |  |  |  |  |  |
| 1 | Vitvi04g04389\_t001 |  | \| \| |  |  |  |  |  |  |  |  |  |  |
| 2 | Vitvi04g01466\_t001 |  | Vitvi18g01129\_t001 |  | Vitvi03g00119\_t001 |  |  |  |  |  |  |  |  |
| 2 | Vitvi04g01467\_t001 |  | \| \| |  | Vitvi03g00120\_t001 |  |  |  |  |  |  |  |  |
| 2 | Vitvi04g04390\_t001 |  | \| \| |  | \| \| |  |  |  |  |  |  |  |  |
| 2 | Vitvi04g01468\_t001 |  | Vitvi18g01130\_t001 |  | \| \| |  |  |  |  |  |  |  |  |
| 2 | Vitvi04g01469\_t001 |  | \| \| |  | Vitvi03g00121\_t001 |  |  |  |  |  |  |  |  |
| 2 | Vitvi04g01470\_t001 |  | Vitvi18g01131\_t001 |  | Vitvi03g00123\_t001 |  |  |  |  |  |  |  |  |
| 1 | Vitvi04g01471\_t001 |  |  |  | \| \| |  |  |  |  |  |  |  |  |
| 1 | Vitvi04g01472\_t001 |  |  |  | \| \| |  |  |  |  |  |  |  |  |
| 1 | Vitvi04g02157\_t001 |  |  |  | \| \| |  |  |  |  |  |  |  |  |
| 1 | Vitvi04g01473\_t001 |  |  |  | \| \| |  |  |  |  |  |  |  |  |
| 1 | Vitvi04g01474\_t001 |  |  |  | \| \| |  |  |  |  |  |  |  |  |
| 1 | Vitvi04g02158\_t001 |  |  |  | \| \| |  |  |  |  |  |  |  |  |
| 1 | Vitvi04g02159\_t001 |  |  |  | \| \| |  |  |  |  |  |  |  |  |
| 1 | Vitvi04g02160\_t002 |  |  |  | \| \| |  |  |  |  |  |  |  |  |
| 1 | Vitvi04g01475\_t002 |  |  |  | \| \| |  |  |  |  |  |  |  |  |
| 1 | Vitvi04g01476\_t001 |  |  |  | \| \| |  |  |  |  |  |  |  |  |
| 1 | Vitvi04g01477\_t002 |  |  |  | \| \| |  |  |  |  |  |  |  |  |
| 1 | Vitvi04g01481\_t001 |  |  |  | \| \| |  |  |  |  |  |  |  |  |
| 1 | Vitvi04g02162\_t001 |  |  |  | \| \| |  |  |  |  |  |  |  |  |
| 1 | Vitvi04g01482\_t001 |  |  |  | Vitvi03g00133\_t001 |  |  |  |  |  |  |  |  |
| 1 | Vitvi04g01483\_t001 |  |  |  | \| \| |  |  |  |  |  |  |  |  |
| 1 | Vitvi04g01484\_t001 |  |  |  | Vitvi03g00135\_t001 |  |  |  |  |  |  |  |  |
| 1 | Vitvi04g01485\_t001 |  |  |  | \| \| |  |  |  |  |  |  |  |  |
| 1 | Vitvi04g01486\_t001 |  |  |  | Vitvi03g00136\_t001 |  |  |  |  |  |  |  |  |
| 1 | Vitvi04g02163\_t001 |  |  |  | \| \| |  |  |  |  |  |  |  |  |
| 1 | Vitvi04g04391\_t001 |  |  |  | Vitvi03g00137\_t001 |  |  |  |  |  |  |  |  |
| 1 | Vitvi04g04392\_t001 |  |  |  | \| \| |  |  |  |  |  |  |  |  |
| 1 | Vitvi04g02165\_t001 |  |  |  | \| \| |  |  |  |  |  |  |  |  |
| 1 | Vitvi04g02166\_t001 |  |  |  | \| \| |  |  |  |  |  |  |  |  |
| 1 | Vitvi04g01488\_t001 |  |  |  | \| \| |  |  |  |  |  |  |  |  |
| 1 | Vitvi04g01490\_t001 |  |  |  | \| \| |  |  |  |  |  |  |  |  |
| 1 | Vitvi04g02167\_t001 |  |  |  | \| \| |  |  |  |  |  |  |  |  |
| 1 | Vitvi04g02168\_t001 |  |  |  | \| \| |  |  |  |  |  |  |  |  |
| 1 | Vitvi04g02169\_t001 |  |  |  | \| \| |  |  |  |  |  |  |  |  |
| 1 | Vitvi04g01492\_t001 |  |  |  | \| \| |  |  |  |  |  |  |  |  |
| 1 | Vitvi04g01493\_t001 |  |  |  | Vitvi03g00139\_t002 |  |  |  |  |  |  |  |  |
| 1 | Vitvi04g04393\_t001 |  |  |  | \| \| |  |  |  |  |  |  |  |  |
| 1 | Vitvi04g04394\_t001 |  |  |  | Vitvi03g00141\_t001 |  |  |  |  |  |  |  |  |
| 1 | Vitvi04g01495\_t001 |  |  |  | \| \| |  |  |  |  |  |  |  |  |
| 1 | Vitvi04g01497\_t001 |  |  |  | \| \| |  |  |  |  |  |  |  |  |
| 1 | Vitvi04g04395\_t001 |  |  |  | \| \| |  |  |  |  |  |  |  |  |
| 2 | Vitvi04g01498\_t001 |  | Vitvi18g01149\_t001 |  | Vitvi03g00143\_t001 |  |  |  |  |  |  |  |  |
| 2 | Vitvi04g01499\_t001 |  | Vitvi18g01150\_t001 |  | \| \| |  |  |  |  |  |  |  |  |
| 2 | Vitvi04g01500\_t001 |  | Vitvi18g02814\_t002 |  | Vitvi03g01400\_t001 |  |  |  |  |  |  |  |  |
| 2 | Vitvi04g02170\_t001 |  | \| \| |  | Vitvi03g00148\_t001 |  |  |  |  |  |  |  |  |
| 2 | Vitvi04g04396\_t001 |  | Vitvi18g01157\_t001 |  | \| \| |  |  |  |  |  |  |  |  |
| 2 | Vitvi04g04397\_t001 |  | \| \| |  | \| \| |  |  |  |  |  |  |  |  |
| 2 | Vitvi04g01503\_t001 |  | \| \| |  | \| \| |  |  |  |  |  |  |  |  |
| 2 | Vitvi04g04398\_t001 |  | Vitvi18g01161\_t001 |  | Vitvi03g00152\_t001 |  |  |  |  |  |  |  |  |
| 2 | Vitvi04g04399\_t001 |  | \| \| |  | \| \| |  |  |  |  |  |  |  |  |
| 2 | Vitvi04g04400\_t001 |  | \| \| |  | \| \| |  |  |  |  |  |  |  |  |
| 2 | Vitvi04g02174\_t001 |  | \| \| |  | \| \| |  |  |  |  |  |  |  |  |
| 2 | Vitvi04g01505\_t001 |  | Vitvi18g01165\_t001 |  | \| \| |  |  |  |  |  |  |  |  |
| 2 | Vitvi04g01506\_t001 |  | Vitvi18g01166\_t001 |  | \| \| |  |  |  |  |  |  |  |  |
| 1 | Vitvi04g01542\_t001 |  |  |  | \| \| |  |  |  |  |  |  |  |  |
| 1 | Vitvi04g01543\_t001.1.6037826e |  |  |  | \| \| |  |  |  |  |  |  |  |  |
| 2 | Vitvi04g02306\_t001 |  | Vitvi18g00190\_t001 |  | \| \| |  |  |  |  |  |  |  |  |
| 2 | Vitvi04g01545\_t001 |  | \| \| |  | \| \| |  |  |  |  |  |  |  |  |
| 2 | Vitvi04g04401\_t001 |  | \| \| |  | \| \| |  |  |  |  |  |  |  |  |
| 2 | Vitvi04g02276\_t001 |  | \| \| |  | \| \| |  |  |  |  |  |  |  |  |
| 2 | Vitvi04g02277\_t001 |  | \| \| |  | \| \| |  |  |  |  |  |  |  |  |
| 2 | Vitvi04g02278\_t001 |  | \| \| |  | \| \| |  |  |  |  |  |  |  |  |
| 2 | Vitvi04g02280\_t001 |  | \| \| |  | Vitvi03g00167\_t001 |  |  |  |  |  |  |  |  |
| 2 | Vitvi04g04402\_t001 |  | \| \| |  | Vitvi03g00168\_t001 |  |  |  |  |  |  |  |  |
| 2 | Vitvi04g01537\_t001 |  | \| \| |  | \| \| |  |  |  |  |  |  |  |  |
| 2 | Vitvi04g01536\_t001 |  | \| \| |  | \| \| |  |  |  |  |  |  |  |  |
| 2 | Vitvi04g01535\_t001 |  | \| \| |  | \| \| |  |  |  |  |  |  |  |  |
| 2 | Vitvi04g02185\_t001 |  | Vitvi18g00172\_t001.3.6037826c |  | \| \| |  |  |  |  |  |  |  |  |
| 2 | Vitvi04g02184\_t001 |  | \| \| |  | \| \| |  |  |  |  |  |  |  |  |
| 2 | Vitvi04g01534\_t001 |  | \| \| |  | \| \| |  |  |  |  |  |  |  |  |
| 2 | Vitvi04g04403\_t001 |  | Vitvi18g00167\_t001 |  | \| \| |  |  |  |  |  |  |  |  |
| 2 | Vitvi04g04404\_t001 |  | \| \| |  | \| \| |  |  |  |  |  |  |  |  |
| 2 | Vitvi04g04405\_t001 |  | Vitvi18g00164\_t001 |  | \| \| |  |  |  |  |  |  |  |  |
| 2 | Vitvi04g01530\_t002 |  | Vitvi18g00159\_t001 |  | \| \| |  |  |  |  |  |  |  |  |
| 2 | Vitvi04g01529\_t001 |  | \| \| |  | \| \| |  |  |  |  |  |  |  |  |
| 2 | Vitvi04g01527\_t001 |  | \| \| |  | \| \| |  |  |  |  |  |  |  |  |
| 2 | Vitvi04g04406\_t001 |  | \| \| |  | \| \| |  |  |  |  |  |  |  |  |
| 2 | Vitvi04g01526\_t001 |  | \| \| |  | \| \| |  |  |  |  |  |  |  |  |
| 2 | Vitvi04g04407\_t002 |  | \| \| |  | \| \| |  |  |  |  |  |  |  |  |
| 2 | Vitvi04g04408\_t001 |  | \| \| |  | \| \| |  |  |  |  |  |  |  |  |
| 2 | Vitvi04g04409\_t001 |  | Vitvi18g00154\_t001 |  | \| \| |  |  |  |  |  |  |  |  |
| 2 | Vitvi04g02295\_t001 |  | \| \| |  | \| \| |  |  |  |  |  |  |  |  |
| 2 | Vitvi04g04410\_t001 |  | \| \| |  | \| \| |  |  |  |  |  |  |  |  |
| 2 | Vitvi04g04411\_t001 |  | \| \| |  | \| \| |  |  |  |  |  |  |  |  |
| 2 | Vitvi04g04412\_t001 |  | \| \| |  | \| \| |  |  |  |  |  |  |  |  |
| 2 | Vitvi04g02288\_t001 |  | \| \| |  | \| \| |  |  |  |  |  |  |  |  |
| 2 | Vitvi04g04413\_t001 |  | \| \| |  | \| \| |  |  |  |  |  |  |  |  |
| 2 | Vitvi04g01519\_t001 |  | Vitvi18g00147\_t001 |  | Vitvi03g00183\_t001 |  |  |  |  |  |  |  |  |
| 2 | Vitvi04g02178\_t001 |  | \| \| |  | \| \| |  |  |  |  |  |  |  |  |
| 2 | Vitvi04g02179\_t001 |  | \| \| |  | \| \| |  |  |  |  |  |  |  |  |
| 2 | Vitvi04g01520\_t001 |  | \| \| |  | \| \| |  |  |  |  |  |  |  |  |
| 2 | Vitvi04g04414\_t001 |  | \| \| |  | \| \| |  |  |  |  |  |  |  |  |
| 2 | Vitvi04g04415\_t001 |  | \| \| |  | \| \| |  |  |  |  |  |  |  |  |
| 2 | Vitvi04g01521\_t001 |  | \| \| |  | \| \| |  |  |  |  |  |  |  |  |
| 2 | Vitvi04g04416\_t001 |  | \| \| |  | \| \| |  |  |  |  |  |  |  |  |
| 2 | Vitvi04g04417\_t001 |  | \| \| |  | \| \| |  |  |  |  |  |  |  |  |
| 2 | Vitvi04g01541\_t001 |  | \| \| |  | \| \| |  |  |  |  |  |  |  |  |
| 2 | Vitvi04g01540\_t001 |  | \| \| |  | \| \| |  |  |  |  |  |  |  |  |
| 2 | Vitvi04g02187\_t001 |  | \| \| |  | \| \| |  |  |  |  |  |  |  |  |
| 2 | Vitvi04g04418\_t001 |  | \| \| |  | Vitvi03g01416\_t001 |  |  |  |  |  |  |  |  |
| 2 | Vitvi04g02297\_t001 |  | \| \| |  | \| \| |  |  |  |  |  |  |  |  |
| 2 | Vitvi04g02298\_t001 |  | Vitvi18g00122\_t001 |  | \| \| |  |  |  |  |  |  |  |  |
| 2 | Vitvi04g02299\_t001 |  | \| \| |  | \| \| |  |  |  |  |  |  |  |  |
| 2 | Vitvi04g04419\_t001 |  | \| \| |  | \| \| |  |  |  |  |  |  |  |  |
| 2 | Vitvi04g04420\_t001 |  | Vitvi18g00118\_t001 |  | \| \| |  |  |  |  |  |  |  |  |
| 2 | Vitvi04g02314\_t001 |  | \| \| |  | \| \| |  |  |  |  |  |  |  |  |
| 2 | Vitvi04g04421\_t001 |  | \| \| |  | \| \| |  |  |  |  |  |  |  |  |
| 2 | Vitvi04g04422\_t001 |  | \| \| |  | \| \| |  |  |  |  |  |  |  |  |
| 2 | Vitvi04g04423\_t001 |  | \| \| |  | \| \| |  |  |  |  |  |  |  |  |
| 2 | Vitvi04g04424\_t001 |  | \| \| |  | \| \| |  |  |  |  |  |  |  |  |
| 2 | Vitvi04g04425\_t001 |  | \| \| |  | \| \| |  |  |  |  |  |  |  |  |
| 2 | Vitvi04g04426\_t001 |  | \| \| |  | \| \| |  |  |  |  |  |  |  |  |
| 2 | Vitvi04g04427\_t001 |  | \| \| |  | \| \| |  |  |  |  |  |  |  |  |
| 2 | Vitvi04g04428\_t001 |  | \| \| |  | \| \| |  |  |  |  |  |  |  |  |
| 2 | Vitvi04g04429\_t001 |  | \| \| |  | Vitvi03g00199\_t003 |  |  |  |  |  |  |  |  |
| 2 | Vitvi04g04430\_t001 |  | \| \| |  | \| \| |  |  |  |  |  |  |  |  |
| 2 | Vitvi04g01518\_t001 |  | Vitvi18g00105\_t001 |  | \| \| |  |  |  |  |  |  |  |  |
| 1 | Vitvi04g02300\_t001 |  |  |  | Vitvi03g00206\_t001 |  |  |  |  |  |  |  |  |
| 1 | Vitvi04g04431\_t001 |  |  |  | \| \| |  |  |  |  |  |  |  |  |
| 1 | Vitvi04g04432\_t001 |  |  |  | \| \| |  |  |  |  |  |  |  |  |
| 1 | Vitvi04g04433\_t001 |  |  |  | \| \| |  |  |  |  |  |  |  |  |
| 1 | Vitvi04g04434\_t001 |  |  |  | \| \| |  |  |  |  |  |  |  |  |
| 1 | Vitvi04g01510\_t001 |  |  |  | \| \| |  |  |  |  |  |  |  |  |
| 1 | Vitvi04g01511\_t001 |  |  |  | Vitvi03g00207\_t001 |  |  |  |  |  |  |  |  |
| 0 | Vitvi04g04435\_t001 |  |  |  |  |  |  |  |  |  |  |  |  |
| 0 | Vitvi04g04436\_t001 |  |  |  |  |  |  |  |  |  |  |  |  |
| 0 | Vitvi04g04437\_t001 |  |  |  |  |  |  |  |  |  |  |  |  |
| 0 | Vitvi04g02190\_t001 |  |  |  |  |  |  |  |  |  |  |  |  |
| 0 | Vitvi04g04438\_t001 |  |  |  |  |  |  |  |  |  |  |  |  |
| 0 | Vitvi04g01550\_t001 |  |  |  |  |  |  |  |  |  |  |  |  |
| 0 | Vitvi04g04439\_t001 |  |  |  |  |  |  |  |  |  |  |  |  |
| 0 | Vitvi04g02198\_t001 |  |  |  |  |  |  |  |  |  |  |  |  |
| 0 | Vitvi04g04440\_t001 |  |  |  |  |  |  |  |  |  |  |  |  |
| 0 | Vitvi04g04441\_t001 |  |  |  |  |  |  |  |  |  |  |  |  |
| 1 | Vitvi04g01554\_t001 |  | Vitvi18g02494\_t001 |  |  |  |  |  |  |  |  |  |  |
| 1 | Vitvi04g01555\_t001 |  | Vitvi18g02493\_t001 |  |  |  |  |  |  |  |  |  |  |
| 1 | Vitvi04g01556\_t001 |  | \| \| |  |  |  |  |  |  |  |  |  |  |
| 1 | Vitvi04g01557\_t001 |  | \| \| |  |  |  |  |  |  |  |  |  |  |
| 1 | Vitvi04g01558\_t001 |  | \| \| |  |  |  |  |  |  |  |  |  |  |
| 1 | Vitvi04g01559\_t002 |  | \| \| |  |  |  |  |  |  |  |  |  |  |
| 1 | Vitvi04g04442\_t001 |  | \| \| |  |  |  |  |  |  |  |  |  |  |
| 1 | Vitvi04g04443\_t001 |  | \| \| |  |  |  |  |  |  |  |  |  |  |
| 1 | Vitvi04g04444\_t001 |  | \| \| |  |  |  |  |  |  |  |  |  |  |
| 1 | Vitvi04g04445\_t001 |  | \| \| |  |  |  |  |  |  |  |  |  |  |
| 1 | Vitvi04g04446\_t001 |  | \| \| |  |  |  |  |  |  |  |  |  |  |
| 1 | Vitvi04g02199\_t001 |  | \| \| |  |  |  |  |  |  |  |  |  |  |
| 1 | Vitvi04g02200\_t001 |  | \| \| |  |  |  |  |  |  |  |  |  |  |
| 1 | Vitvi04g04447\_t001 |  | \| \| |  |  |  |  |  |  |  |  |  |  |
| 1 | Vitvi04g04448\_t001 |  | Vitvi18g00095\_t001 |  |  |  |  |  |  |  |  |  |  |
| 1 | Vitvi04g01565\_t001 |  | \| \| |  |  |  |  |  |  |  |  |  |  |
| 1 | Vitvi04g01566\_t001 |  | \| \| |  |  |  |  |  |  |  |  |  |  |
| 1 | Vitvi04g04449\_t001 |  | \| \| |  |  |  |  |  |  |  |  |  |  |
| 1 | Vitvi04g04450\_t001 |  | \| \| |  |  |  |  |  |  |  |  |  |  |
| 1 | Vitvi04g04451\_t001 |  | \| \| |  |  |  |  |  |  |  |  |  |  |
| 1 | Vitvi04g01568\_t001 |  | Vitvi18g00091\_t002 |  |  |  |  |  |  |  |  |  |  |
| 1 | Vitvi04g04452\_t001 |  | \| \| |  |  |  |  |  |  |  |  |  |  |
| 1 | Vitvi04g01570\_t001 |  | \| \| |  |  |  |  |  |  |  |  |  |  |
| 1 | Vitvi04g01571\_t001 |  | \| \| |  |  |  |  |  |  |  |  |  |  |
| 1 | Vitvi04g01572\_t001 |  | \| \| |  |  |  |  |  |  |  |  |  |  |
| 1 | Vitvi04g04453\_t001 |  | \| \| |  |  |  |  |  |  |  |  |  |  |
| 1 | Vitvi04g02201\_t001 |  | \| \| |  |  |  |  |  |  |  |  |  |  |
| 1 | Vitvi04g04454\_t001 |  | \| \| |  |  |  |  |  |  |  |  |  |  |
| 1 | Vitvi04g01573\_t001 |  | \| \| |  |  |  |  |  |  |  |  |  |  |
| 1 | Vitvi04g01574\_t001 |  | \| \| |  |  |  |  |  |  |  |  |  |  |
| 1 | Vitvi04g02203\_t004 |  | Vitvi18g02486\_t001 |  |  |  |  |  |  |  |  |  |  |
| 1 | Vitvi04g01575\_t001 |  | \| \| |  |  |  |  |  |  |  |  |  |  |
| 1 | Vitvi04g01576\_t001 |  | \| \| |  |  |  |  |  |  |  |  |  |  |
| 1 | Vitvi04g04455\_t001 |  | \| \| |  |  |  |  |  |  |  |  |  |  |
| 1 | Vitvi04g01577\_t001 |  | \| \| |  |  |  |  |  |  |  |  |  |  |
| 1 | Vitvi04g01578\_t001 |  | Vitvi18g00084\_t001 |  |  |  |  |  |  |  |  |  |  |
| 0 | Vitvi04g01579\_t002 |  |  |  |  |  |  |  |  |  |  |  |  |
| 0 | Vitvi04g01580\_t001 |  |  |  |  |  |  |  |  |  |  |  |  |
| 1 | Vitvi04g01581\_t001 |  | Vitvi18g02972\_t001 |  |  |  |  |  |  |  |  |  |  |
| 1 | Vitvi04g01582\_t001 |  | \| \| |  |  |  |  |  |  |  |  |  |  |
| 1 | Vitvi04g01583\_t001 |  | Vitvi18g01631\_t001 |  |  |  |  |  |  |  |  |  |  |
| 1 | Vitvi04g04456\_t001 |  | \| \| |  |  |  |  |  |  |  |  |  |  |
| 1 | Vitvi04g01584\_t001 |  | \| \| |  |  |  |  |  |  |  |  |  |  |
| 1 | Vitvi04g01585\_t001 |  | \| \| |  |  |  |  |  |  |  |  |  |  |
| 1 | Vitvi04g04457\_t001 |  | \| \| |  |  |  |  |  |  |  |  |  |  |
| 1 | Vitvi04g01586\_t001 |  | \| \| |  |  |  |  |  |  |  |  |  |  |
| 1 | Vitvi04g02205\_t001 |  | \| \| |  |  |  |  |  |  |  |  |  |  |
| 1 | Vitvi04g04458\_t001 |  | \| \| |  |  |  |  |  |  |  |  |  |  |
| 1 | Vitvi04g01589\_t001 |  | \| \| |  |  |  |  |  |  |  |  |  |  |
| 1 | Vitvi04g04459\_t001 |  | \| \| |  |  |  |  |  |  |  |  |  |  |
| 1 | Vitvi04g04460\_t001 |  | \| \| |  |  |  |  |  |  |  |  |  |  |
| 1 | Vitvi04g04461\_t001 |  | \| \| |  |  |  |  |  |  |  |  |  |  |
| 1 | Vitvi04g02210\_t001 |  | \| \| |  |  |  |  |  |  |  |  |  |  |
| 1 | Vitvi04g01590\_t001 |  | \| \| |  |  |  |  |  |  |  |  |  |  |
| 1 | Vitvi04g04462\_t001 |  | \| \| |  |  |  |  |  |  |  |  |  |  |
| 1 | Vitvi04g01591\_t005 |  | Vitvi18g01634\_t002 |  |  |  |  |  |  |  |  |  |  |
| 1 | Vitvi04g02211\_t001 |  | Vitvi18g01636\_t001 |  |  |  |  |  |  |  |  |  |  |
| 1 | Vitvi04g01592\_t001 |  | \| \| |  |  |  |  |  |  |  |  |  |  |
| 1 | Vitvi04g01595\_t001 |  | \| \| |  |  |  |  |  |  |  |  |  |  |
| 1 | Vitvi04g01596\_t001 |  | Vitvi18g01648\_t001 |  |  |  |  |  |  |  |  |  |  |
| 1 | Vitvi04g01597\_t002 |  | \| \| |  |  |  |  |  |  |  |  |  |  |
| 1 | Vitvi04g04463\_t001 |  | \| \| |  |  |  |  |  |  |  |  |  |  |
| 1 | Vitvi04g01598\_t001 |  | \| \| |  |  |  |  |  |  |  |  |  |  |
| 1 | Vitvi04g04464\_t001 |  | \| \| |  |  |  |  |  |  |  |  |  |  |
| 1 | Vitvi04g04465\_t001 |  | \| \| |  |  |  |  |  |  |  |  |  |  |
| 1 | Vitvi04g02214\_t001 |  | \| \| |  |  |  |  |  |  |  |  |  |  |
| 1 | Vitvi04g02216\_t001 |  | \| \| |  |  |  |  |  |  |  |  |  |  |
| 1 | Vitvi04g02219\_t001 |  | \| \| |  |  |  |  |  |  |  |  |  |  |
| 1 | Vitvi04g04466\_t001 |  | \| \| |  |  |  |  |  |  |  |  |  |  |
| 1 | Vitvi04g01599\_t001 |  | \| \| |  |  |  |  |  |  |  |  |  |  |
| 1 | Vitvi04g02221\_t001 |  | \| \| |  |  |  |  |  |  |  |  |  |  |
| 1 | Vitvi04g04467\_t001 |  | \| \| |  |  |  |  |  |  |  |  |  |  |
| 1 | Vitvi04g04468\_t001 |  | \| \| |  |  |  |  |  |  |  |  |  |  |
| 1 | Vitvi04g02222\_t001 |  | \| \| |  |  |  |  |  |  |  |  |  |  |
| 1 | Vitvi04g04469\_t001 |  | \| \| |  |  |  |  |  |  |  |  |  |  |
| 1 | Vitvi04g01601\_t001 |  | \| \| |  |  |  |  |  |  |  |  |  |  |
| 1 | Vitvi04g04470\_t001 |  | \| \| |  |  |  |  |  |  |  |  |  |  |
| 1 | Vitvi04g01602\_t001 |  | \| \| |  |  |  |  |  |  |  |  |  |  |
| 1 | Vitvi04g04471\_t001 |  | \| \| |  |  |  |  |  |  |  |  |  |  |
| 1 | Vitvi04g01604\_t001 |  | Vitvi18g01649\_t001 |  |  |  |  |  |  |  |  |  |  |
| 1 | Vitvi04g04472\_t001 |  | \| \| |  |  |  |  |  |  |  |  |  |  |
| 1 | Vitvi04g04473\_t001 |  | \| \| |  |  |  |  |  |  |  |  |  |  |
| 1 | Vitvi04g01608\_t001 |  | \| \| |  |  |  |  |  |  |  |  |  |  |
| 1 | Vitvi04g01610\_t001 |  | \| \| |  |  |  |  |  |  |  |  |  |  |
| 1 | Vitvi04g01611\_t001 |  | \| \| |  |  |  |  |  |  |  |  |  |  |
| 1 | Vitvi04g04474\_t001 |  | Vitvi18g04427\_t001 |  |  |  |  |  |  |  |  |  |  |
| 0 | Vitvi04g04475\_t001 |  |  |  |  |  |  |  |  |  |  |  |  |
| 0 | Vitvi04g04476\_t001 |  |  |  |  |  |  |  |  |  |  |  |  |
| 0 | Vitvi04g04477\_t001 |  |  |  |  |  |  |  |  |  |  |  |  |
| 0 | Vitvi04g01613\_t001 |  |  |  |  |  |  |  |  |  |  |  |  |
| 0 | Vitvi04g04478\_t001 |  |  |  |  |  |  |  |  |  |  |  |  |
| 0 | Vitvi04g04479\_t001 |  |  |  |  |  |  |  |  |  |  |  |  |
| 0 | Vitvi04g01616\_t001 |  |  |  |  |  |  |  |  |  |  |  |  |
| 0 | Vitvi04g01617\_t001 |  |  |  |  |  |  |  |  |  |  |  |  |
| 0 | Vitvi04g04480\_t001 |  |  |  |  |  |  |  |  |  |  |  |  |
| 0 | Vitvi04g01618\_t001 |  |  |  |  |  |  |  |  |  |  |  |  |
| 0 | Vitvi04g04481\_t001 |  |  |  |  |  |  |  |  |  |  |  |  |
| 0 | Vitvi04g01619\_t001 |  |  |  |  |  |  |  |  |  |  |  |  |
| 0 | Vitvi04g01620\_t001 |  |  |  |  |  |  |  |  |  |  |  |  |
| 0 | Vitvi04g04482\_t001 |  |  |  |  |  |  |  |  |  |  |  |  |
| 0 | Vitvi04g04483\_t001 |  |  |  |  |  |  |  |  |  |  |  |  |
| 0 | Vitvi04g01622\_t001 |  |  |  |  |  |  |  |  |  |  |  |  |
| 0 | Vitvi04g01623\_t002 |  |  |  |  |  |  |  |  |  |  |  |  |
| 0 | Vitvi04g01624\_t001 |  |  |  |  |  |  |  |  |  |  |  |  |
| 0 | Vitvi04g01625\_t001 |  |  |  |  |  |  |  |  |  |  |  |  |
| 0 | Vitvi04g01626\_t001 |  |  |  |  |  |  |  |  |  |  |  |  |
| 0 | Vitvi04g04484\_t001 |  |  |  |  |  |  |  |  |  |  |  |  |
| 0 | Vitvi04g02225\_t001 |  |  |  |  |  |  |  |  |  |  |  |  |
| 0 | Vitvi04g02226\_t001 |  |  |  |  |  |  |  |  |  |  |  |  |
| 0 | Vitvi04g04485\_t001 |  |  |  |  |  |  |  |  |  |  |  |  |
| 0 | Vitvi04g04486\_t001 |  |  |  |  |  |  |  |  |  |  |  |  |
| 0 | Vitvi04g04487\_t001 |  |  |  |  |  |  |  |  |  |  |  |  |
| 0 | Vitvi04g04488\_t001 |  |  |  |  |  |  |  |  |  |  |  |  |
| 0 | Vitvi04g02230\_t001 |  |  |  |  |  |  |  |  |  |  |  |  |
| 0 | Vitvi04g01633\_t002 |  |  |  |  |  |  |  |  |  |  |  |  |
| 0 | Vitvi04g04489\_t001 |  |  |  |  |  |  |  |  |  |  |  |  |
| 0 | Vitvi04g01635\_t001 |  |  |  |  |  |  |  |  |  |  |  |  |
| 0 | Vitvi04g01636\_t001 |  |  |  |  |  |  |  |  |  |  |  |  |
| 0 | Vitvi04g04490\_t001 |  |  |  |  |  |  |  |  |  |  |  |  |
| 0 | Vitvi04g02232\_t001 |  |  |  |  |  |  |  |  |  |  |  |  |
| 0 | Vitvi04g04491\_t001 |  |  |  |  |  |  |  |  |  |  |  |  |
| 0 | Vitvi04g02233\_t001 |  |  |  |  |  |  |  |  |  |  |  |  |
| 0 | Vitvi04g04492\_t001 |  |  |  |  |  |  |  |  |  |  |  |  |
| 0 | Vitvi04g01637\_t001 |  |  |  |  |  |  |  |  |  |  |  |  |
| 0 | Vitvi04g01638\_t001 |  |  |  |  |  |  |  |  |  |  |  |  |
| 0 | Vitvi04g04493\_t001 |  |  |  |  |  |  |  |  |  |  |  |  |
| 0 | Vitvi04g04494\_t001 |  |  |  |  |  |  |  |  |  |  |  |  |
| 0 | Vitvi04g02237\_t001 |  |  |  |  |  |  |  |  |  |  |  |  |
| 0 | Vitvi04g04495\_t001 |  |  |  |  |  |  |  |  |  |  |  |  |
| 0 | Vitvi04g02239\_t001 |  |  |  |  |  |  |  |  |  |  |  |  |
| 0 | Vitvi04g02241\_t001 |  |  |  |  |  |  |  |  |  |  |  |  |
| 0 | Vitvi04g01640\_t001 |  |  |  |  |  |  |  |  |  |  |  |  |
| 0 | Vitvi04g01641\_t001 |  |  |  |  |  |  |  |  |  |  |  |  |
| 0 | Vitvi04g02242\_t001 |  |  |  |  |  |  |  |  |  |  |  |  |
| 0 | Vitvi04g04496\_t001 |  |  |  |  |  |  |  |  |  |  |  |  |
| 0 | Vitvi04g02243\_t001 |  |  |  |  |  |  |  |  |  |  |  |  |
| 0 | Vitvi04g04497\_t001 |  |  |  |  |  |  |  |  |  |  |  |  |
| 0 | Vitvi04g01643\_t001 |  |  |  |  |  |  |  |  |  |  |  |  |
| 0 | Vitvi04g01645\_t001 |  |  |  |  |  |  |  |  |  |  |  |  |
| 0 | Vitvi04g01646\_t001 |  |  |  |  |  |  |  |  |  |  |  |  |
| 0 | Vitvi04g01648\_t001 |  |  |  |  |  |  |  |  |  |  |  |  |
| 0 | Vitvi04g01650\_t001 |  |  |  |  |  |  |  |  |  |  |  |  |
| 0 | Vitvi04g01651\_t001 |  |  |  |  |  |  |  |  |  |  |  |  |
| 0 | Vitvi04g02245\_t001 |  |  |  |  |  |  |  |  |  |  |  |  |
| 0 | Vitvi04g01653\_t001 |  |  |  |  |  |  |  |  |  |  |  |  |
| 1 | Vitvi04g01654\_t001 |  | Vitvi18g01244\_t001 |  |  |  |  |  |  |  |  |  |  |
| 1 | Vitvi04g01655\_t001 |  | \| \| |  |  |  |  |  |  |  |  |  |  |
| 1 | Vitvi04g01657\_t001 |  | Vitvi18g01242\_t002 |  |  |  |  |  |  |  |  |  |  |
| 1 | Vitvi04g02246\_t001 |  | \| \| |  |  |  |  |  |  |  |  |  |  |
| 1 | Vitvi04g04498\_t001 |  | \| \| |  |  |  |  |  |  |  |  |  |  |
| 1 | Vitvi04g02247\_t002 |  | \| \| |  |  |  |  |  |  |  |  |  |  |
| 1 | Vitvi04g01659\_t001 |  | \| \| |  |  |  |  |  |  |  |  |  |  |
| 1 | Vitvi04g01661\_t001 |  | Vitvi18g01240\_t003 |  |  |  |  |  |  |  |  |  |  |
| 1 | Vitvi04g01662\_t001 |  | \| \| |  |  |  |  |  |  |  |  |  |  |
| 1 | Vitvi04g01663\_t001 |  | Vitvi18g01235\_t004 |  |  |  |  |  |  |  |  |  |  |
| 1 | Vitvi04g02248\_t001 |  | Vitvi18g02833\_t001 |  |  |  |  |  |  |  |  |  |  |
| 1 | Vitvi04g01664\_t001 |  | \| \| |  |  |  |  |  |  |  |  |  |  |
| 1 | Vitvi04g01665\_t002 |  | \| \| |  |  |  |  |  |  |  |  |  |  |
| 1 | Vitvi04g04499\_t001 |  | \| \| |  |  |  |  |  |  |  |  |  |  |
| 1 | Vitvi04g01666\_t001 |  | \| \| |  |  |  |  |  |  |  |  |  |  |
| 1 | Vitvi04g01670\_t001 |  | Vitvi18g01223\_t001 |  |  |  |  |  |  |  |  |  |  |
| 1 | Vitvi04g01671\_t001 |  | \| \| |  |  |  |  |  |  |  |  |  |  |
| 1 | Vitvi04g01672\_t001 |  | \| \| |  |  |  |  |  |  |  |  |  |  |
| 1 | Vitvi04g04500\_t001 |  | \| \| |  |  |  |  |  |  |  |  |  |  |
| 1 | Vitvi04g04501\_t001 |  | \| \| |  |  |  |  |  |  |  |  |  |  |
| 1 | Vitvi04g01674\_t001 |  | \| \| |  |  |  |  |  |  |  |  |  |  |
| 1 | Vitvi04g04502\_t001 |  | \| \| |  |  |  |  |  |  |  |  |  |  |
| 1 | Vitvi04g01676\_t001 |  | \| \| |  |  |  |  |  |  |  |  |  |  |
| 1 | Vitvi04g01677\_t002 |  | \| \| |  |  |  |  |  |  |  |  |  |  |
| 1 | Vitvi04g01678\_t001 |  | \| \| |  |  |  |  |  |  |  |  |  |  |
| 1 | Vitvi04g01679\_t001 |  | \| \| |  |  |  |  |  |  |  |  |  |  |
| 1 | Vitvi04g01680\_t001 |  | \| \| |  |  |  |  |  |  |  |  |  |  |
| 1 | Vitvi04g04503\_t001 |  | \| \| |  |  |  |  |  |  |  |  |  |  |
| 1 | Vitvi04g01681\_t001 |  | \| \| |  |  |  |  |  |  |  |  |  |  |
| 1 | Vitvi04g04504\_t001 |  | \| \| |  |  |  |  |  |  |  |  |  |  |
| 1 | Vitvi04g01682\_t001 |  | \| \| |  |  |  |  |  |  |  |  |  |  |
| 1 | Vitvi04g01684\_t001 |  | \| \| |  |  |  |  |  |  |  |  |  |  |
| 1 | Vitvi04g01685\_t001 |  | \| \| |  |  |  |  |  |  |  |  |  |  |
| 1 | Vitvi04g04505\_t001 |  | Vitvi18g01214\_t001 |  |  |  |  |  |  |  |  |  |  |
| 1 | Vitvi04g04506\_t001 |  | \| \| |  |  |  |  |  |  |  |  |  |  |
| 1 | Vitvi04g01689\_t001 |  | \| \| |  |  |  |  |  |  |  |  |  |  |
| 1 | Vitvi04g04507\_t001 |  | \| \| |  |  |  |  |  |  |  |  |  |  |
| 1 | Vitvi04g01691\_t001 |  | \| \| |  |  |  |  |  |  |  |  |  |  |
| 1 | Vitvi04g01692\_t001 |  | \| \| |  |  |  |  |  |  |  |  |  |  |
| 1 | Vitvi04g01693\_t002 |  | \| \| |  |  |  |  |  |  |  |  |  |  |
| 1 | Vitvi04g01694\_t001 |  | \| \| |  |  |  |  |  |  |  |  |  |  |
| 1 | Vitvi04g01695\_t001 |  | \| \| |  |  |  |  |  |  |  |  |  |  |
| 1 | Vitvi04g02250\_t001 |  | \| \| |  |  |  |  |  |  |  |  |  |  |
| 1 | Vitvi04g01696\_t001 |  | Vitvi18g01210\_t001 |  |  |  |  |  |  |  |  |  |  |
| 1 | Vitvi04g01697\_t001 |  | Vitvi18g01209\_t001 |  |  |  |  |  |  |  |  |  |  |
| 1 | Vitvi04g01698\_t001 |  | \| \| |  |  |  |  |  |  |  |  |  |  |
| 1 | Vitvi04g01699\_t002 |  | \| \| |  |  |  |  |  |  |  |  |  |  |
| 1 | Vitvi04g01700\_t001 |  | \| \| |  |  |  |  |  |  |  |  |  |  |
| 1 | Vitvi04g01701\_t001 |  | \| \| |  |  |  |  |  |  |  |  |  |  |
| 1 | Vitvi04g04508\_t001 |  | \| \| |  |  |  |  |  |  |  |  |  |  |
| 1 | Vitvi04g01702\_t001 |  | Vitvi18g02823\_t001 |  |  |  |  |  |  |  |  |  |  |
| 1 | Vitvi04g01705\_t001 |  | \| \| |  |  |  |  |  |  |  |  |  |  |
| 1 | Vitvi04g01706\_t001 |  | \| \| |  |  |  |  |  |  |  |  |  |  |
| 1 | Vitvi04g01708\_t001 |  | \| \| |  |  |  |  |  |  |  |  |  |  |
| 1 | Vitvi04g01709\_t002 |  | \| \| |  |  |  |  |  |  |  |  |  |  |
| 1 | Vitvi04g01711\_t001 |  | Vitvi18g01174\_t001 |  |  |  |  |  |  |  |  |  |  |
| 0 | Vitvi04g04509\_t001 |  |  |  |  |  |  |  |  |  |  |  |  |
| 0 | Vitvi04g01714\_t001 |  |  |  |  |  |  |  |  |  |  |  |  |
| 0 | Vitvi04g01715\_t001 |  |  |  |  |  |  |  |  |  |  |  |  |
| 0 | Vitvi04g01719\_t001 |  |  |  |  |  |  |  |  |  |  |  |  |
| 0 | Vitvi04g01720\_t001 |  |  |  |  |  |  |  |  |  |  |  |  |
| 0 | Vitvi04g04510\_t001 |  |  |  |  |  |  |  |  |  |  |  |  |
| 0 | Vitvi04g01721\_t001 |  |  |  |  |  |  |  |  |  |  |  |  |
| 0 | Vitvi04g01722\_t001 |  |  |  |  |  |  |  |  |  |  |  |  |
| 0 | Vitvi04g01723\_t001 |  |  |  |  |  |  |  |  |  |  |  |  |
| 0 | Vitvi04g01724\_t001 |  |  |  |  |  |  |  |  |  |  |  |  |
| 0 | Vitvi04g01726\_t001 |  |  |  |  |  |  |  |  |  |  |  |  |
| 0 | Vitvi04g01727\_t001 |  |  |  |  |  |  |  |  |  |  |  |  |
| 0 | Vitvi04g01728\_t001 |  |  |  |  |  |  |  |  |  |  |  |  |
| 0 | Vitvi04g01729\_t001 |  |  |  |  |  |  |  |  |  |  |  |  |
| 0 | Vitvi04g01730\_t001 |  |  |  |  |  |  |  |  |  |  |  |  |
| 0 | Vitvi04g01731\_t001 |  |  |  |  |  |  |  |  |  |  |  |  |
| 0 | Vitvi04g04511\_t001 |  |  |  |  |  |  |  |  |  |  |  |  |
| 0 | Vitvi04g01733\_t001 |  |  |  |  |  |  |  |  |  |  |  |  |
| 0 | Vitvi04g01734\_t001 |  |  |  |  |  |  |  |  |  |  |  |  |
| 0 | Vitvi04g01735\_t001 |  |  |  |  |  |  |  |  |  |  |  |  |
| 0 | Vitvi04g01736\_t001 |  |  |  |  |  |  |  |  |  |  |  |  |
| 0 | Vitvi04g01737\_t001 |  |  |  |  |  |  |  |  |  |  |  |  |
| 0 | Vitvi04g01738\_t001 |  |  |  |  |  |  |  |  |  |  |  |  |
| 0 | Vitvi04g01739\_t001 |  |  |  |  |  |  |  |  |  |  |  |  |
| 0 | Vitvi04g01740\_t001 |  |  |  |  |  |  |  |  |  |  |  |  |
| 0 | Vitvi04g01741\_t001 |  |  |  |  |  |  |  |  |  |  |  |  |
| 0 | Vitvi04g02252\_t001 |  |  |  |  |  |  |  |  |  |  |  |  |
| 0 | Vitvi04g01742\_t001 |  |  |  |  |  |  |  |  |  |  |  |  |
| 0 | Vitvi04g02253\_t001 |  |  |  |  |  |  |  |  |  |  |  |  |
| 0 | Vitvi04g04512\_t001 |  |  |  |  |  |  |  |  |  |  |  |  |
| 0 | Vitvi04g01743\_t001 |  |  |  |  |  |  |  |  |  |  |  |  |
| 0 | Vitvi04g01745\_t002 |  |  |  |  |  |  |  |  |  |  |  |  |
| 0 | Vitvi04g01746\_t003 |  |  |  |  |  |  |  |  |  |  |  |  |
| 0 | Vitvi04g01747\_t001 |  |  |  |  |  |  |  |  |  |  |  |  |
| 0 | Vitvi04g01748\_t001 |  |  |  |  |  |  |  |  |  |  |  |  |
| 0 | Vitvi04g04513\_t001 |  |  |  |  |  |  |  |  |  |  |  |  |
| 0 | Vitvi04g04514\_t001 |  |  |  |  |  |  |  |  |  |  |  |  |
| 0 | Vitvi04g01750\_t002 |  |  |  |  |  |  |  |  |  |  |  |  |
| 0 | Vitvi04g01751\_t001 |  |  |  |  |  |  |  |  |  |  |  |  |
| 0 | Vitvi04g04515\_t001 |  |  |  |  |  |  |  |  |  |  |  |  |
| 0 | Vitvi04g04516\_t001 |  |  |  |  |  |  |  |  |  |  |  |  |
| 0 | Vitvi04g04517\_t001 |  |  |  |  |  |  |  |  |  |  |  |  |
| 0 | Vitvi04g02258\_t001 |  |  |  |  |  |  |  |  |  |  |  |  |
| 0 | Vitvi04g02259\_t001 |  |  |  |  |  |  |  |  |  |  |  |  |
| 0 | Vitvi04g01754\_t001 |  |  |  |  |  |  |  |  |  |  |  |  |
| 0 | Vitvi04g02260\_t001 |  |  |  |  |  |  |  |  |  |  |  |  |
| 0 | Vitvi04g04518\_t001 |  |  |  |  |  |  |  |  |  |  |  |  |
| 0 | Vitvi04g04519\_t001 |  |  |  |  |  |  |  |  |  |  |  |  |
| 0 | Vitvi04g01756\_t001 |  |  |  |  |  |  |  |  |  |  |  |  |
| 0 | Vitvi04g01757\_t001 |  |  |  |  |  |  |  |  |  |  |  |  |
| 0 | Vitvi04g04520\_t001 |  |  |  |  |  |  |  |  |  |  |  |  |
| 0 | Vitvi04g01759\_t001 |  |  |  |  |  |  |  |  |  |  |  |  |
| 0 | Vitvi04g01760\_t001 |  |  |  |  |  |  |  |  |  |  |  |  |
| 0 | Vitvi04g04521\_t001 |  |  |  |  |  |  |  |  |  |  |  |  |
| 0 | Vitvi04g01763\_t001 |  |  |  |  |  |  |  |  |  |  |  |  |
| 0 | Vitvi04g02261\_t002 |  |  |  |  |  |  |  |  |  |  |  |  |
